# Adopting electric vertical takeoff and landing aircraft for green transport transformation

**DOI:** 10.1016/j.fmre.2025.04.002

**Published:** 2025-04-11

**Authors:** Ming Liu, Han Hao, Yong Geng, Hao Dou, Xin Sun, Fanlong Bai, Xiaobo Qu, Kai Wang, Zongwei Liu, Fuquan Zhao

**Affiliations:** a School of Vehicle and Mobility, Tsinghua University, Beijing 100084, China; b Energy Policy Research Group, Cambridge Judge Business School, University of Cambridge, Cambridge CB2 1AG, UK; c Integrated Research on Energy, Environment and Society (IREES), Energy and Sustainability Research Institute Groningen (ESRIG), University of Groningen, Groningen 9700 AB, Netherlands; d School of International and Public Affairs, Shanghai Jiao Tong University, Shanghai 200240, China; e Tsinghua-Rio Tinto Joint Research Center for Resources Energy and Sustainable Development, Tsinghua University, Beijing 100084, China

**Keywords:** Electric vertical takeoff and landing aircraft (eVTOL), Flying car, On-road vehicles, Greenhouse gas emissions, Life cycle assessment

## Abstract

Electric Vertical Takeoff and Landing aircraft (eVTOLs), also known as flying cars, are expected to lead a new round of transport transformation owing to their superior travel speed and spatial flexibility. While the benefits in transport efficiency are solid, such transformation is criticized by many for increasing the climate burden. This criticism primarily stems from the significantly higher energy consumption associated with air transport compared to on-road transport. Behind such criticism lies the research gap that the climate impacts of eVTOLs have rarely been examined due to the complexity of eVTOL configuration and operation. This study fills this research gap by establishing a well-to-wheel and cradle-to-gate combined process-based life cycle assessment framework involving flying process modeling, component sizing, and operation simulation modules. Here, we show that a four-seat, 100-km eVTOL emits 0.24 kg CO_2_-eq/passenger-km, which is twice that of a comparable on-road battery electric vehicle. This emissions gap can be narrowed with advanced battery technology and cleaner electricity. Improved battery-specific energy (500 Wh/kg) can reduce eVTOL emissions to 0.15 kg CO_2_-eq/passenger-km, 41.3% higher than battery electric vehicle emissions. When the electricity emission factor decreases to 0.10 kg CO_2_-eq/kWh, eVTOL emissions can be significantly reduced to 0.05 kg CO_2_-eq/passenger-km, which equals battery electric vehicle emissions. Here, we argue that with comprehensive production, regulation, and operation strategies, the upcoming transport transition from vehicles to eVTOLs could be a green one.

## Graphical abstract

**Figure:**
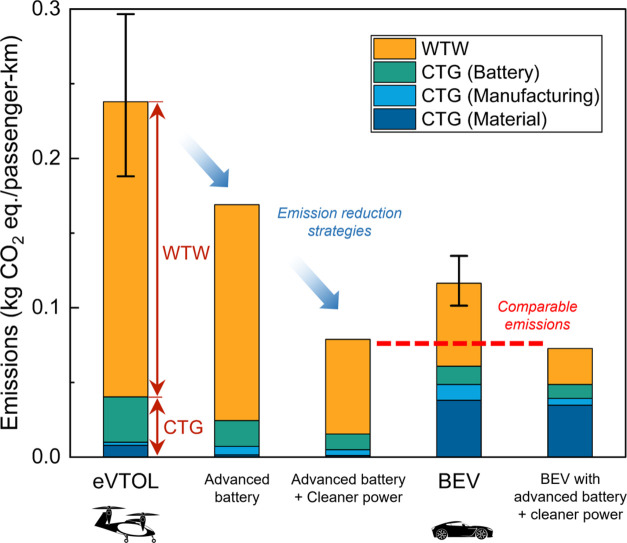

## 1 Introduction

Electric Vertical Takeoff and Landing aircraft (eVTOLs), also known as flying cars, are expected to substantially reshape future transport systems, as they can provide high-speed, point-to-point transport services [1]. For example, an eVTOL flight from San Francisco to San Jose can be completed in only 15 min, compared to 2 h by on-road vehicle [2]. The overall transport system also benefits from the transition to eVTOLs, which could reduce traffic congestion and accidents [3]. This emerging technology has attracted increasing attention from governments, industries, and academia [[4], [5], [6], [7], [8], [9], [10], [11]], with leading companies like EHang, Boeing, and Joby making great progress in improving the technological feasibility of eVTOLs [[12], [13], [14]].

While the benefits in transport efficiency are solid, eVTOLs are often criticized for their Greenhouse Gas (GHG) emissions due to the higher propulsion energy needed compared with on-road vehicles. During eVTOL flights, the propulsion energy needed is 3.5 times that of a comparable on-road electric vehicle, resulting in proportionally higher GHG emissions [15,16]. As a result, eVTOLs are plotted as trading energy consumption and emissions for transport efficiency [17]. However, opportunities exist to mitigate eVTOL emissions, such as adopting cleaner electricity, improved manufacturing processes, and advanced battery technology. These factors can potentially bring eVTOL emissions in line with those of on-road vehicles. Therefore, it is crucial to have a comprehensive evaluation of eVTOLs’ climate impacts.

Several pioneering studies have examined GHG emissions from eVTOLs. Kasliwal et al. [18] compared emissions of eVTOLs and on-road vehicles, finding that levelized emissions of eVTOLs can be lower than those of fossil-fueled vehicles in longer trips. Liu et al. [15,16] calculated emissions from VTOLs with various propulsion systems, showing that eVTOLs emit less GHG than their engine-powered counterparts. While these studies provided a solid foundation for understanding eVTOL emissions, they mostly confined the scope to the Well-to-Wheel (WTW) stages, i.e., emissions from the production, transmission, and use of propulsion electricity. The Cradle-to-Gate (CTG) emissions, including emissions from material production, components production, and eVTOL assembly, also play an essential role in life cycle emissions estimation. Andre et al. [19] and Liberacki et al. [20] calculated eVTOL CTG emissions, finding that takeoff mass and power emission factor significantly impact CTG emissions. However, due to the complexity of eVTOL configuration, they only examined a few materials and lacked a comprehensive, component-level CTG analysis. Filling this research gap is crucial for justifying the eVTOL industry from a climate perspective.

This study bridges this research gap by comparing the life cycle GHG emissions of eVTOL and on-road vehicles, with both WTW and CTG emissions considered. We aim to answer if eVTOLs can achieve co-benefits in GHG abatement in addition to enhancing transport efficiency and how to reach such co-benefits. The Life Cycle Assessment (LCA) approach is the most effective method for this purpose. It provides a comprehensive evaluation of material usage and energy consumption throughout an eVTOL’s life cycle, essential for accurate emission estimation. By accounting for both WTW and CTG stages, this study enables a holistic assessment of eVTOL emissions from both operation and production processes, preventing biased conclusions. Therefore, we established a process-based LCA framework with technology-rich process characterizations involving flying process modeling, key eVTOL component sizing, and operation simulation. Here, we show that although life cycle emissions from an eVTOL are currently higher than those from an on-road vehicle, adopting strategies such as reducing electricity emission factors and improving battery-specific energy can significantly mitigate emissions of eVTOLs, making them an important part of green transport.

## 2 Materials and methods

### 2.1 System boundary

**Fig. 1:**
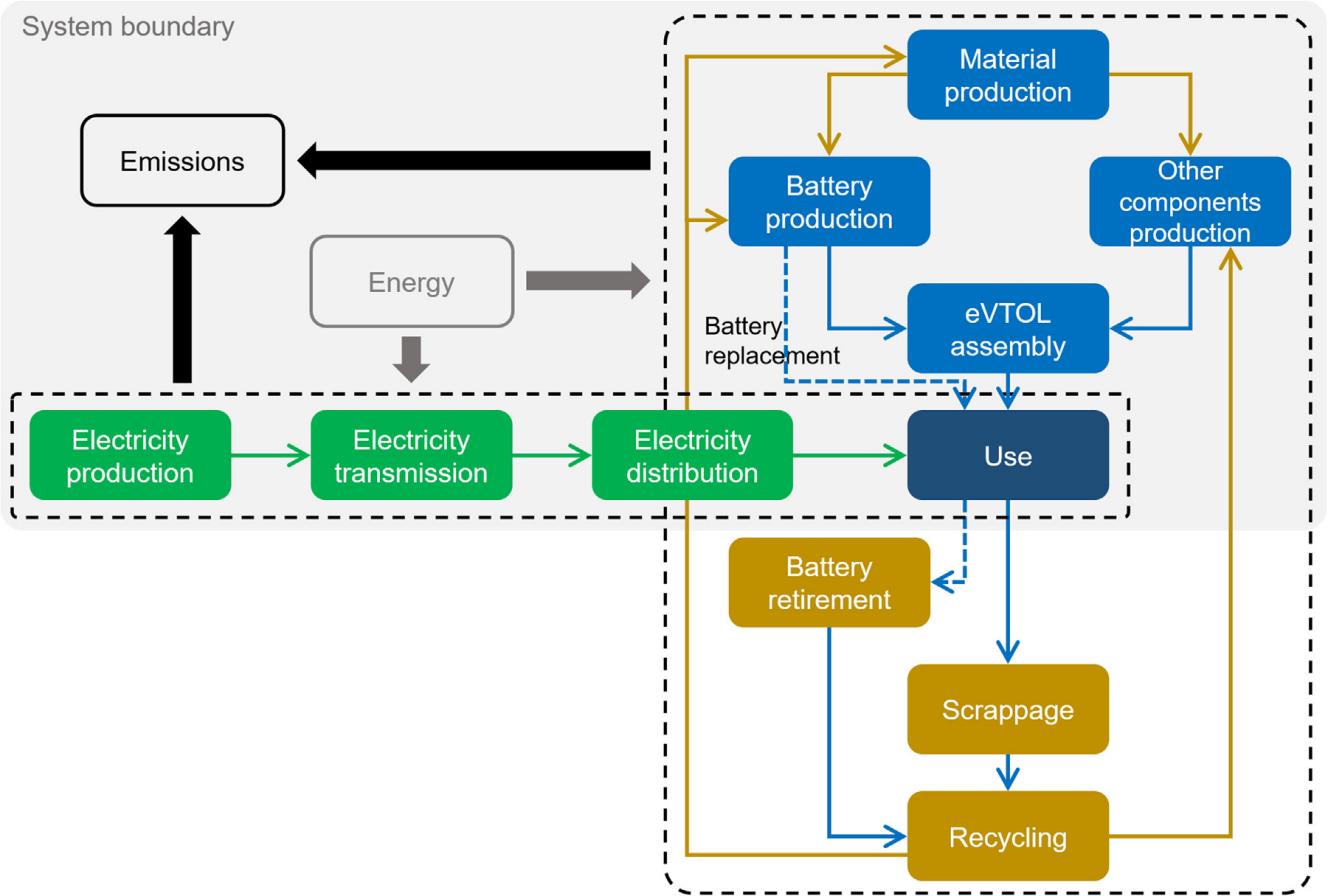
**Research system boundary.** eVTOL, electric vertical takeoff and landing aircraft.

Fig. 1 shows the research system boundary, in which the life cycle emissions of both eVTOLs and on-road vehicles are calculated. Both the fuel cycle and vehicle cycle are considered. The fuel cycle includes electricity production, transmission, distribution, and use. The WTW emissions are the total emissions from these processes. The CTG emissions from the vehicle cycle are accounted for by considering material production, battery production, other components production, and eVTOL assembly processes but excluding scrappage and recycling processes due to the lack of data. The functional unit is defined to be one passenger-km. In the base case, eVTOLs are assumed to be manufactured and used in China, meaning that all energy and material emission factors used in the base case are China-specific data. Results for other countries are also provided. Material emission factors vary with different electricity emission factors, considering the electricity energy used during material production. The climate impact is calculated using the ReCiPe 2016 method, with 100-year Global Warming Potential as the indicator [21].

### 2.2 Flying process model

The flying process is first modeled to obtain key characteristics of eVTOL operation, such as energy consumption. Five sub-phases, namely hovering, climbing, cruising, descending, and landing, are defined within the flying process. While hovering and landing, eVTOLs take off and land vertically. While climbing and descending, eVTOLs fly to or descend from the cruising altitude at a certain angle. While cruising, a horizontal flight is conducted by eVTOLs. The power demand of each sub-phase is calculated based on the kinematic and dynamic characteristics ((1), (2), (3), (4)).

(1) $$P_{hover}\;or\;P_{land}=\frac{m_{TO}g}{\eta_{hover}}\sqrt{\frac{\delta }{2\rho }}$$

(2) $$P_{climb}=\frac{m_{TO}g}{\eta_{climb}}\left(ROC+\frac{ROC}{sin\alpha \times L/D_{climb}\;}\right)$$

(3) $$P_{cruise}=\frac{m_{TO}g}{\eta_{cruise}}\frac{V_{cruise}}{L/D_{cruise}}$$

(4) $$P_{descend}=\frac{m_{TO}g}{\eta_{descend}}\left(-ROD+\frac{ROD}{sin\alpha \times L/D_{descend}\;}\right)$$

where

$P_{hover}$, $P_{land}$, $P_{climb}$, $P_{cruise}$, and $P_{descend}$ are the power demand of an eVTOL while hovering, landing, climbing, cruising, and descending, respectively (kW);
$m_{TO}$ is the maximum takeoff mass (kg);
$\eta_{hover}$, $\eta_{climb}$, $\eta_{cruise}$, and $\eta_{descend}$ are the propulsion system efficiencies while hovering/landing, climbing, cruising, and descending, respectively;
δ is the disk loading (N/m^2^);
ρ is the air density (kg/m^3^);
ROC and ROD are the rate of climb and the rate of descend, respectively (m/s);
$L/D_{climb}$, $L/D_{cruise}$, and $L/D_{descend}$ are the lift-to-drag ratios while climbing, cruising, and descending, respectively;
α is the flight angle (degrees);
g is the acceleration due to gravity (m/s^2^);
$V_{cruise}$ is the cruising speed (m/s).

Total energy consumption during the flight is the sum of each sub-phase’s energy consumption, obtained by multiplying the power demand with the duration of each process (Eq. (5)). The duration of each sub-phase is calculated by dividing the flying distance by the velocity (Eq. (6)), while the hovering and the landing time are set as fixed values. Energy reserve, which is required for eVTOL safety by regulations, is calculated by multiplying the cruising power demand with the additional cruising time (Eq. (7)). The parameters used here are listed in Table S1.

(5) $$E_{c}=\sum P_{i}t_{i}$$

(6) $$t_{i}=\frac{d_{i}}{V_{i}}$$

(7) $$E_{r}=P_{crusie}\times t_{add}$$

where

$E_{c}$ and $E_{r}$ are total energy consumption and energy reserve, respectively (MJ);
$P_{i}$ is the power demand of sub-phase i (kW);
$t_{i}$ and $t_{add}$ are the duration time in sub-phase i and the additional cruising time, respectively (s);
$d_{i}$ is the flying distance in sub-phase i (m);
$V_{i}$ is the velocity in sub-phase i (m/s).

### 2.3 Component sizing model

The takeoff mass of an eVTOL is divided into five parts: airframe mass, powertrain mass, furnishing mass, system & equipment mass, and load (Eq. (8)) [22]. The powertrain mass encompasses battery mass and electric motor mass (Eq. (9)). The battery mass is composed of energy storage battery and auxiliary power unit (Eq. (10)). To meet energy requirements, the energy storage battery must store adequate energy for both energy consumption and reserve (Eq. (11)). Another battery serves as an auxiliary power unit while hovering and landing, due to the high power demand (Eq. (12)). To meet power requirements, electric motors need to provide sufficient power to sustain eVTOL flight, especially during high-power-demand phases like hovering and landing (Eq. (13)). The nacelle in the airframe system protects the battery and its mass is assumed to be proportional to the battery mass (Eq. (14)). Apart from the powertrain mass and nacelle mass, the remaining components account for a fixed share in the takeoff mass (Eq. (15)).

(8) $$m_{TO}=m_{AF}+m_{PT}+m_{FR}+m_{S\&E}+m_{L}$$

(9) $$m_{PT}=m_{B}+m_{EM}$$

(10) $$m_{B}=m_{ESB}+m_{APU}$$

(11) $$m_{ESB}=\frac{E_{c}+E_{r}}{\rho_{Benergy}}$$

(12) $$m_{APU}=\frac{P_{hover}-m_{ESB}\times \rho_{Bpower}}{\rho_{APUpower}}$$

(13) $$m_{EM}=\frac{P_{hover}}{\rho_{EMpower}}+m_{EM_{0}}$$

(14) $$m_{NC}=R_{NC-B}\times m_{B}$$

(15) $$m_{TO}\times R_{AOC-TO}=m_{AF}+m_{FR}+m_{S\&E}-m_{NC}$$

where

$m_{AF}$, $m_{PT}$, $m_{FR}$, $m_{S\&E}$, and $m_{L}$ are airframe mass, powertrain mass, furnishing mass, system & equipment mass, and load (kg);
$m_{B}$, $m_{ESB}$, $m_{APU}$, and $m_{EM}$ are the battery mass, energy storage battery mass, auxiliary power unit battery mass, and electric motor mass (kg);
$\rho_{Benergy}$ is battery specific energy (Wh/kg);
$\rho_{APUpower}$ and $\rho_{Bpower}$ are the power density of auxiliary power unit and energy storage battery (kW/kg);
$\rho_{EMpower}$ is electric motor power density (kW/kg);
$m_{EM_{0}}$ is the initial mass of electric motors (kg);
$m_{NC}$ is the nacelle mass (kg);
$R_{NC-B}$ is the ratio of nacelle mass to the total battery mass;
$R_{AOC-TO}$ is the ratio of remaining components mass to the takeoff mass.

The airframe is divided into multiple components, including the nacelle, empennage, landing gear, rotor, wing, and fuselage. The system & equipment encompass multiple devices for eVTOL operation, including hydraulics, instruments, air conditioning, flight control device, load handling device, avionics, electrics, and power electronics. The mass of these components, excluding those already accounted for, maintains a predetermined proportional relationship to their total mass (Eq. (16)). The proportional relationship is derived from the U.S. Army Aero Flight Dynamics Directorate (AFDD) model [22,23]. The parameters used here are listed in Table S2.

(16) $$m_{j}=R_{j}\times \;\left(m_{AF}+m_{FR}+m_{S\&E}-m_{NC}\right)$$

where

$m_{j}$ is the mass of component j, including empennage, landing gear, wing, fuselage, rotor, hydraulics, instrument, air conditioning, flight control, load handling, avionics, electrics, power electronics, and furnishing (kg);
$R_{j}$ is the ratio of component mass j to their total mass.

### 2.4 Operation simulation model

In this research, a fixed yearly operation time is assumed for eVTOLs. To simplify calculations, an eVTOL is assumed to travel the same distance (100 km) in each mission. By dividing the yearly operation time by the mission time, the number of missions per year is calculated and the result is rounded to the nearest integer (Eq. (17)). The total passenger-km travelled can be further calculated based on the number of missions per year, flying range, eVTOL life, number of seats, and the load factor (or occupancy rate) (Eq. (18)). A load factor of 50% is assumed. The parameters used here are listed in Table S3.

(17) $$N_{mission}=\left[\frac{t_{YO}}{t_{mission}}\right]$$

(18) $$PKT=VL\times N_{mission}\times R\times N_{seat}\times LF$$

where

$N_{mission}$ is the number of missions per year, which is rounded to the nearest integer;
$t_{YO}$ and $t_{mission}$ are the yearly operation time and mission time (h);
PKT is the passenger-km traveled (passenger-km);
R is the range of eVTOLs (km);
VL is the eVTOL life (year);
$N_{seat}$ is the number of seats;
LF is the load factor.

### 2.5 Emissions calculation model

Based on flying process and operation simulation models, the total WTW emissions from one eVTOL are obtained by multiplying the total energy consumption with the electricity emission factor (Eq. (19)). The levelized WTW emissions are then accounted for by dividing total WTW emissions by total passenger-km travelled (Eq. (20)).

(19) $$E_{WTW}=VL\times N_{mission}\times E_{c}\times EF_{e}$$

(20) $$E_{LWTW}=\frac{E_{WTW}}{PKT}$$

where

$E_{WTW}$ and $E_{LWTW}$ are the total and levelized WTW emissions (t CO_2_-eq and kg CO_2_-eq/passenger-km);
$EF_{e}$ is the electricity emission factor (kg CO_2_-eq/kWh).

The CTG emissions are the sum of emissions from material production, battery production, and other components & eVTOL production (Eq. (21)). The Bill of Materials (BoM) of each component is obtained based on electric aircraft life cycle inventory dataset [24,25], which provides detailed data on materials used and their mass in each component. By multiplying the mass of each material with its emission factor accordingly, material production emissions can be obtained (Eq. (22)). Battery production emissions are calculated based on the energy consumption for battery production, the associated emission factors, and the frequency of battery replacements (Eq. (23)). The battery replacement frequency calculation is simplified by assuming that eVTOLs travel 100 km, the designed range, in each mission, meaning the battery undergoes a charge-discharge cycle per mission. By dividing the battery cycle life by the number of missions per day, we determine when the battery reaches the State of Health (SOH) threshold. Calculating emissions from other components & eVTOL production uses the same approach as above (Eq. (24)). Using the total passenger-km travelled data, levelized CTG emissions are further calculated (Eq. (25)). The parameters used here are listed in Tables S4-S6.

(21) $$E_{CTG}=E_{MT}+E_{BP}+E_{CP\&VA}$$

(22) $$E_{MT}=\sum_{j}\sum_{k_{j}}\left(R_{k_{j}-j}\times m_{j}\times EF_{k}\right)$$

(23) $$E_{BP}=\left(1+N_{BR}\right)\;\times \sum_{l}\left(E_{c_{l_{B}}}\times m_{B}\times EF_{l}\right)$$

(24) $$E_{CP\&VA}=\sum_{j}\sum_{l_{j}}\left(E_{c_{l_{j}}}\times m_{j}\times EF_{l}\right)$$

(25) $$E_{LCTG}=\frac{E_{CTG}}{PKT}$$

where

$E_{CTG}$ and $E_{LCTG}$ are the total and levelized CTG emissions (t CO_2_-eq and kg CO_2_-eq/passenger-km);
$E_{MT}$, $E_{BP}$, and $E_{CP\&VA}$ are material production, battery production, and other components production & eVTOL assembly emissions (kg CO_2_-eq);
$R_{k_{j}-j}$ is the share of material k in component j;
$EF_{k}$ is the emission factor of material k (kg CO_2_-eq/kg);
$N_{BR}$ is the battery replacement frequency;
$E_{c_{l_{j}}}$ is the consumption of energy l while producing component j (kWh/kg);
$EF_{l}$ is the emission factor of energy l (kg CO_2_-eq/kWh).

WTW and CTG emissions from on-road Battery Electric Vehicles (BEVs), Internal Combustion Engine Vehicles (ICEVs), and Plug-in Hybrid Electric Vehicles (PHEVs) are also calculated for comparison, using the similar LCA framework as eVTOLs. The mass of each component, BoM, and energy consumption for battery and vehicle production are derived from the GREET model [26]. The average values for vehicle specifications, such as vehicle weight, energy consumption rate, and battery capacity, are used for calculation in this study and they are sourced from GREET [26], EV database [27], and the Ministry of Industry and Information Technology of China [28]. Vehicles are categorized into five segments (mini, small, compact, mid-size, and large) based on their wheelbase. Beyond the calculation based on average values, emissions from BEVs in various segments are also provided. Emission factors for energy and materials of different on-road vehicles are the same. All emissions are calculated using the ReCiPe 2016 method.

### 2.6 Sensitivity analysis

Uncertainties exist in several parameters. To evaluate the impact of these uncertainties, a sensitivity analysis on key parameters is conducted. Given that key technologies like batteries can undergo significant improvement, calculating eVTOL emissions using projected future values of these parameters is crucial to understanding potential advancements. These two analyses follow the same approach – changes of parameters are input into the corresponding models and the resulting emissions are derived. For example, improved battery-specific energy is input into the component sizing model, which outputs updated energy consumption and mass distribution. With these data, eVTOL emissions can be updated accordingly.

## 3 Results

### 3.1 Life cycle GHG emissions from eVTOLs

A reference eVTOL configuration is employed for base-case emissions calculation. This configuration, featuring seats for four (one pilot and three passengers) and 100-km range capacity, aligns with mainstream prototypes for potential air taxi deployment [2,29]. The flying process is subsequently modeled to reflect the energy and power demands of eVTOLs. The airframe, battery, electric motor, and takeoff mass are sized accordingly to fulfill load, energy, and power requirements. A life cycle inventory, including materials and energy inputs for eVTOL production and operation, is then established. The climate impacts of eVTOLs are estimated using Global Warming Potential (GWP) as the indicator. Both CTG and WTW emissions are accounted.for Energy and material emission factors used in the base case are China-specific data (e.g., 0.57 kg CO_2_-eq/kWh for electricity emission factor), while the results for other countries are analyzed in subsequent sections. The emissions, measured by kilogram CO_2_ equivalent per passenger per kilometer travelled (kg CO_2_-eq/passenger-km), are then levelized by passenger-km travelled (PKT) for comparison with on-road vehicles. A load factor of 50% is assumed for PKT calculation in this study. Notably, the required travelling distance for achieving the same origin-to-destination task for eVTOLs is typically shorter than on-road vehicles because eVTOLs travel straightly while on-road vehicles travel more due to road network complexity. The impact of such difference is discussed subsequently.

**Fig. 2:**
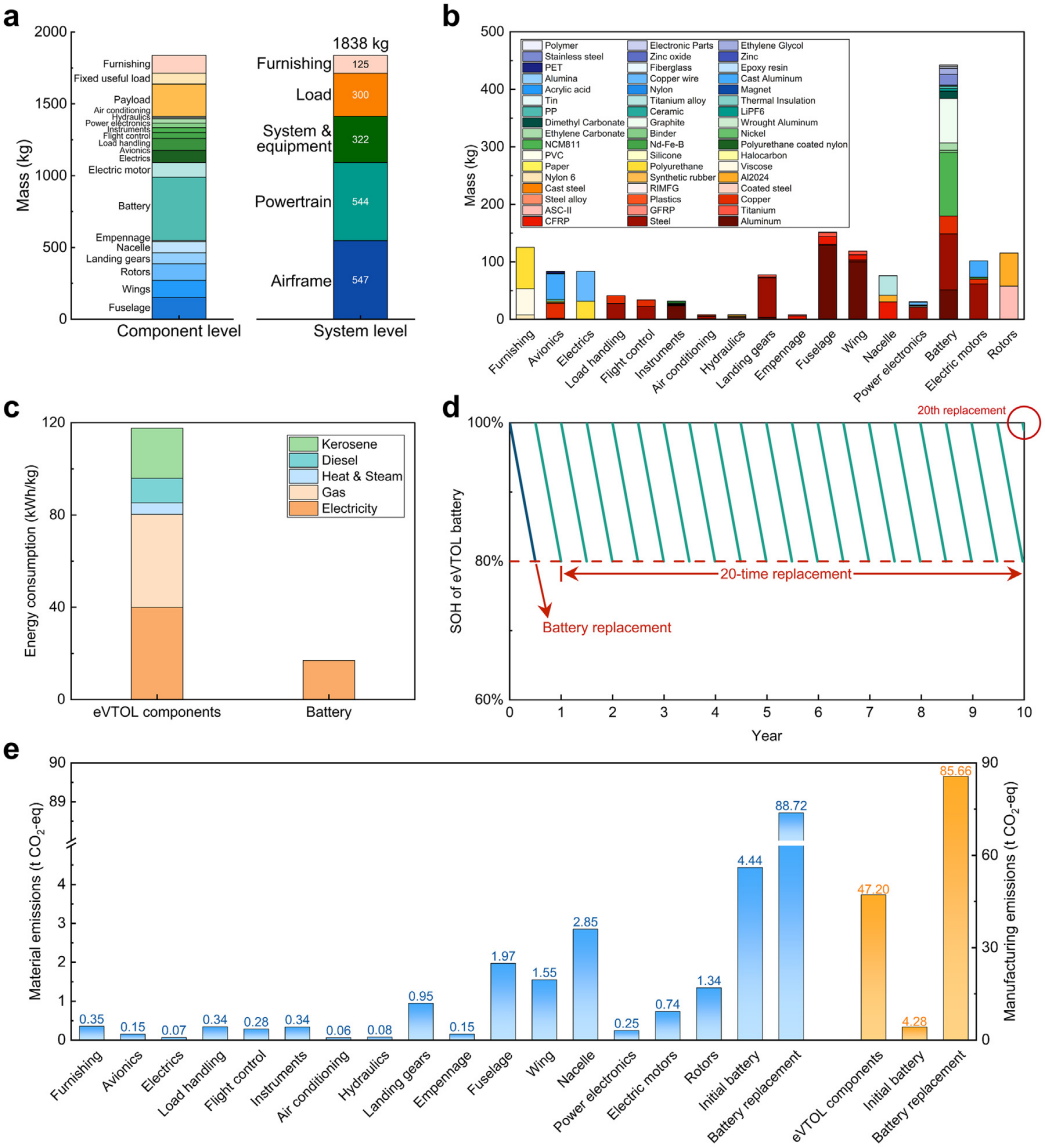
**CTG emissions from eVTOLs.** (a) The system-level and component-level mass distribution of a reference eVTOL. (b) Material mass distribution of each component. (c) Energy consumption during components and eVTOL production. (d) Battery replacement frequency over eVTOL’s lifetime, with the 20th replacement highlighted. (e) Material (left axis) and manufacturing (right axis) emissions from one eVTOL. Bill of materials data are provided in Fig. S1 and Table S4, with material mass distribution results under different battery-specific energy shown in Figs. S2-S6. eVTOL, electric vertical takeoff and landing aircraft; CFRP, carbon fiber reinforced plastic; GFRP, glass fiber reinforced plastic; PP, polypropylene; PET, polyethylene terephthalate; RIMFG, resin impregnated micro fiber glass; NCM, nickel cobalt manganese; SOH, state of health; CTG, cradle-to-gate.

The modeling of the reference configuration leads to an eVTOL with an 88 kWh battery capacity, 366 kW propulsion power, 52.0 kWh/100 km energy consumption rate, and 1838 kg takeoff mass (Fig. 2a). Five systems are defined within one eVTOL, namely airframe, powertrain, system & equipment, load, and furnishing. Among these, the airframe stands as the largest system, sized at 547 kg. The fuselage and wings are the two main components in the airframe, weighing 152 and 119 kg, respectively. The powertrain mass (544 kg) is another important contributor to takeoff mass, accounting for 29.6% of the total mass. Notably, a Nickel Cobalt Manganese (NCM) battery with 250-Wh/kg specific energy and a life of 2000 cycles is assumed for base-case estimation. This leads to a battery mass of 442 kg. Due to the high power needed during flight, a high electric motor mass (102 kg) is required. The system & equipment category encompasses multiple devices essential for eVTOL operation, with avionics and electrics weighing 84 kg each, while the other components weigh < 45 kg.

Fig. 2b shows the breakdown of eVTOL components into materials. Steel and aluminum are two major materials used in eVTOLs, weighing 312 and 309 kg per eVTOL, respectively. Different alloys, such as ASC-II, are also utilized in eVTOLs to ensure high strength. Coupled with emission factors of the materials (Table S6), emissions associated with material production can be accounted for (Fig. 2e). Like on-road transport, batteries emerge as a major contributor to material emissions in eVTOLs due to the high mass and utilization of high-emission-intensity materials, e.g., NCM cathode. For one eVTOL, total emissions from battery material production are 4.4 t CO_2_-eq, higher than those from other components. The production of nacelle materials, primarily titanium, emits 2.9 t CO_2_-eq. Total emissions from fuselage and wings material production are 2.0 and 1.6 t CO_2_-eq, respectively, both relatively high due to the aluminum composition. Rotors, which are mainly made of aluminum alloy, yield emissions of 1.3 t CO_2_-eq in the material production process. Electric motors, despite the high mass, impose a marginal impact on material emissions, with emissions of 0.7 t CO_2_-eq.

Fig. 2c shows the energy consumed in eVTOL manufacturing processes, with associated emissions illustrated in Fig. 2e. Producing 1 kg of eVTOL requires 40.0 kWh electricity, 40.2 kWh natural gas, 5.0 kWh heat & steam, 10.7 kWh diesel, and 21.7 kWh kerosene energy [24,30]. This implies a total of 47.2 t GHG emissions (Fig. 2e). The energy consumption for battery production is illustrated separately due to its significant impact. The electricity required for producing 1 kg of battery is 17.0 kWh [26], leading to 4.3 t GHG emissions in total.

Batteries in eVTOLs have to be replaced frequently due to the high-frequency eVTOL operation and strict safety requirements on aircraft. As shown in Fig. 2d, using 80% SOH as the limit, the calculation results show that an eVTOL battery with a 2000-cycle life reaches the SOH threshold within about half a year, which can be characterized as biannual battery replacements for the eVTOL. Considering eVTOLs’ 10-year lifespan, the total battery replacement frequency is expected to reach 20 times. Material and production emissions from battery replacement are estimated at 88.7 and 85.7 t CO_2_-eq over eVTOLs’ lifetime, significantly higher than emissions from other components. This highlights the key role of battery technology in eVTOL emissions.

The WTW emissions are derived by multiplying energy consumption with the associated electricity emission factor (Table S5). The 52.0 kWh/100 km energy consumption rate and 4000,000 km lifetime mileage of the reference eVTOL leads to 1190 t CO_2_-eq WTW emissions in total.

**Fig. 3:**
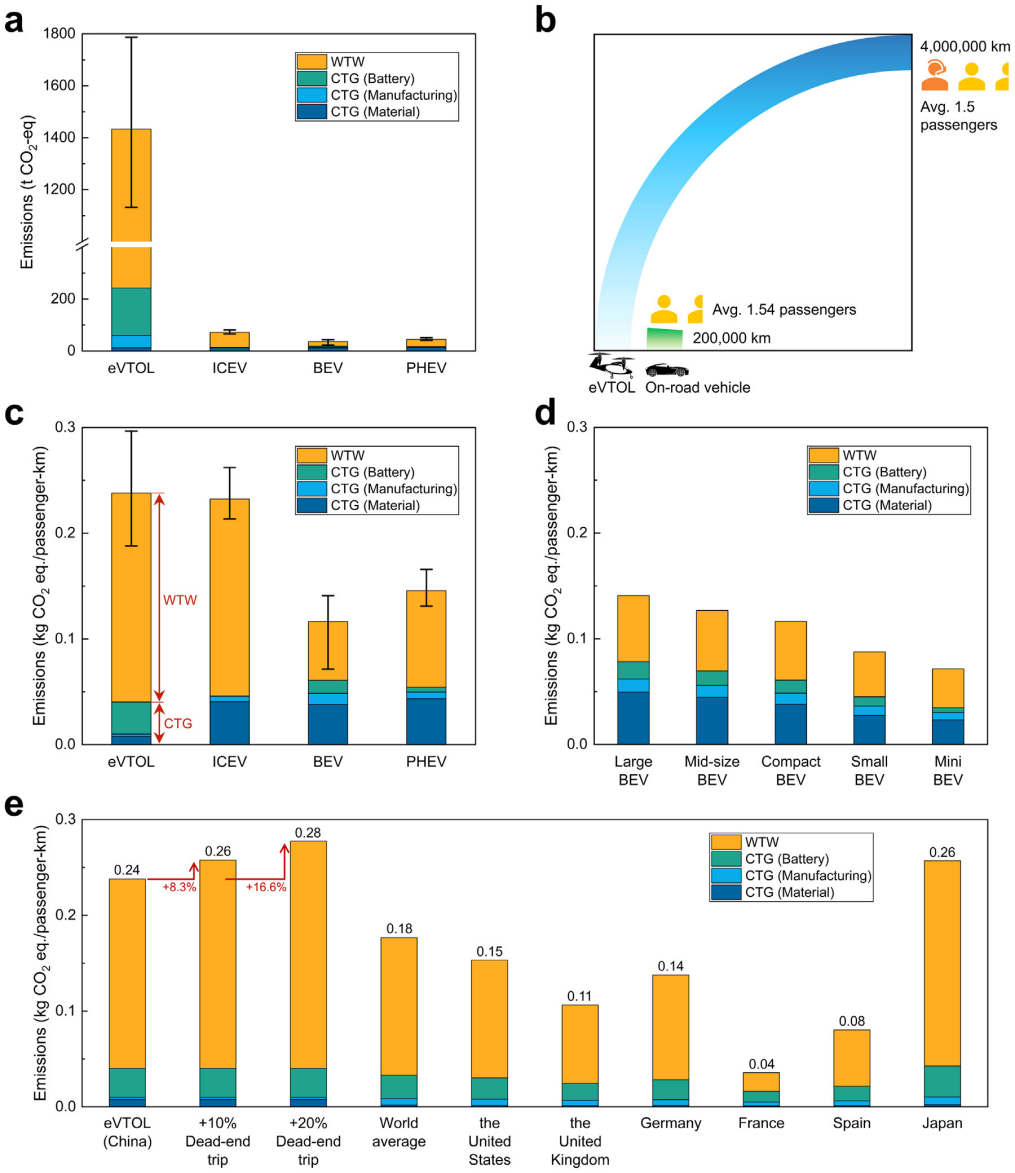
**Life cycle emissions from eVTOLs and on-road vehicles.** (a) Total emissions from one eVTOL, one ICEV, one BEV, and one PHEV. (b) Mileage traveled during the lifetime of each eVTOL and each on-road vehicle, with the average number of passengers carried per trip. (c) Levelized emissions. (d) Emissions from BEVs across different segments (mini, small, compact, mid-size, and large). (e) Emissions with different dead-end trip assumptions and emissions in various countries. The error bars represent the variability of life cycle emissions due to different eVTOL and vehicle sizes. eVTOL, electric vertical takeoff and landing aircraft; ICEV, internal combustion engine vehicle; BEV, battery electric vehicle; PHEV, plug-in hybrid electric vehicle; CTG, cradle-to-gate; WTW, well-to-wheel.

Combining both CTG and WTW emissions, Fig. 3 shows the life cycle emissions per eVTOL. The life cycle emissions from on-road vehicles, estimated using a similar LCA framework as eVTOLs, are provided for comparison. Average values of vehicle specifications, such as battery capacity, sourced from [[26], [27], [28]], are used for calculation. Battery emissions (all battery-related emissions, including battery material and manufacturing emissions from both initial battery and battery replacement) are individually illustrated, while material and manufacturing emissions of other components are shown separately (Fig. 3a). Total life cycle emissions from the reference eVTOL reach 1433 t CO_2_-eq. WTW emissions are prominent with a share of 83.7%. Battery emissions account for 12.2% of total emissions, attributed to high battery replacement emissions. Emissions from the material and manufacturing of other components account for 0.9% and 3.3% of total emissions, respectively, indicating their marginal impacts.

Total emissions per eVTOL significantly surpass those from different on-road vehicles. Emissions from one ICEV, one BEV, and one PHEV are 72, 36, and 45 t CO_2_-eq, respectively, about one-twentieth, one-fortieth, and one-thirtieth of eVTOL emissions. The reasons behind such a substantial gap are differences in their operation characteristics (Fig. 3b). With a 10-year life and 2000-hour yearly operation time, an eVTOL is projected to cover > 4000,000 km during its lifetime, whereas an on-road vehicle is typically driven for 200,000 km.

When looking at the life cycle emissions from a levelized perspective, i.e., per-passenger-km emissions, the emissions gap between eVTOL and on-road vehicles narrows substantially (Fig. 3c). The levelized life cycle emissions per eVTOL are 0.24 kg CO_2_-eq/passenger-km, 2.4%, 104.4%, and 63.3% higher than those from an ICEV, a BEV, and a PHEV. From such a perspective, eVTOLs have already reached a level comparable to that of ICEVs. The gap between eVTOLs and BEVs and PHEVs, however, is still apparent. When compared to high-capacity transportation modes such as railways, the levelized emissions of eVTOLs are estimated to be 2–10 times higher [20], highlighting the emission reduction advantage of high-capacity transport.

A sensitivity analysis on vehicle sizes is provided. Taking BEVs for example (Fig. 3d), large-segment BEVs, featuring larger batteries and higher vehicle weight, emit 0.14 kg CO_2_-eq/passenger-km – only 41.7% lower than eVTOL emissions. In contrast, mini-segment BEVs with small batteries emit only 0.07 kg CO_2_-eq/passenger-km – less than one-third of eVTOL emissions. Dead-end trips, which occur when eVTOLs need to pick up passengers at different locations, can increase emissions. With a 10% dead-end trip share, eVTOL emissions rise to 0.26 kg CO_2_-eq/passenger-km, 8.3% higher than the base-case result. This highlights the need for efficient operation to reduce emissions associated with dead-end trips. China-specific data are used for base-case estimation, while results for other countries are shown in Fig. 3e. With the global average power emission factor, eVTOLs emit 0.18 kg CO_2_-eq/passenger-km – 25.7% lower than base-case emissions. In the U.S., eVTOL emissions are 0.15 kg CO_2_-eq/passenger-km, while in France, emissions can drop to 0.04 kg CO_2_-eq/passenger-km.

### 3.2 Potential for eVTOL GHG reduction

**Fig. 4:**
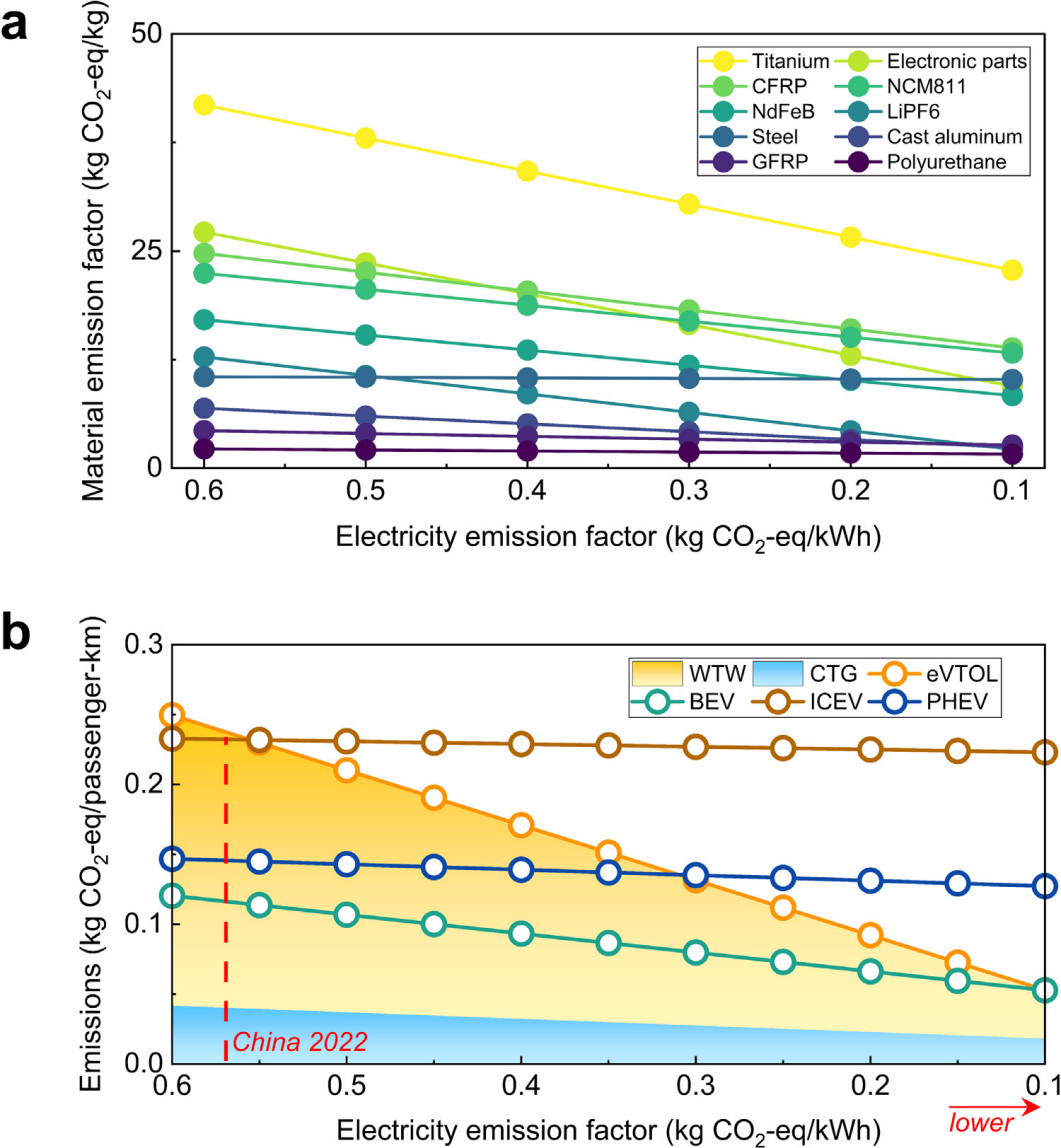
**Impact of reducing electricity emission factor on emissions reduction.** (a) Changes in material emission factors when electricity emission factor decreases, using ten materials as examples. (b) Emissions with reduced electricity emission factors. CTG emissions with varying electricity emission factors, battery-specific energy, and cycle life assumptions are provided in Figures S7-S16 and S27-S34, with corresponding life cycle emissions provided in Figures S17-S26 and S35-S50. eVTOL, electric vertical takeoff and landing aircraft; ICEV, internal combustion engine vehicle; BEV, battery electric vehicle; PHEV, plug-in hybrid electric vehicle; CTG, cradle-to-gate; WTW, well-to-wheel; CFRP, carbon fiber reinforced plastic; GFRP, glass fiber reinforced plastic; NCM, nickel cobalt manganese.

**Fig. 5:**
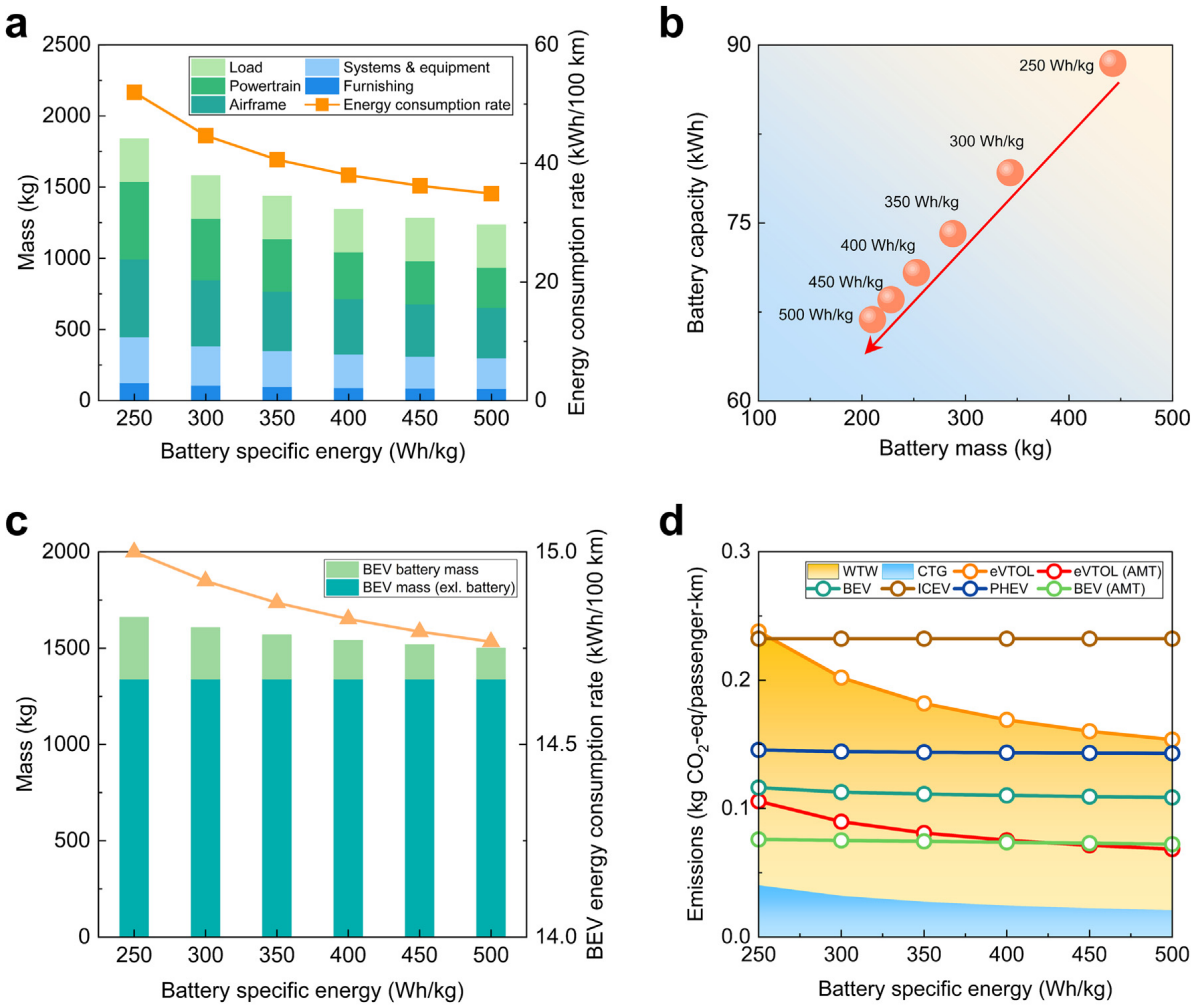
**Impact of improving battery specific energy on emissions reduction.** (a) Changes in eVTOL takeoff mass and energy consumption rate with improved specific energy. (b) Changes in eVTOL battery mass and capacity with improved specific energy. (c) Changes in BEV weight and energy consumption rate with improved specific energy. (d) Emissions with improved specific energy. The relationship between BEV weight and energy consumption rate is shown in Figure S51. AMT, all measure taken scenario; eVTOL, electric vertical takeoff and landing aircraft; ICEV, internal combustion engine vehicle; BEV, battery electric vehicle; PHEV, plug-in hybrid electric vehicle; CTG, cradle-to-gate; WTW, well-to-wheel.

**Fig. 6:**
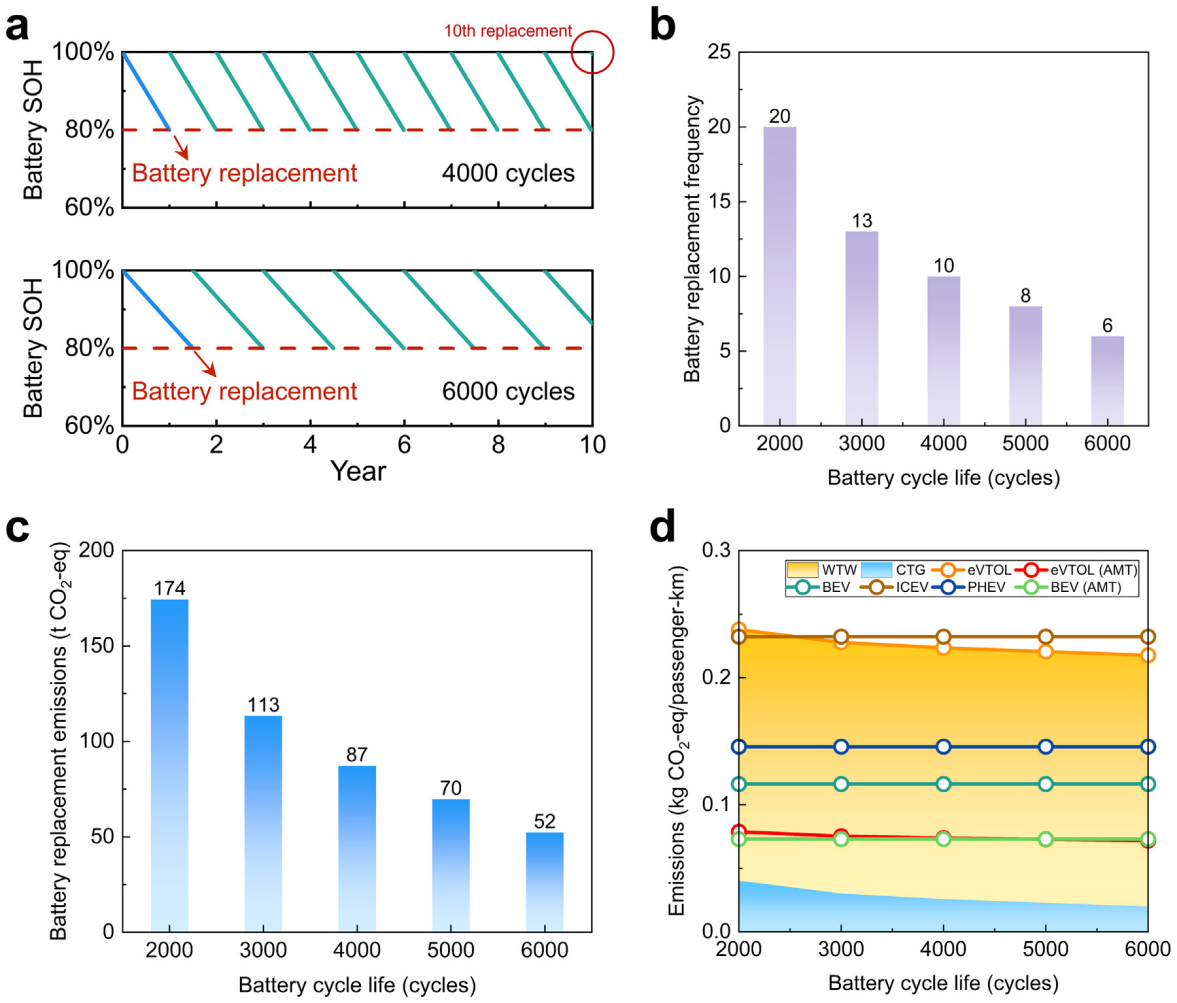
**Impact of improving battery life on emissions reduction.** (a) Changes in battery SOH with 4000-cycle and 6000-cycle battery life. (b) Battery replacement frequencies with improved cycle life. (c) Battery replacement emissions with improved cycle life. (d) Emissions with improved cycle life. eVTOL, electric vertical takeoff and landing aircraft; ICEV, internal combustion engine vehicle; BEV, battery electric vehicle; PHEV, plug-in hybrid electric vehicle; CTG, cradle-to-gate; WTW, well-to-wheel; SOH, state of health; AMT, all measure taken scenario.

While eVTOLs show higher GHG emissions than on-road BEVs in the base case, numerous factors can potentially contribute to mitigating eVTOL emissions. Fig. 4, Fig. 5, Fig. 6 show the representative strategies and their potential impacts.

The first and most important strategy focuses on reducing the electricity emission factor by adopting renewable power. Fig. 4b shows the emission reductions for both eVTOL and on-road vehicles (BEV, PHEV, and ICEV), with electricity emission factor decreasing. While life cycle emissions per eVTOL are 2.4% higher than those from an ICEV under the base case, this gap can be filled by decreasing the electricity emission factor to 0.55 kg CO_2_-eq/kWh. With a lower emission factor of 0.30 kg CO_2_-eq/kWh, eVTOL emissions could decrease to the same level of PHEVs. A further decrease to 0.10 kg CO_2_-eq/kWh emission factor, close to the current level in Denmark [31], enables eVTOL to have the same emissions as a BEV. Certainly, clean electricity can also reduce emissions from BEVs and PHEVs. But the reduction potential for eVTOLs is still larger than that for on-road vehicles, because eVTOLs consume significantly more electricity and can get more benefits from the electricity transition.

The second strategy focuses on battery-specific energy improvement, which leads to lower battery mass, and the consequent lower takeoff mass and energy consumption rate, thereby reducing CTG and WTW emissions. The impact of improved specific energy on mitigating emissions from BEVs and PHEVs is also considered. Higher specific energy can reduce both battery and vehicle weight, leading to lower vehicle energy consumption rates, and ultimately decreasing both WTW and CTG emissions. When battery specific energy increases to 300 Wh/kg, the required battery shrinks to 79 kWh and 343 kg, 10.2% and 22.4% lower than the base case (Fig. 5b). Both the takeoff mass (1581 kg) and energy consumption rate (44.7 kWh/100 km) are reduced by 14.0% (Fig. 5a). Life cycle emissions per eVTOL decrease to 0.20 kg CO_2_-eq/passenger-km, being 13.0% lower but 79.1% higher than those from an ICEV and a BEV, respectively (Fig. 5d). With the application of Solid-State Batteries (SSBs), battery-specific energy can be increased to 500 Wh/kg [32], resulting in 25.0% and 52.4% reductions in battery capacity (66 kWh) and mass (210 kg), respectively. The takeoff mass is decreased to 1235 kg, with a 32.8% lower energy consumption rate (34.9 kWh/100 km). In this context, the emissions gap between one eVTOL (0.15 kg CO_2_-eq/passenger-km) and one BEV can be further narrowed to 41.3%.

Due to the smaller battery capacities and lower energy consumption rates of on-road vehicles, the benefits of improving battery-specific energy are less significant compared to eVTOLs. For BEVs, increasing the specific energy to 300 Wh/kg reduces the battery weight and energy consumption rate by 16.7% and 0.5%, respectively (Fig. 5c), resulting in a 3.1% decrease in emissions (Fig. 5d). A further increase to 500 Wh/kg reduces BEV emissions by 6.6%. For PHEVs that have even smaller batteries than BEVs, the impact is more marginal. Enhancing battery specific energy to 300 and 500 Wh/kg reduces PHEV emissions by only 0.9% and 1.8% (Fig. 5d).

The third strategy focuses on improving battery cycle life with the aim of cutting down the battery replacement frequency. In the reference eVTOL configuration, a 2000-cycle-life battery reaches the SOH limit within half a year, necessitating 20 times replacements over the eVTOL’s 10-year lifetime. When the battery life is extended to 3000 cycles, the battery replacement frequency decreases to 13 times (Fig. 6b), leading to reduced battery replacement emissions of 113 t CO_2_-eq (Fig. 6c). Consequently, with lower CTG emissions (0.03 kg CO_2_-eq/passenger-km), eVTOL emissions are reduced to 0.23 kg CO_2_-eq/passenger-km, 1.9% lower but 95.7% higher than those from an ICEV and a BEV, respectively (Fig. 6d). With rapid technology development, if the battery life is increased to 6000 cycles, then the eVTOL battery can maintain sufficient SOH for >1 year, resulting in 6-time battery replacement and 52 t CO_2_-eq battery replacement emissions in total. Life cycle emissions per eVTOL are thereby reduced to 0.22 kg CO_2_-eq/passenger-km, reaching a level comparable to an ICEV. An apparent gap between eVTOL and BEV emissions, however, still exists.

With all the above-mentioned measures taken simultaneously (AMT scenario), the life cycle GHG emissions per eVTOL can be significantly mitigated, achieving much greater reductions. For example, with electricity emission factor reduced to 0.25 kg CO_2_-eq/kWh (current level in Italy) [31], battery specific energy increased to 400 Wh/kg, and battery life increased to 3000 cycles (the latter two can potentially be realized with SSBs or advanced NCM batteries) [10,[33], [34], [35]], which are challenging yet achievable targets in their respective areas in the medium term, eVTOL emissions are reduced to 0.075 kg CO_2_-eq/passenger-km, comparable to those from a BEV. Notably, though measures discussed above, particularly reducing power emission factors, can significantly mitigate eVTOL emissions, achieving these targets on emission factors and battery technologies is challenging. A more detailed discussion on their feasibility is provided in the subsequent section.

**Fig. 7:**
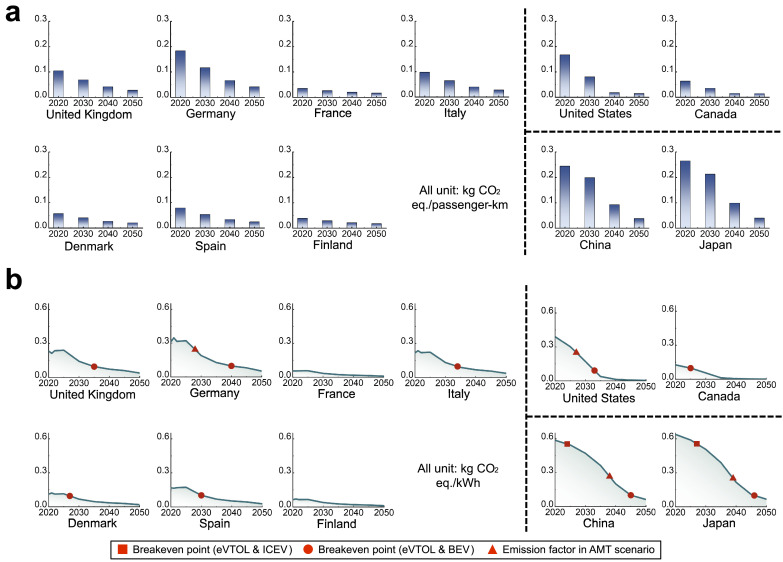
**Life cycle emissions from an eVTOL in different countries.** (a) Life cycle emissions per eVTOL in different countries. (b) Electricity emission factors of different countries, with the breakeven points being depicted. The right mark means that the country has already achieved the breakeven point by 2020. Projections on electricity emission factors are obtained based on data from European Environment Agency [31], the U.K. government [36], Enerdata [37], the Ministry of Ecology and Environment of the People’s Republic of China [38], and the International Energy Agency [39]. eVTOL, electric vertical takeoff and landing aircraft; ICEV, internal combustion engine vehicle; BEV, battery electric vehicle; AMT, all measures taken scenario.

The regional heterogeneity of electricity emission factors leads to significant variability in eVTOL emissions, meaning distinct eVTOL GHG implications in different countries (Fig. 7). Emission reductions for both eVTOL and on-road vehicles due to emission factor decrease are considered. eVTOL emissions are already lower than ICEV emissions in most listed countries. The breakeven between eVTOL and BEV emissions is a near-term prospect for many countries. For Canada, for example, with a projected 0.06 kg CO_2_-eq/kWh emission factor by 2030, eVTOL emissions are only 0.035 kg CO_2_-eq/passenger-km, 26.0% lower than BEV emissions. In France and Sweden, where current emission factors already reside below 0.10 kg CO_2_-eq/kWh, eVTOLs have already become greener than BEVs, indicating that large-scale eVTOL deployment can be accelerated in such countries from the climate perspective. Even in countries with higher power emission factors, such as China, the breakeven between eVTOL and BEV emissions can be expected by no later than 2040.

**Fig. 8:**
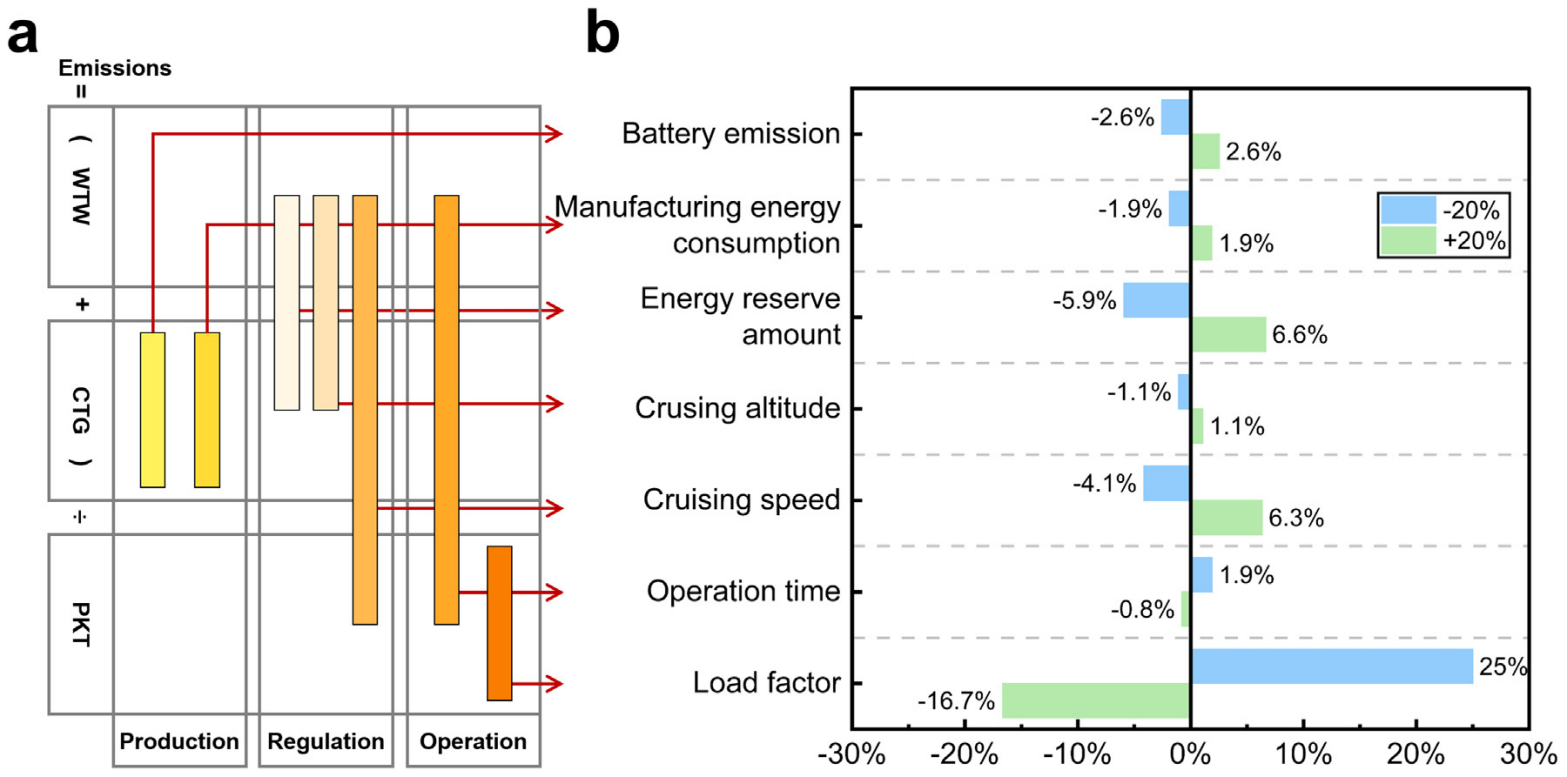
**Influencing factor analysis of eVTOL emissions.** (a) Categories of influencing factors, in which the emissions calculation is divided into three main processes, WTW, CTG, and PKT calculation (vertical axis), with three factor categories (horizontal axis), production-related, regulation-related, and operation-related. (b) Impact analysis of seven influencing factors. eVTOL, electric vertical takeoff and landing aircraft; WTW, well-to-wheel; CTG, cradle-to-gate; PKT, passenger km traveled.

Aside from the measures that lie at the heart of transport electrification as discussed above, several other production-, regulation-, and operation-related factors can also contribute to reducing eVTOL emissions. Impacts from seven representative factors are simulated and summarized in Fig. 8.

From the production perspective, producing advanced batteries (e.g., SSBs) with new materials and techniques potentially emits more GHG. If battery emissions are 20% higher, eVTOL emissions will increase by 2.6%. Besides, eVTOL manufacturing is still in the early stage of development, implying considerable potential for energy consumption optimization. With manufacturing energy consumption reduced by 20%, eVTOL emissions can be reduced by 1.9%.

Regarding regulation-related factors, current regulations impose strict limitations on the energy reserve amount, which must be adequate for a 15-min additional eVTOL flight [18]. However, there is potential for relaxation with significant improvements in eVTOL safety in the future. A 20% reduction in energy reserve amount requirements will decrease the battery mass by 8.7%, resulting in a 5.9% reduction in eVTOL emissions. Regulations mandate sufficient cruising altitude to ensure safe eVTOL flights. But in scenarios with no high-rise buildings, such as rural trips, eVTOLs can operate at lower altitudes, which helps reduce energy consumption during the climbing process. It is estimated that eVTOL emissions can be reduced by 1.1% when the cruising altitude decreases by 20%. A lower cruising speed, which is mainly required in intra-city missions, decreases the power demand, thereby reducing the energy consumption. Although the PKT is also lower due to fewer missions conducted, an overall life cycle emissions reduction of 4.1% can be achieved with a 20% reduction in cruising speed.

Operation-wise, increasing operation time boosts PKT and thus lowers levelized GHG emissions. While this measure also accelerates battery degradation, which results in a higher battery replacement frequency, a 0.8% GHG reduction can be expected when the operation time is increased by 20%. Improving the load factor can directly increase the PKT, significantly reducing levelized emissions. A 20% higher load factor increases PKT by 20%, resulting in a 16.7% decrease in eVTOL emissions, while a 20% lower load factor leads to 25.0% higher emissions.

## 4 Discussion

Our study reveals a picture of future transportation in which the transformation from road transport to air transport can bring either a climate burden or benefit. A wide range of factors, particularly electricity emission factor, battery technology, and operation strategy, influence the climate impacts of such transition. We quantitatively estimate the GHG impacts of these factors and demonstrate feasible measure combinations that lead to lower emissions of eVTOLs than on-road vehicles. These findings shed light on the technology, regulation, and operation strategies necessary for eVTOLs to be not only efficient but also green.

Although eVTOLs are currently estimated to bring higher emissions than on-road vehicles, a set of low-hanging fruit technologies such as 300-Wh/kg batteries, which are highly likely to be deployed before 2025, can make eVTOL emissions lower than ICEV emissions. ICEVs still dominate 98% of the market stock in on-road transport [40]. Considering the extensive use of ICEVs, adopting eVTOLs has already emerged as an effective way to mitigate transport emissions.

A set of strategies for abating eVTOL emissions is needed to ensure the low-carbon transport transition. As our results suggest, the top priority should be to significantly reduce the electricity emission factor through the adoption of renewable power. With plausible battery improvement assumptions, a 0.25 kg CO_2_-eq/kWh power emission factor is needed to reach the breakeven between eVTOL and BEV emissions. Such a level is largely within the global targets in the timeframe of 2030 to 2040 [39]. However, despite continuous efforts, significant challenges remain in achieving these targets. Renewable energy sources, such as wind and solar, are intermittent and subject to seasonal fluctuations, necessitating large energy storage capacities to maintain grid stability [[41], [42], [43]]. The large-scale deployment of these technologies, coupled with necessary upgrades of grid infrastructure, demands significant time and capital investment [44]. This also adds to the complexity of grid dispatch and load management, requiring increased efforts in developing advanced algorithms and control systems.

Battery technology lies at the heart of eVTOL development, both from the technology feasibility and CO_2_ abatement perspectives. It is therefore urgent to improve battery technology, especially in the near and medium term, since substantial reductions in electricity emission factors cannot be easily achieved. Increasing battery-specific energy to above 400 Wh/kg and extending battery life are key targets for eVTOL-specific batteries. NCM batteries and SSBs are candidate technologies for eVTOL utilization, though both face challenges in reaching these goals.

For NCM batteries, adopting Ni-rich materials can enhance specific energy, whereas this raises safety concerns. Safety is a priority for eVTOL batteries. Addressing the safety issues of NCM batteries requires extensive R&D efforts, which introduces uncertainty regarding their large-scale utilization in eVTOLs. Innovations in both active safety (e.g., big-data-based detection and fault diagnosis) and passive safety (e.g., improved internal structure) are essential [45]. Extending cycle life also presents difficulties for NCM batteries, particularly considering the fast-changing discharge demands during eVTOL flights under complex weather conditions. This requires both material advancements (e.g., changing material content) and improved battery management systems [46].

While SSBs show great potential to achieve high specific energy, enhanced safety, and extended cycle life simultaneously, they face numerous challenges, notably their low technology readiness level. SSBs remain at the laboratory or pilot production stage and have not yet been commercially scaled [47,48]. Producing SSBs requires new techniques and equipment, demanding significant time and financial investment [35,48]. Moreover, finding a suitable material for the solid electrolyte that balances performance and cost remains difficult. Issues such as poor contact or chemical incompatibility between the solid electrolyte and electrode interface further degrade battery performance and cycle life.

High operation efficiency, especially high load factor, is essential in that it dilutes eVTOLs’ large life cycle emissions into a relatively low levelized level. To maintain this, consumer willingness for taking eVTOL transport should be promoted. Safety is the primary concern for most consumers to choosing eVTOLs [49]. Efficient but strict airworthiness certification is required, particularly considering the potential safety hazard of high-specific-energy NCM batteries. Demonstration operation is also suggested to improve passengers’ confidence and willingness to adopt eVTOL services. Another significant barrier to consumer acceptance is the high cost. The manufacturing cost of an eVTOL is estimated at over $600,000 [2], nearly 20 times that of a Tesla Model 3. The average fare of eVTOL service, considering both production and operational expenses, is projected to be $1.5 to $3.0 per passenger-km [10], substantially higher than on-road taxi services. Improving battery-specific energy, which can cut down manufacturing and energy costs, is crucial to reducing eVTOL costs.

Many other factors also impact consumer acceptance of eVTOLs. Current regulations on energy reserve, as discussed above, will necessitate larger battery capacities in eVTOLs, leading to higher GHG emissions and costs and ultimately, lower consumer acceptance. Moreover, regulations such as no-fly zones, while ensuring urban safety, limit operational areas and application scenarios of eVTOLs, reducing their convenience for potential users. Continuously evolving regulations, comsidering technology improvements, are needed to promote eVTOL adoption. Noise is a significant obstacle to integrating eVTOLs into people’s daily lives [50]. The large-scale deployment of eVTOLs in cities could lead to prolonged noise exposure, negatively affecting public health. The cruising altitude and noise levels of eVTOLs, particularly in urban areas, must be regulated, necessitating well-designed noise pollution tests. Furthermore, the large size of eVTOLs, particularly those with multiple rotors, poses challenges for vertiport construction in cities. One potential solution could be utilizing building rooftops to serve as vertiport sites, offering space in urban environments.

Beyond the GHG reduction potential on a per-passenger-km basis, the adoption of eVTOLs can also create emissions reduction opportunities on the system level. For example, eVTOLs normally operate straight between the origin and the destination, while on-road vehicles typically have to travel longer due to complex road networks. This consequently leads to lower required traveling distance and GHG emissions for eVTOLs. Furthermore, a partial shift to air transport by eVTOLs can reduce traffic congestion and accidents, bringing considerable GHG reduction opportunities [3]. These potentials should be fully recognized when shaping future eVTOL development strategies.

## 5 Conclusion

Despite the benefits in transport efficiency, the potentially high emissions of eVTOLs have become one major concern, necessitating comprehensive estimation. To address this research gap, this study proposes a component-level LCA framework, which considers both WTW and CTG stages and is able to calculate eVTOL emissions across varied technology and operation scenarios. This framework, with detailed life cycle inventory provided, can serve as the foundation for future emissions studies on eVTOLs and can be expanded to assess other electric aircraft.

Our findings show that the life cycle emissions from current mainstream eVTOLs, under base case assumptions, are 0.24 kg CO_2_-eq/passenger-km, 95.2% higher than those from a BEV. Mitigating eVTOL emissions and narrowing this emissions gap will require cleaner electricity and advanced battery technology. These provide crucial insights into the climate impacts of the eVTOL industry, highlight its potential to play a role in future green transport, and offer valuable, achievable strategies for reducing eVTOL emissions to support eVTOL development.

While this study provides a comprehensive estimation of the emissions from eVTOLs, there are limitations that future studies could address. First, eVTOLs come in various configurations (tilt-rotor, lift & cruise, multi-rotor, etc.), whereas this study focuses on only one of them. Further research on other configurations is essential. Second, different application scenarios could result in distinct eVTOL emissions outcomes, necessitating more comprehensive studies that cover a broader range of eVTOL use cases.

## Declaration of competing interest

The authors declare that they have no conflicts of interest in this work.

## Appendix Supplementary materials

Image, application 1
