# Supplementary material for: Adopting electric vertical takeoff and landing aircraft for green transport transformation
Source: Fundam Res. 2025 Apr 11;6(4):2621–33. doi: 10.1016/j.fmre.2025.04.002 (PMC13424162; doi:10.1016/j.fmre.2025.04.002)
Supplement: Supplementary file 1 [file mmc1.docx]

**Supplemental Figures**

**
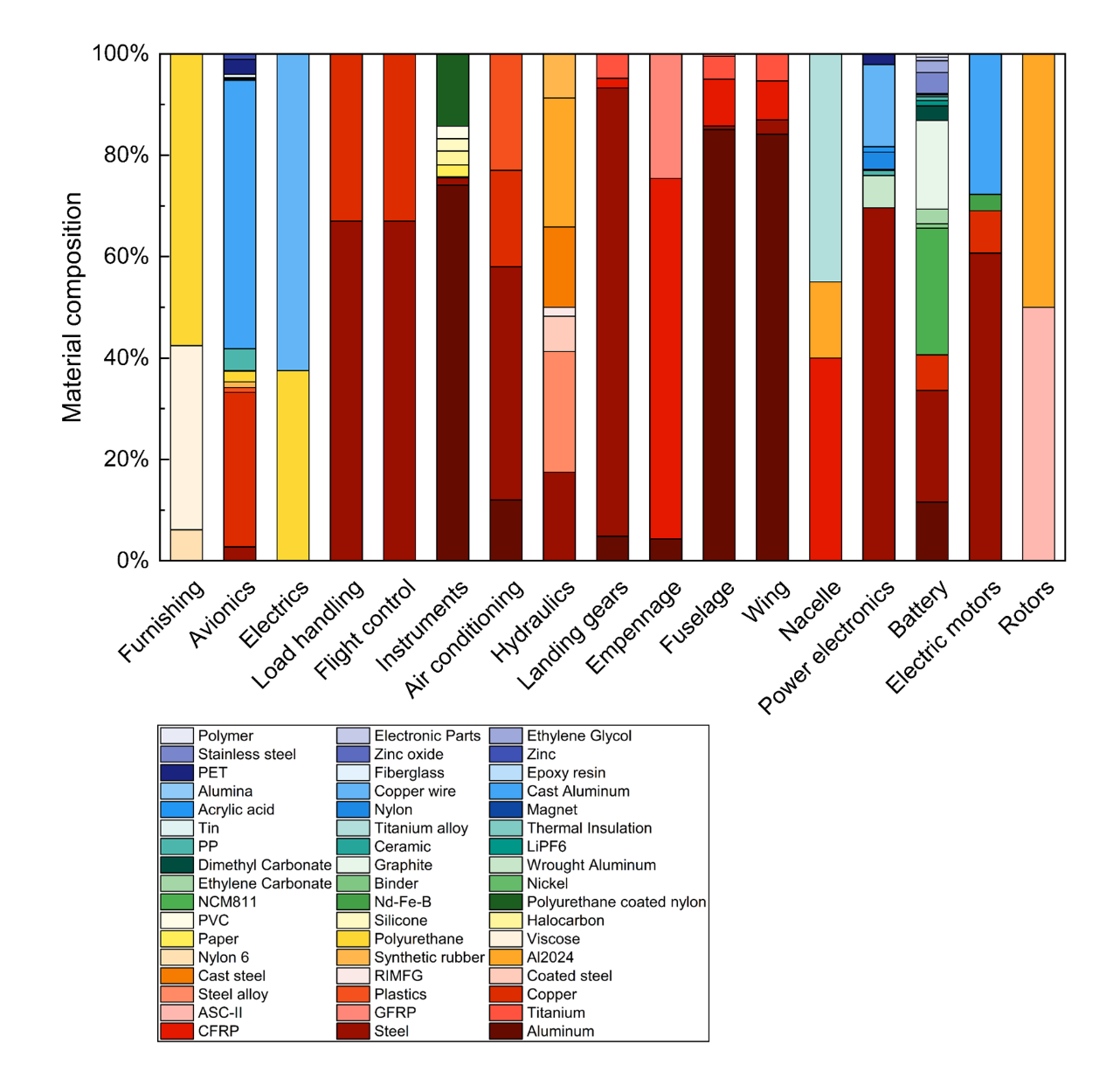
**

**Figure S1.** Bill of materials for eVTOL components. CFRP: Carbon Fiber Reinforced Plastic; GFRP: Glass Fiber Reinforced Plastic; PP: Polypropylene; PET: Polyethylene Terephthalate; RIMFG: Resin Impregnated Micro Fiber Glass; NCM: Nickel Cobalt Manganese.


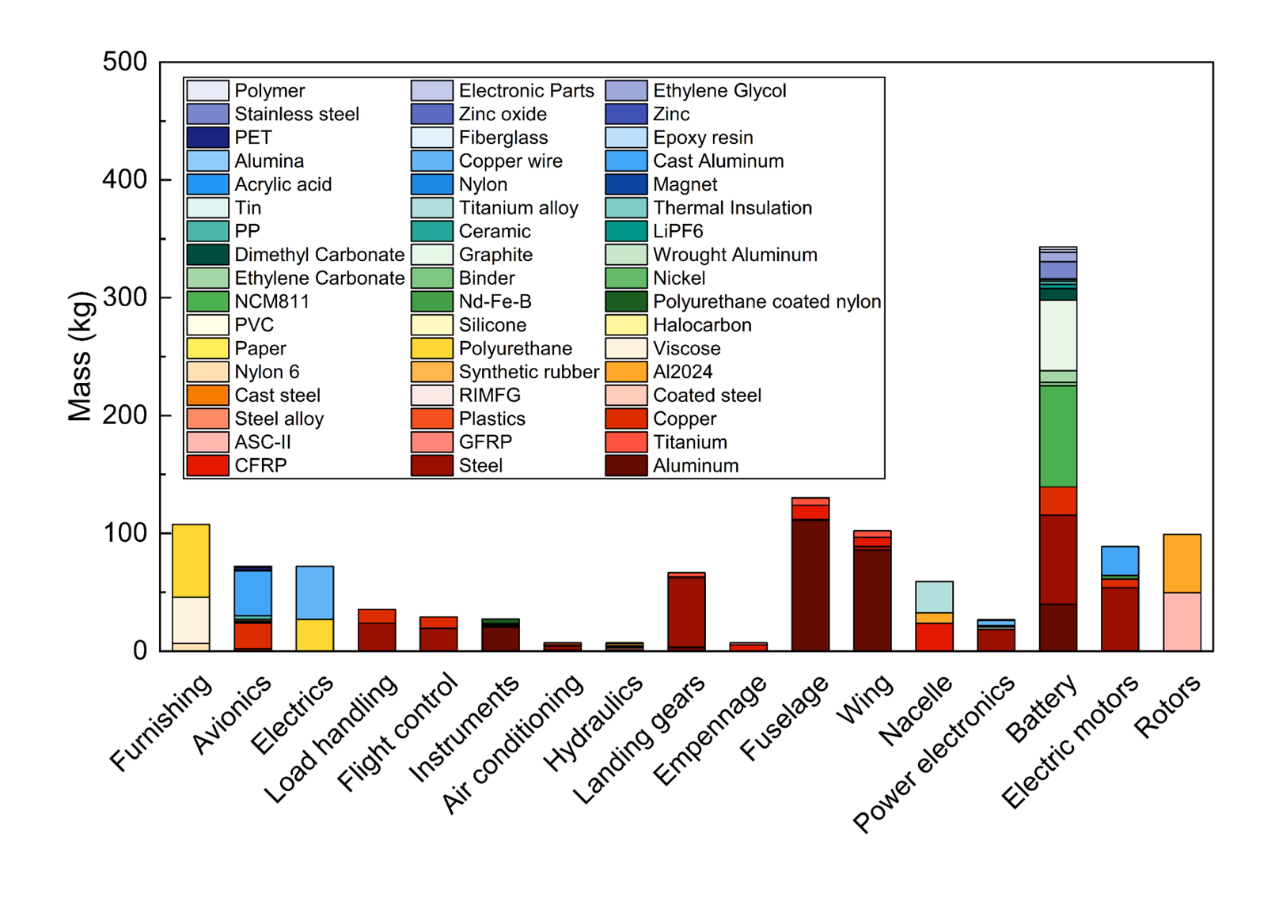


**Figure S2.** Material mass distribution of each component (battery specific energy of 300 Wh/kg).


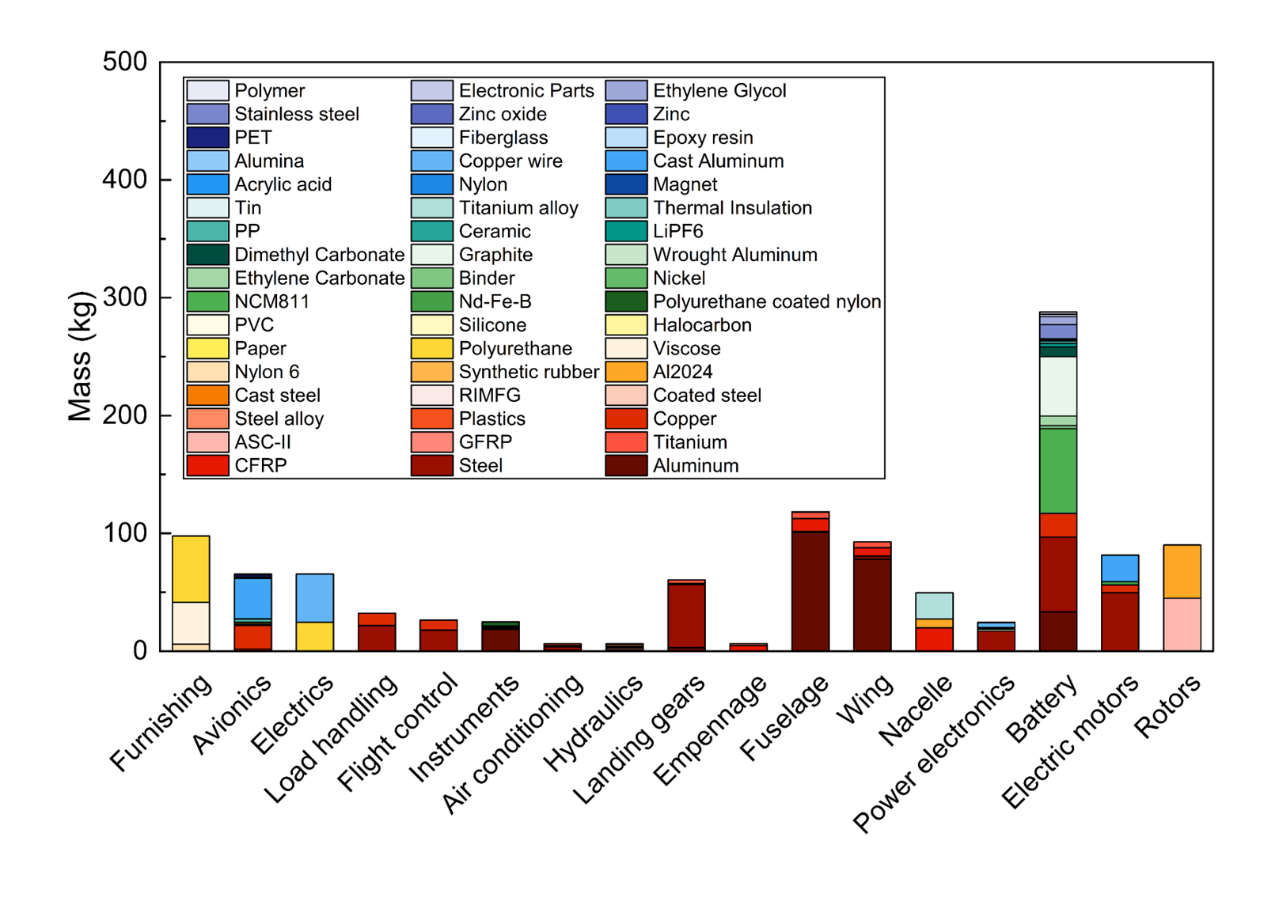


**Figure S3.** Material mass distribution of each component (battery specific energy of 350 Wh/kg).


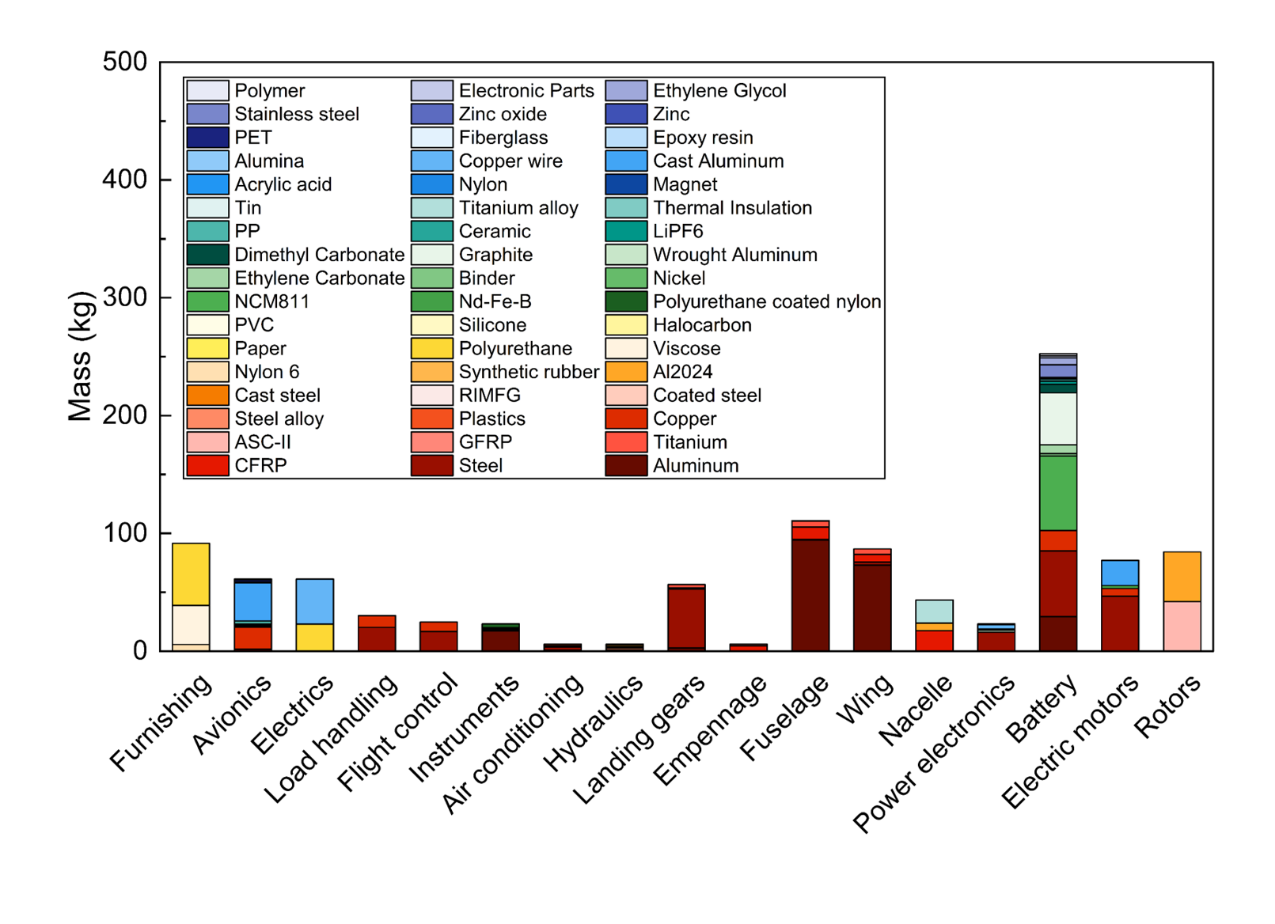


**Figure S4.** Material mass distribution of each component (battery specific energy of 400 Wh/kg).


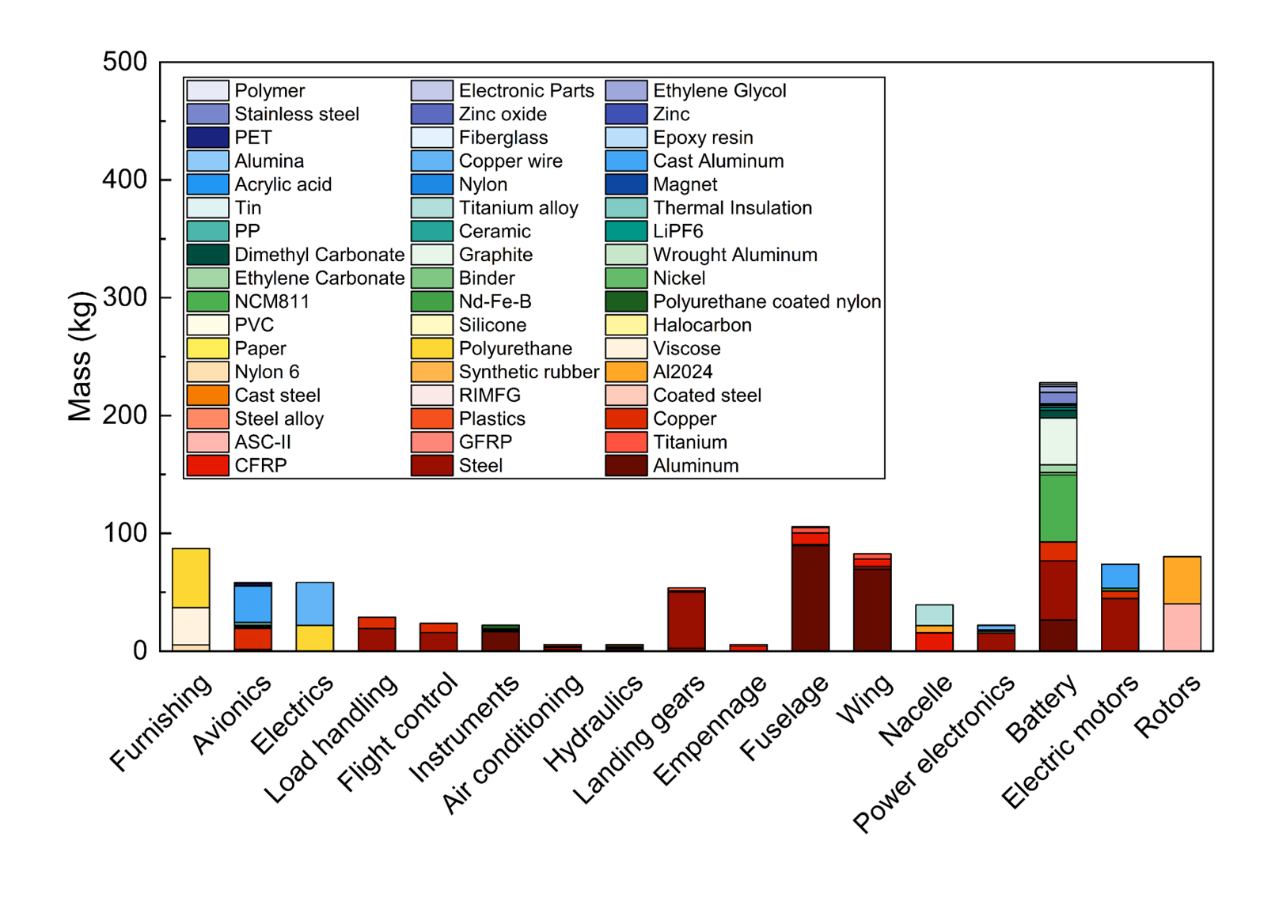


**Figure S5.** Material mass distribution of each component (battery specific energy of 450 Wh/kg).


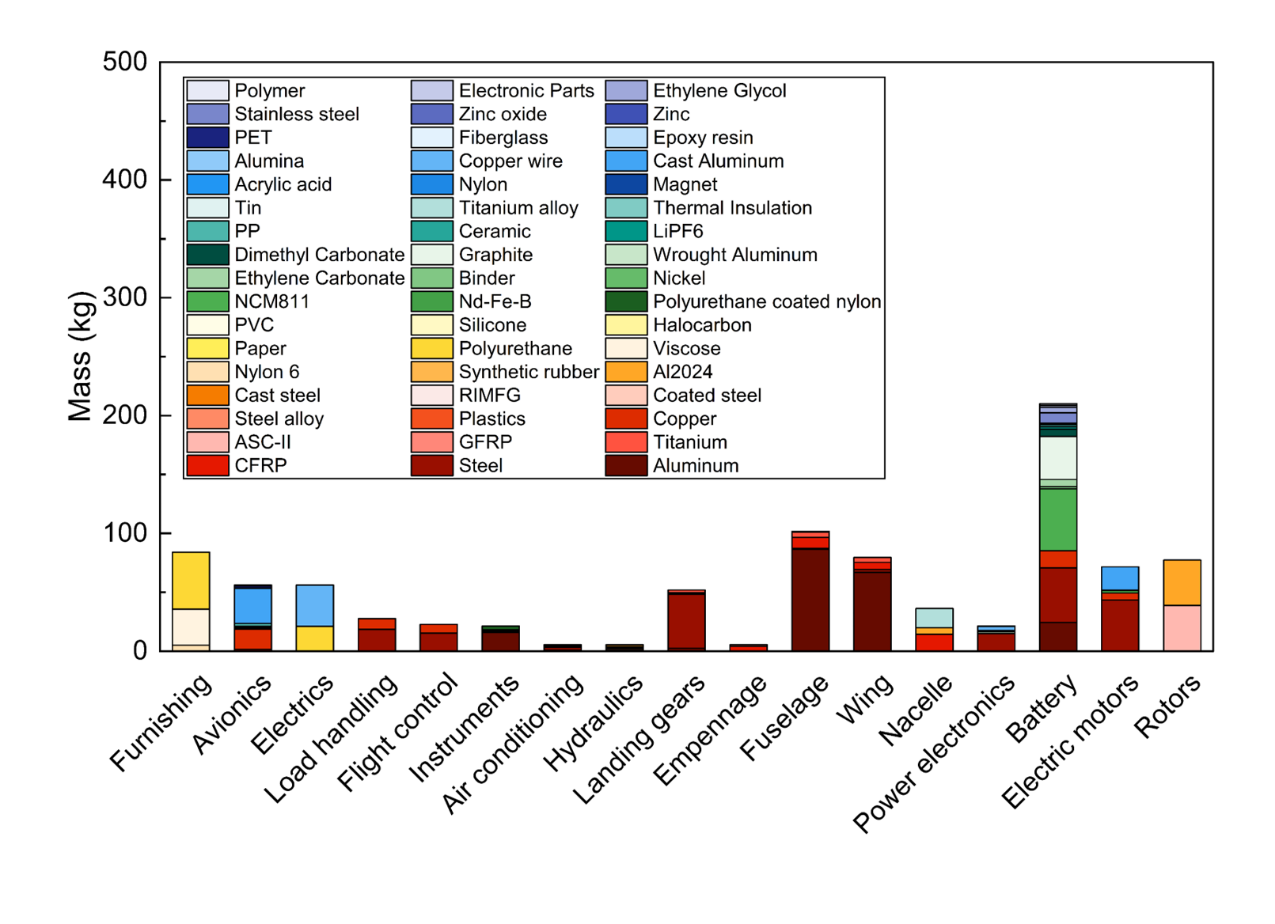


**Figure S6.** Material mass distribution of each component (battery specific energy of 500 Wh/kg).


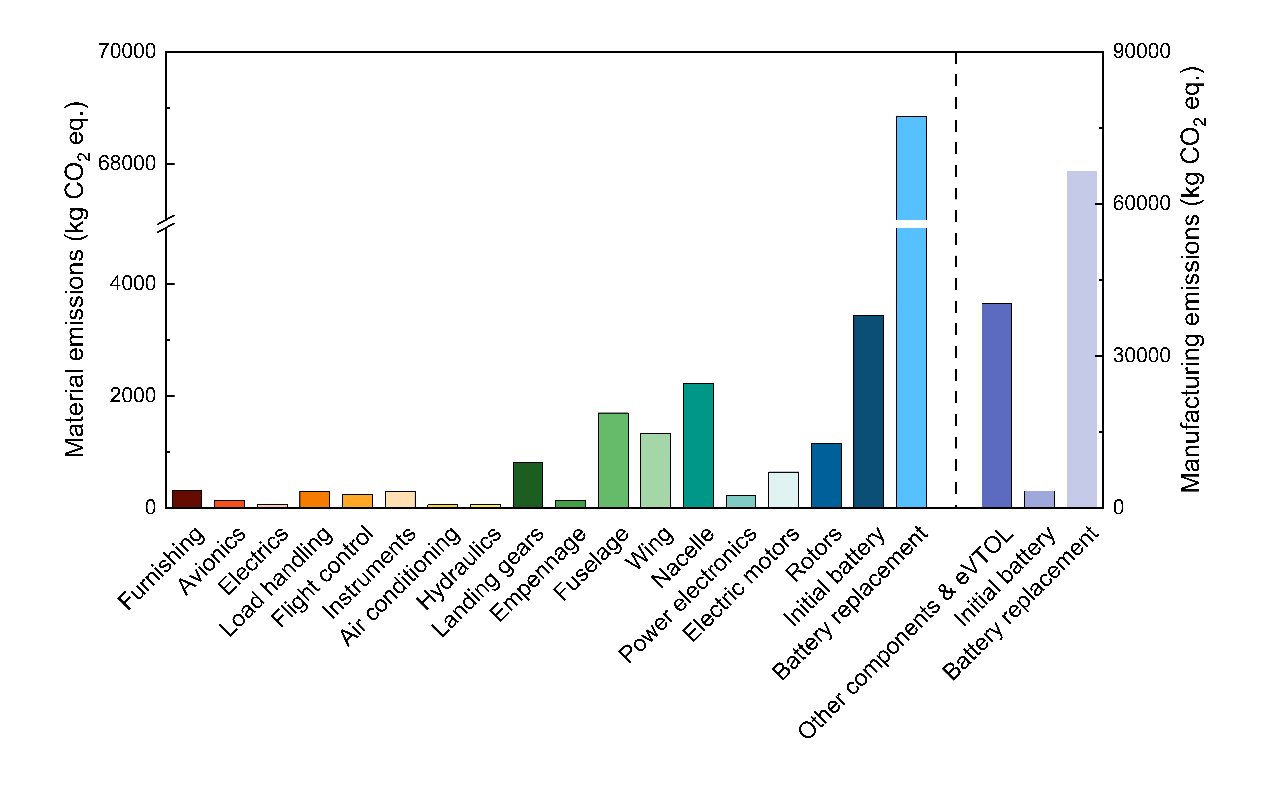


**Figure S7.** CTG emissions from eVTOLs (battery specific energy: 300 Wh/kg, electricity emission factor: base case, battery life: base case).


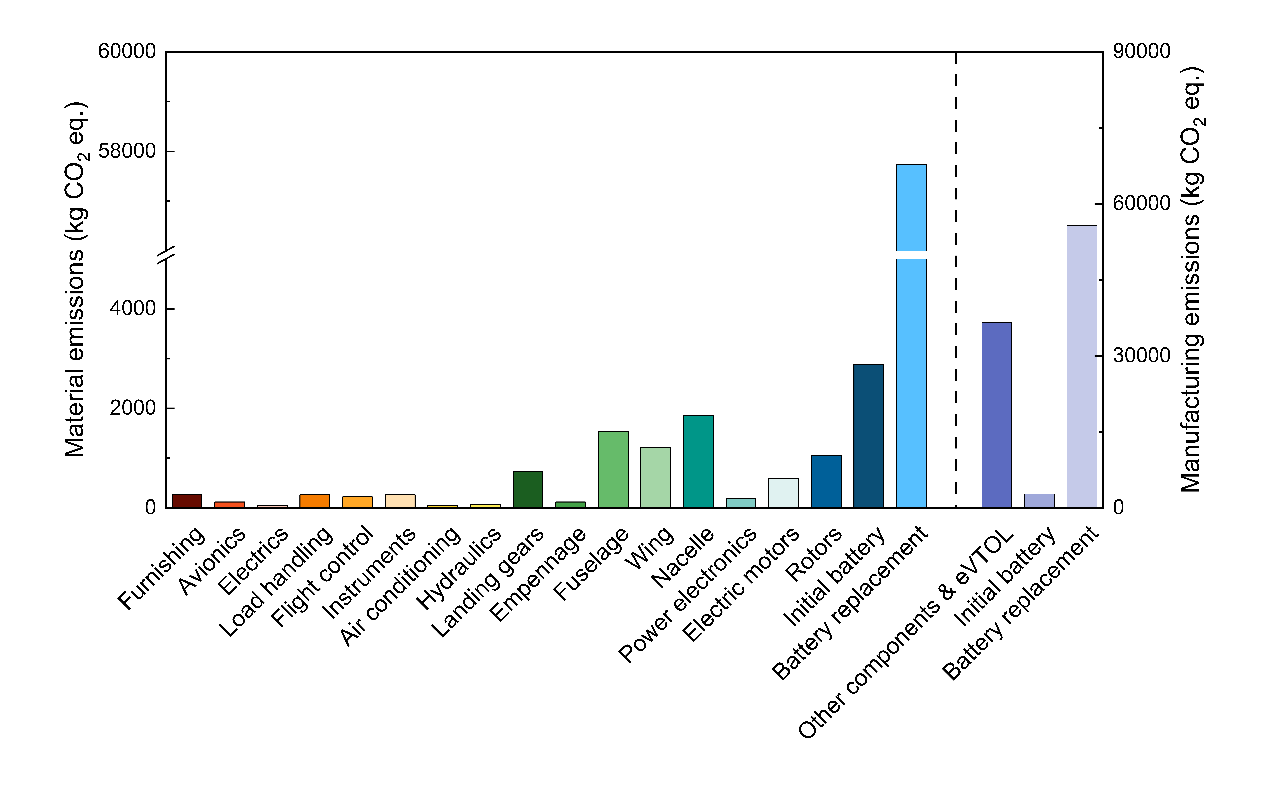


**Figure S8.** CTG emissions from eVTOLs (battery specific energy: 350 Wh/kg, electricity emission factor: base case, battery life: base case).


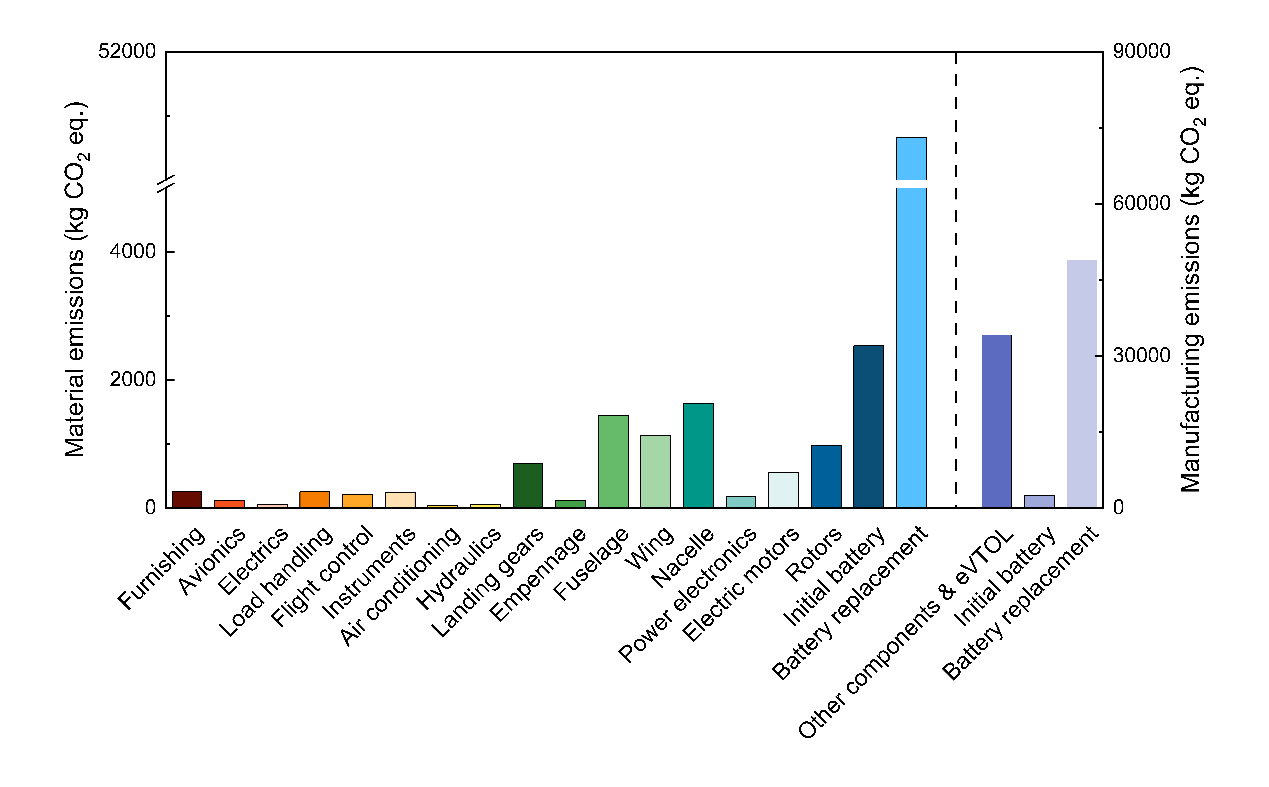


**Figure S9.** CTG emissions from eVTOLs (battery specific energy: 400 Wh/kg, electricity emission factor: base case, battery life: base case).


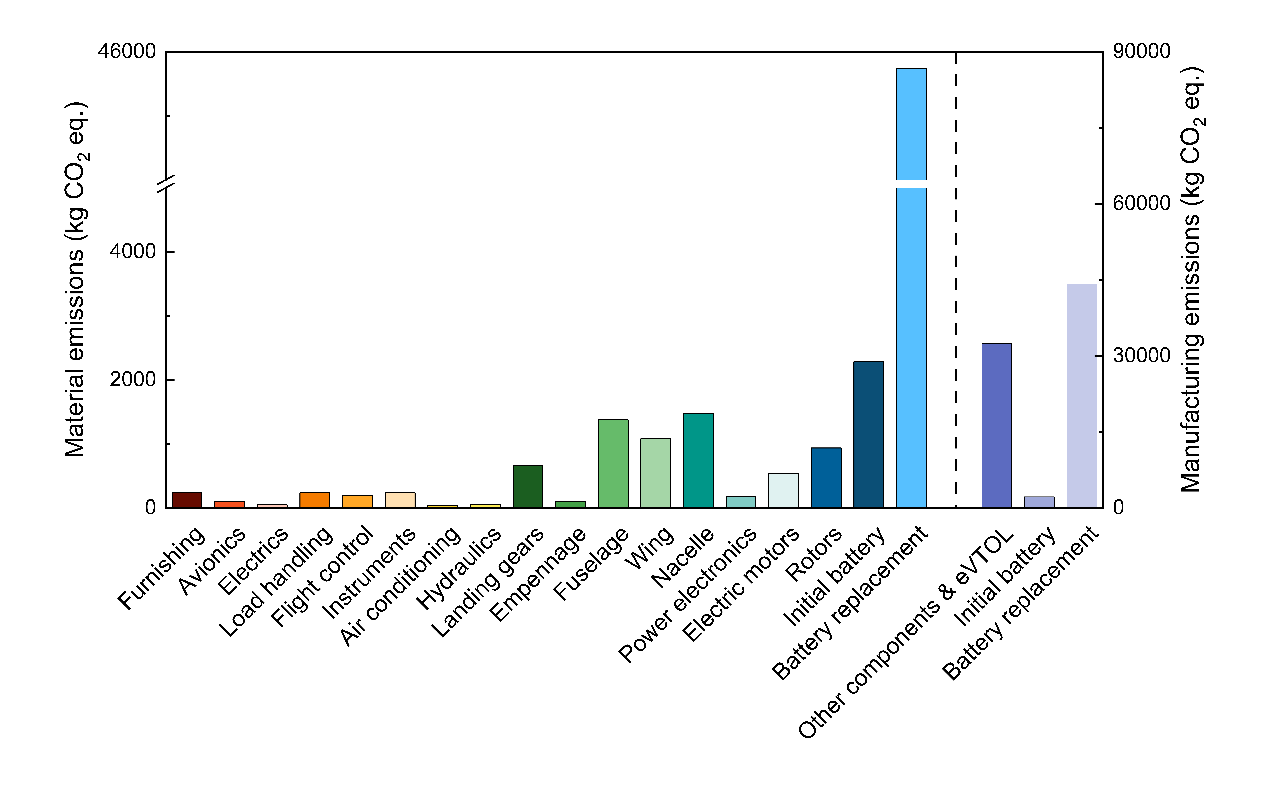


**Figure S10.** CTG emissions from eVTOLs (battery specific energy: 450 Wh/kg, electricity emission factor: base case, battery life: base case).


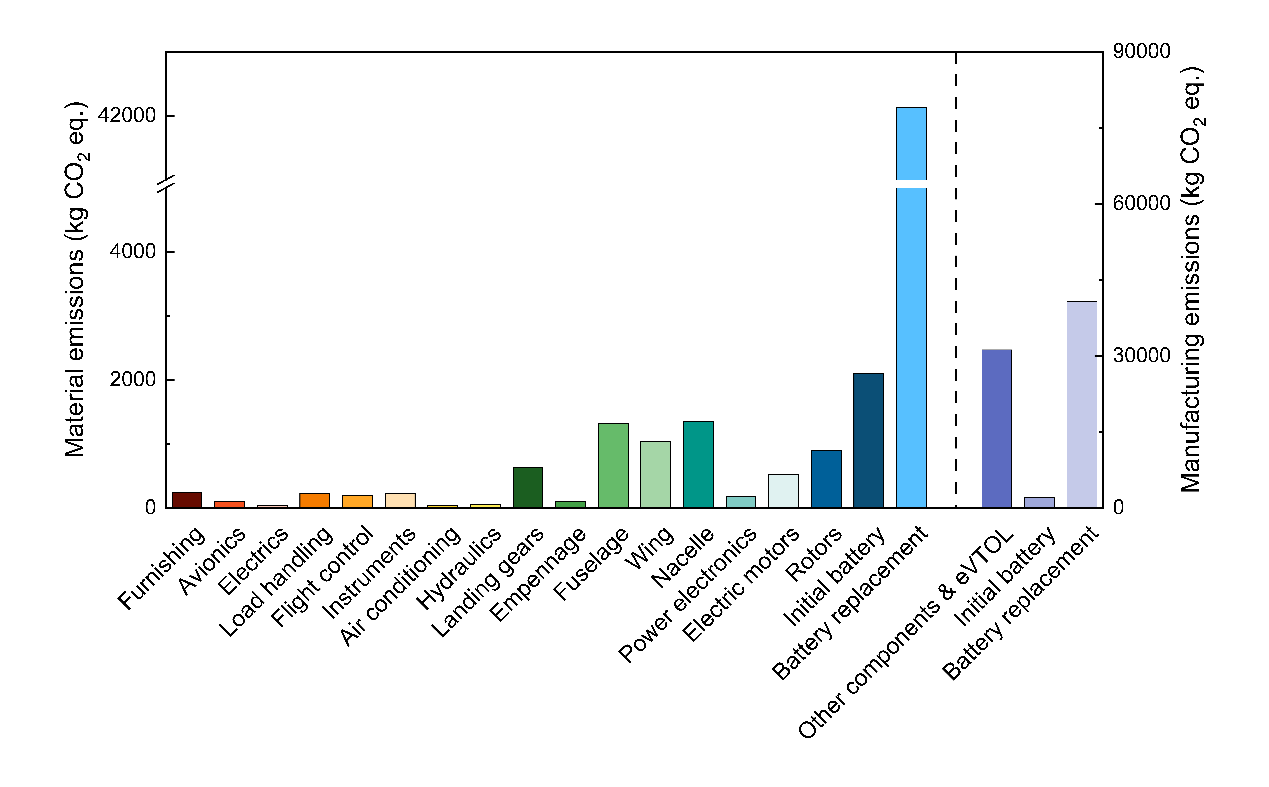


**Figure S11.** CTG emissions from eVTOLs (battery specific energy: 500 Wh/kg, electricity emission factor: base case, battery life: base case).


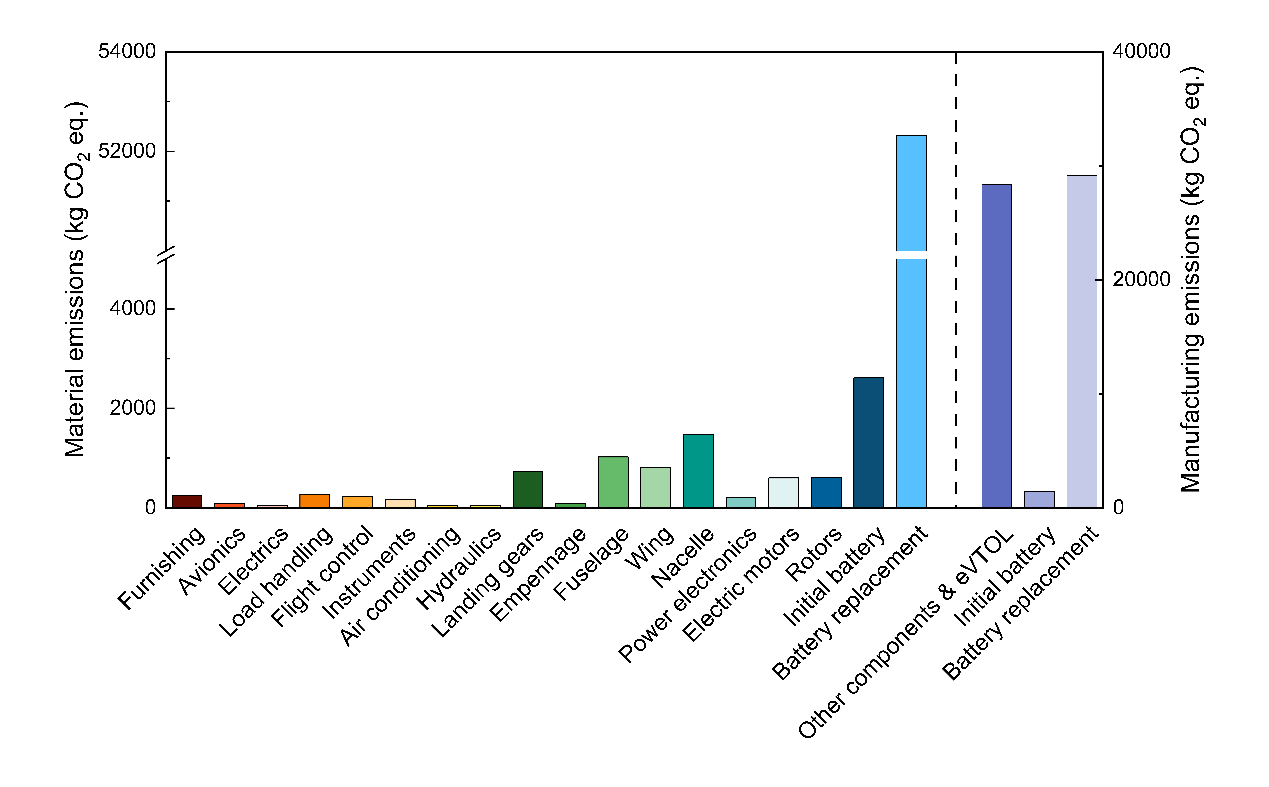


**Figure S12.** CTG emissions from eVTOLs (battery specific energy: 300 Wh/kg, electricity emission factor: 0.25 kg CO_2_/kWh, battery life: base case).


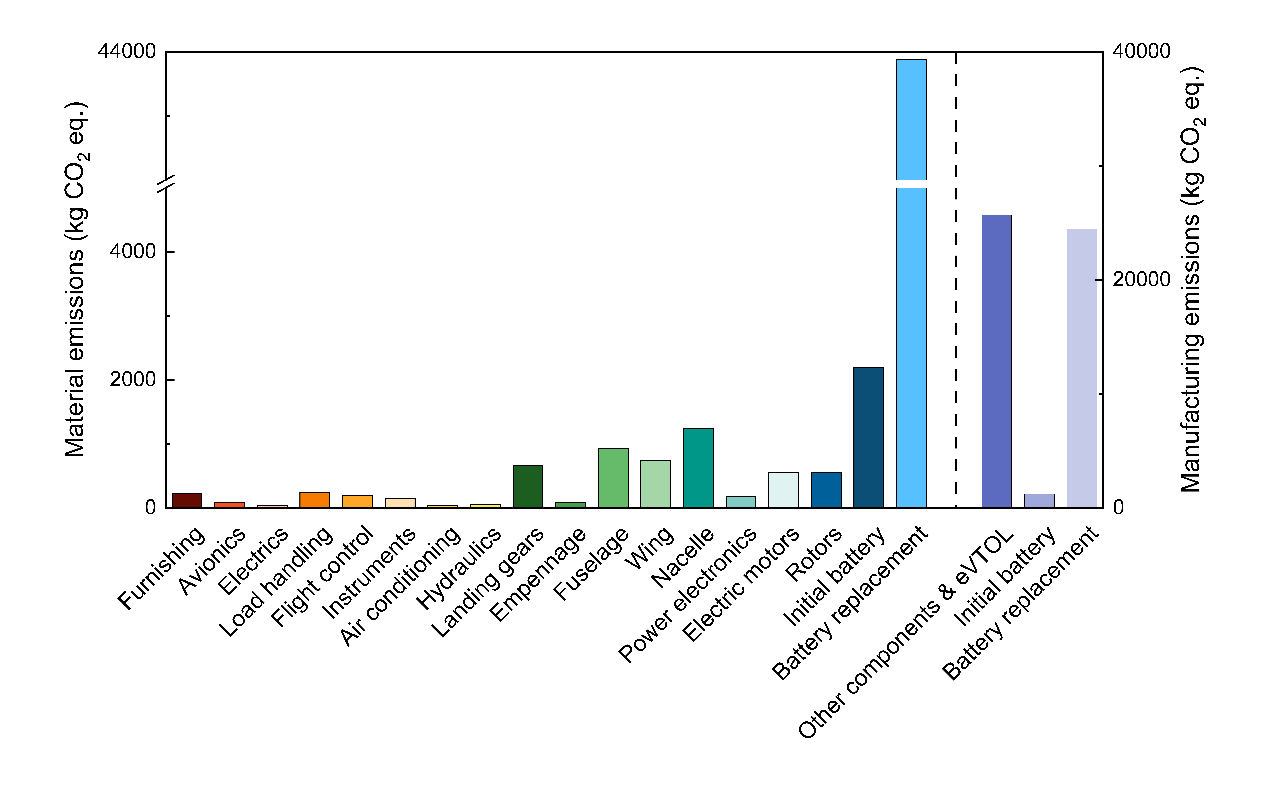


**Figure S13.** CTG emissions from eVTOLs (battery specific energy: 350 Wh/kg, electricity emission factor: 0.25 kg CO_2_/kWh, battery life: base case).


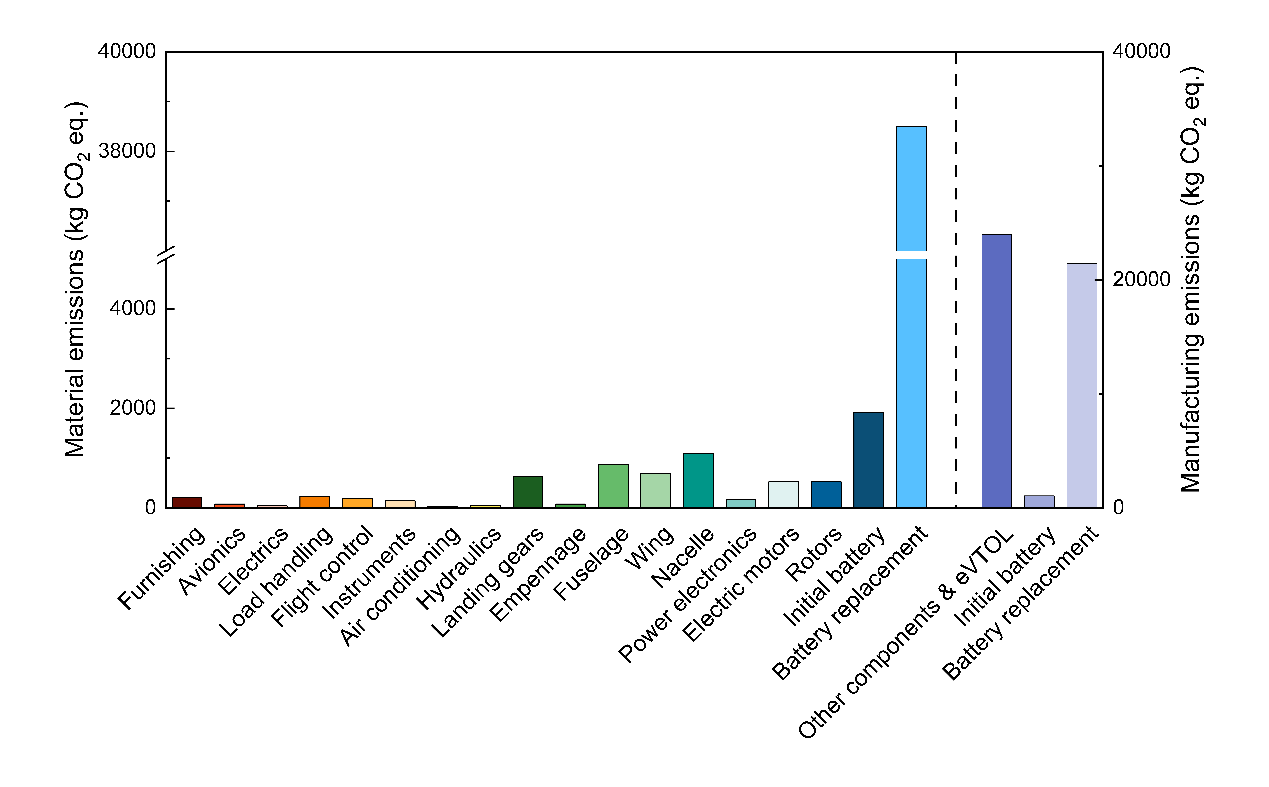


**Figure S14.** CTG emissions from eVTOLs (battery specific energy: 400 Wh/kg, electricity emission factor: 0.25 kg CO_2_/kWh, battery life: base case).


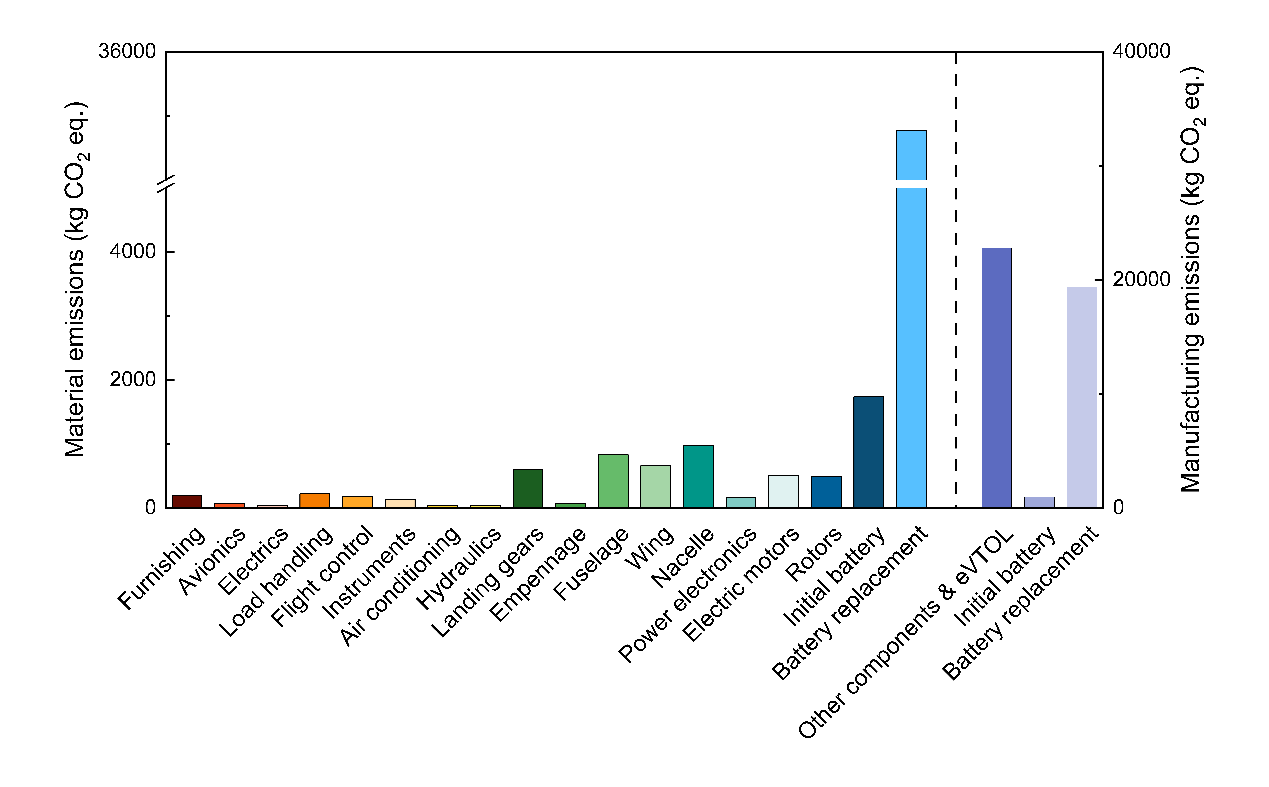


**Figure S15.** CTG emissions from eVTOLs (battery specific energy: 450 Wh/kg, electricity emission factor: 0.25 kg CO_2_/kWh, battery life: base case).


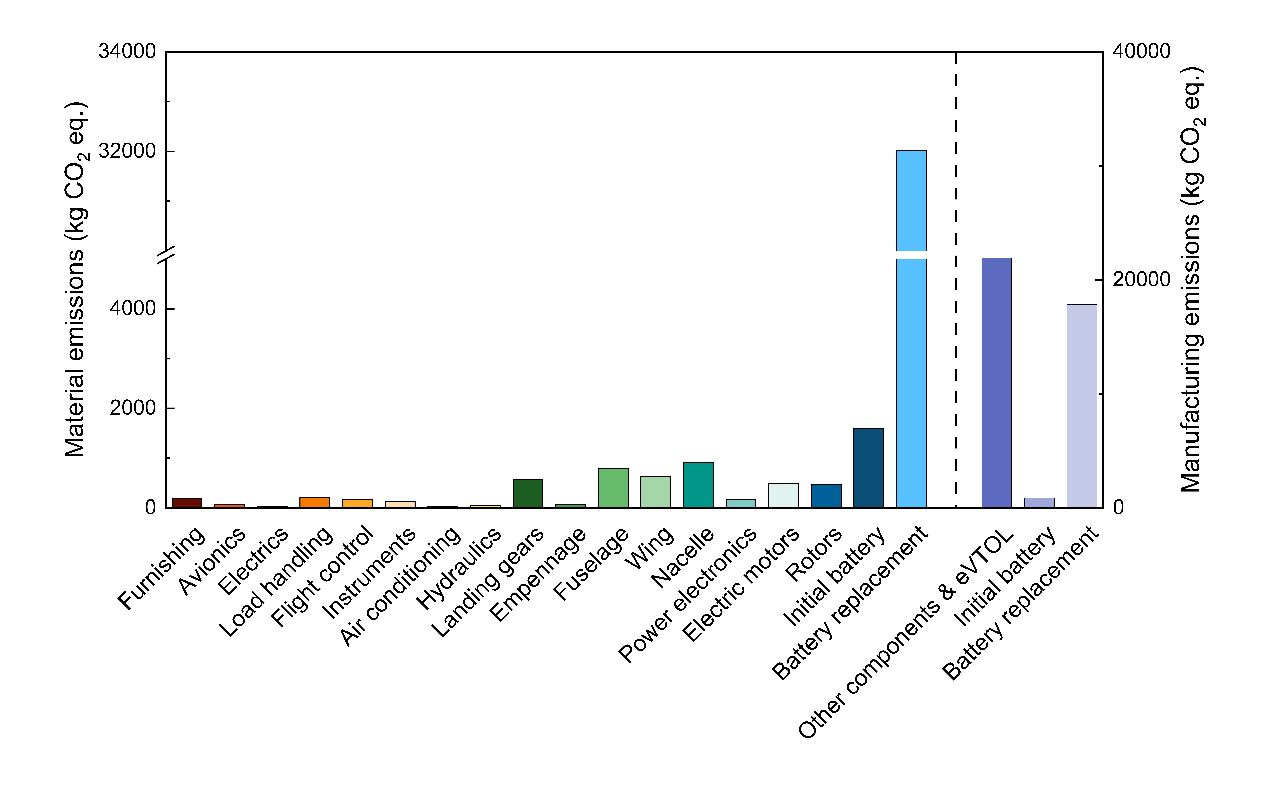


**Figure S16.** CTG emissions from eVTOLs (battery specific energy: 500 Wh/kg, electricity emission factor: 0.25 kg CO_2_/kWh, battery life: base case).


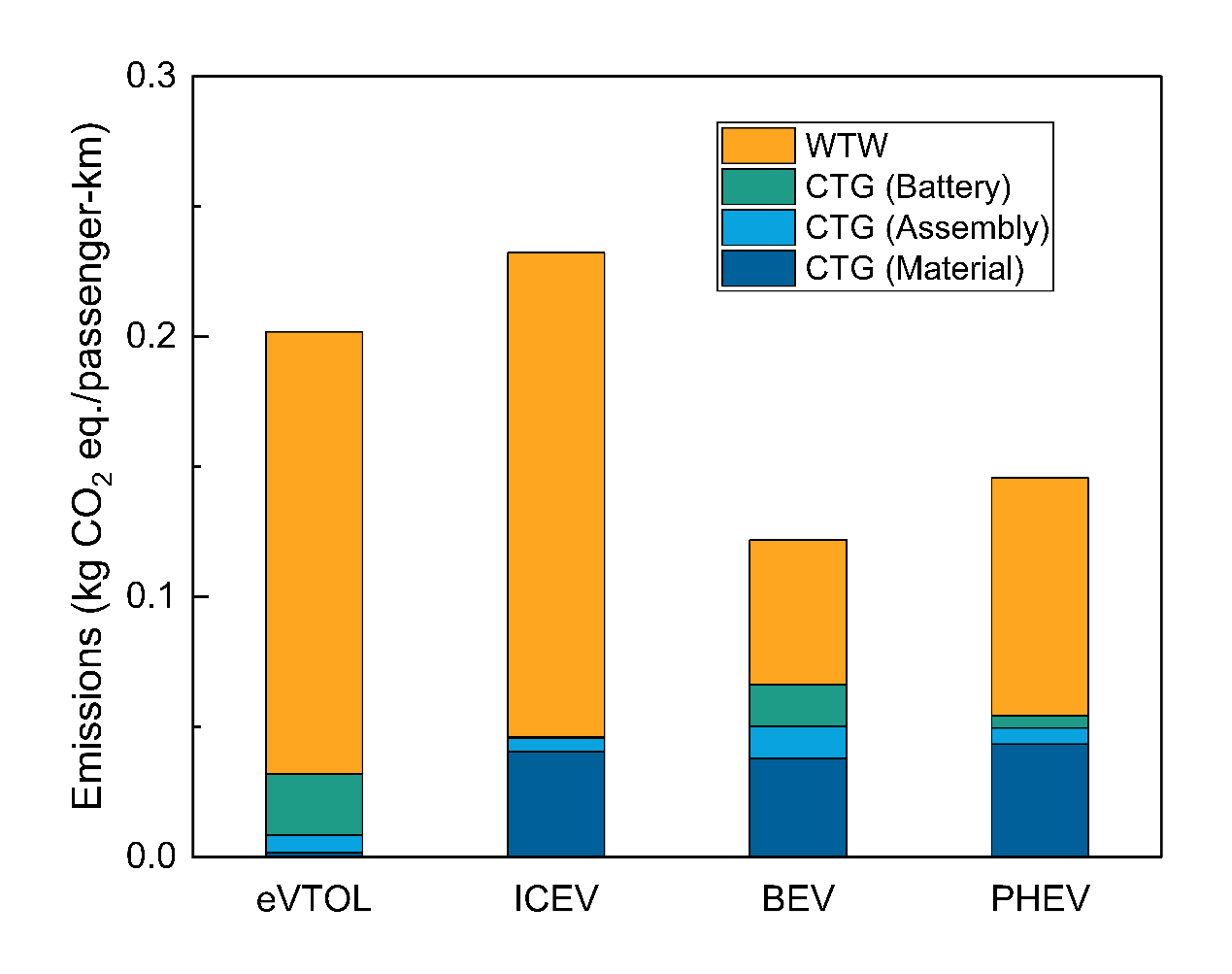


**Figure S17.** Life cycle emissions from eVTOLs and on-road vehicles (battery specific energy: 300 Wh/kg, electricity emission factor: base case, battery life: base case).


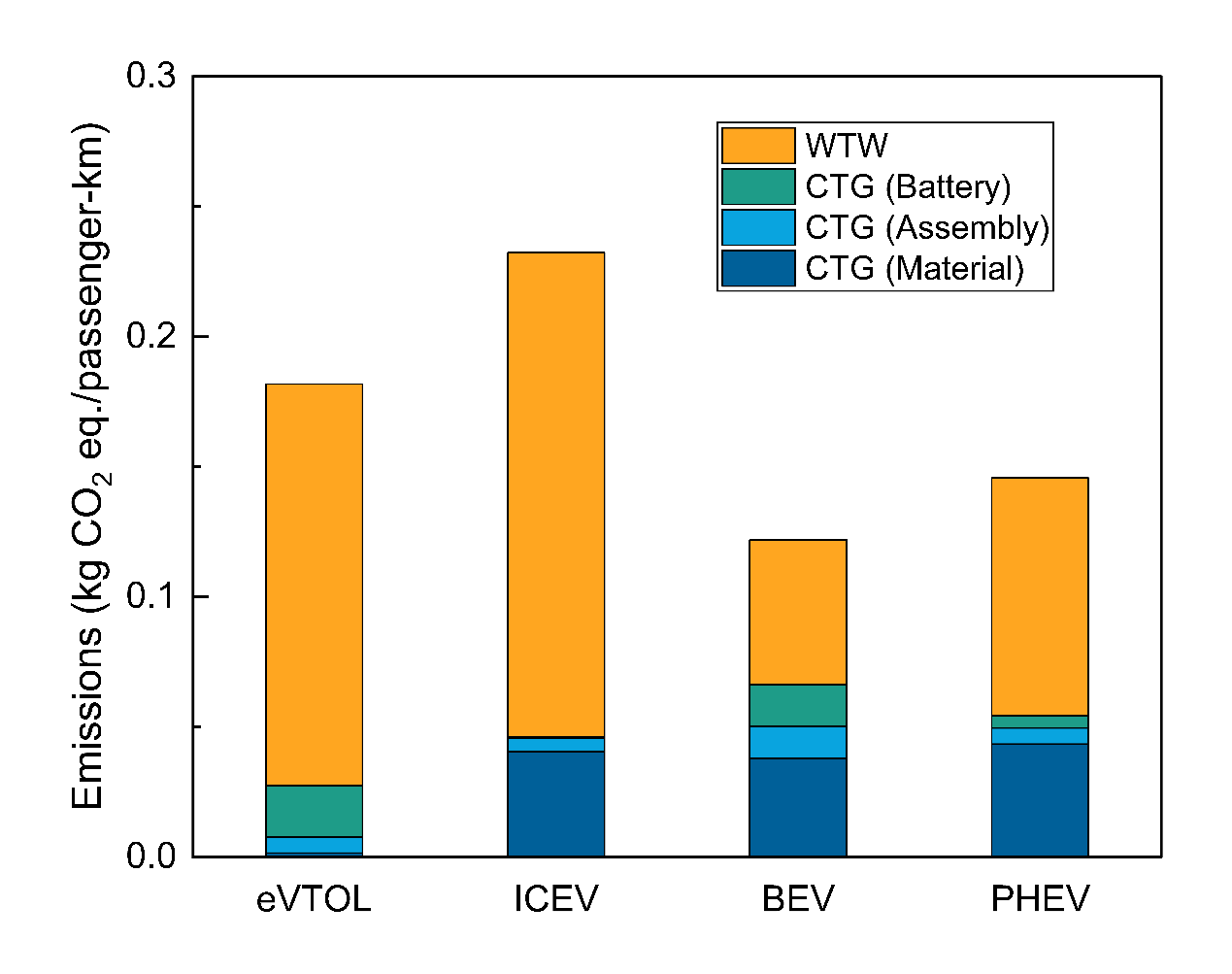


**Figure S18.** Life cycle emissions from eVTOLs and on-road vehicles (battery specific energy: 350 Wh/kg, electricity emission factor: base case, battery life: base case).


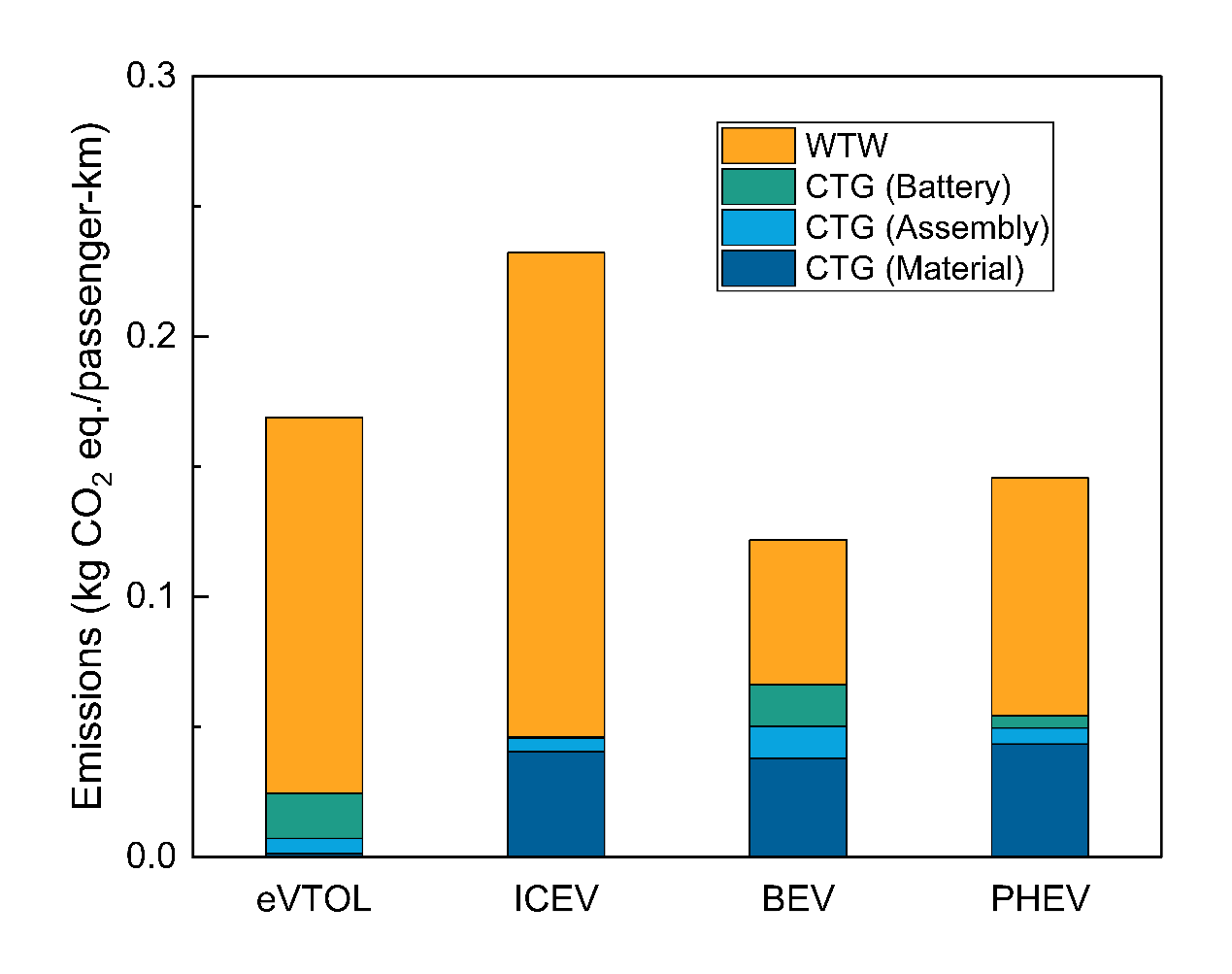


**Figure S19.** Life cycle emissions from eVTOLs and on-road vehicles (battery specific energy: 400 Wh/kg, electricity emission factor: base case, battery life: base case).


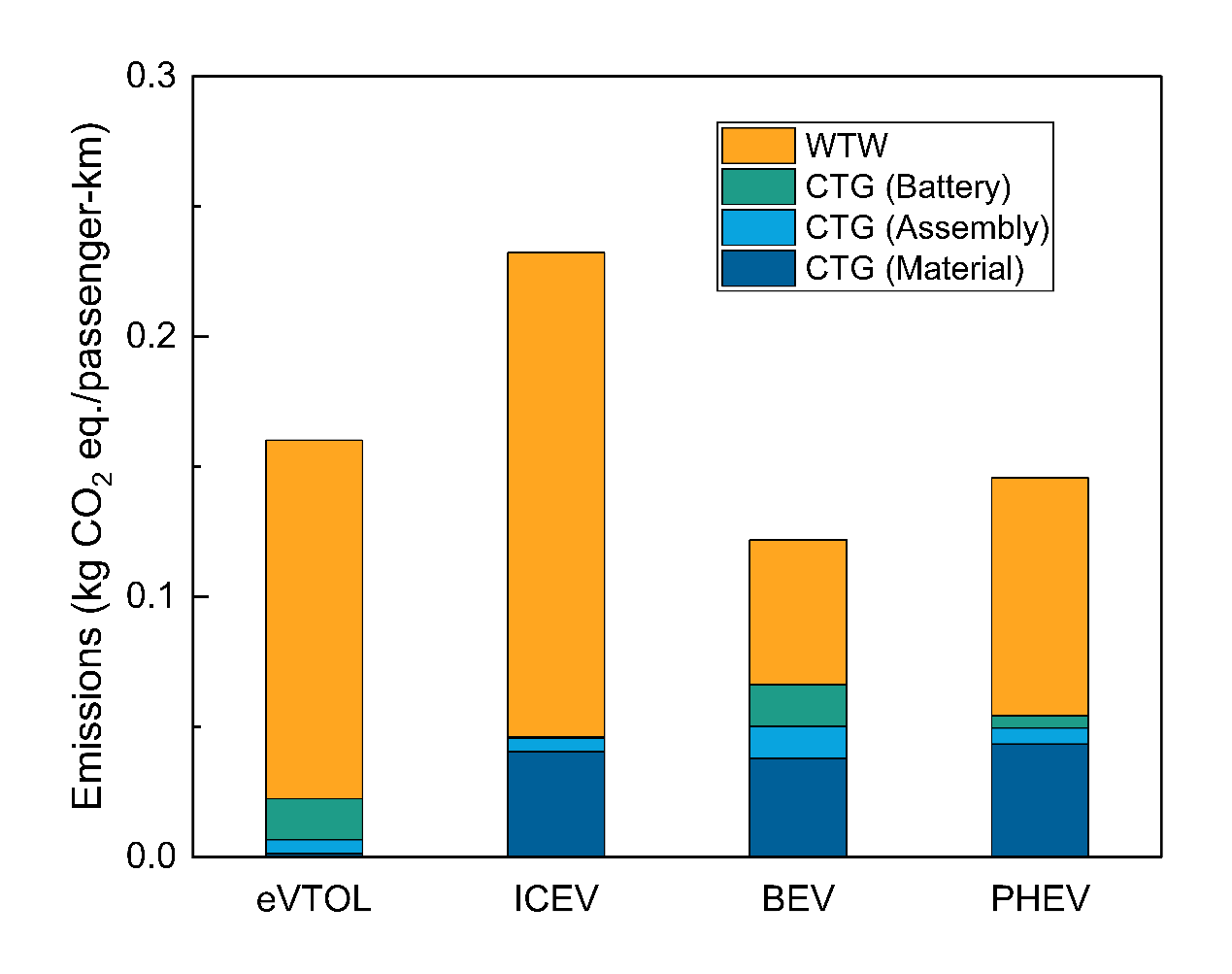


**Figure S20.** Life cycle emissions from eVTOLs and on-road vehicles (battery specific energy: 450 Wh/kg, electricity emission factor: base case, battery life: base case).


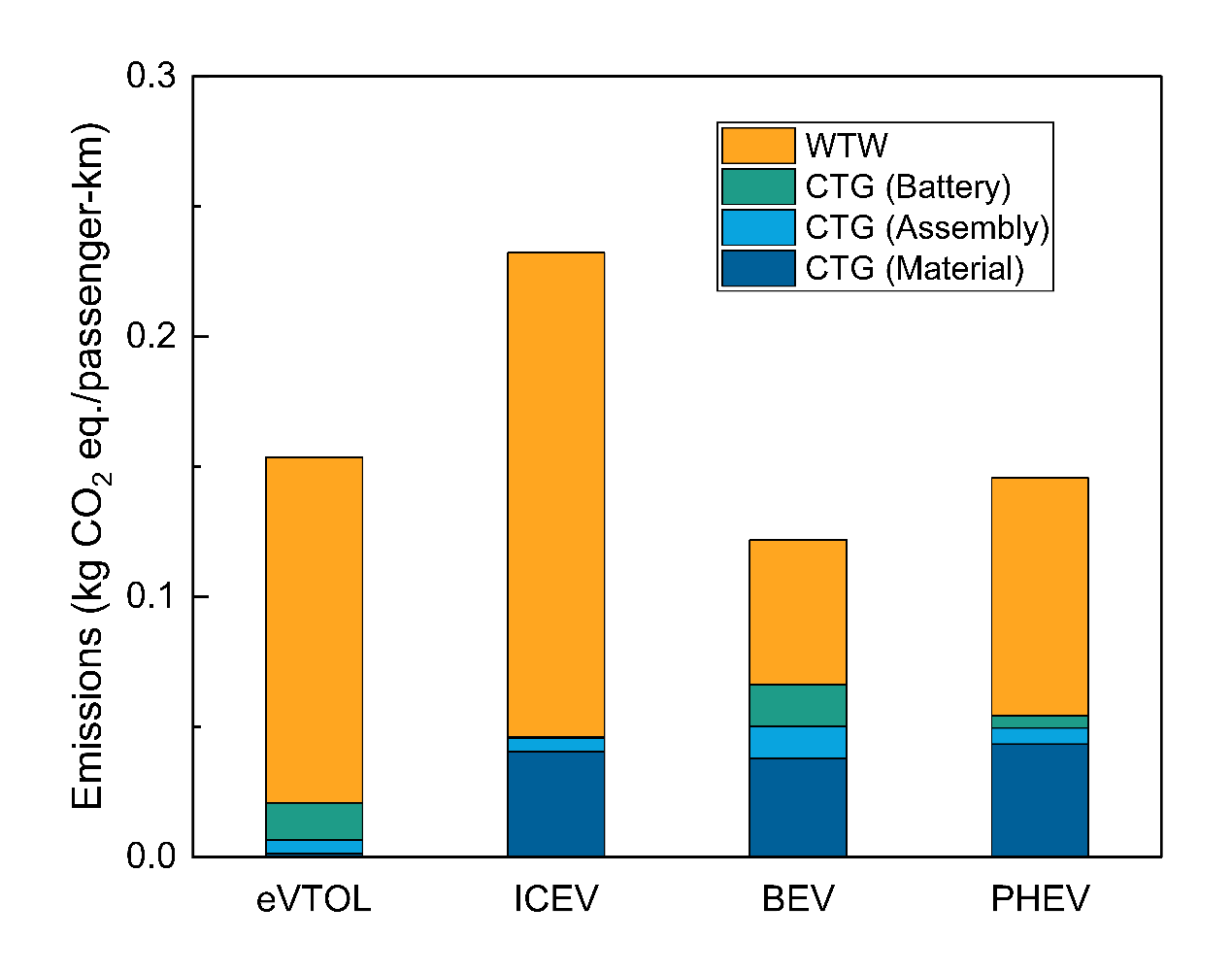


**Figure S21.** Life cycle emissions from eVTOLs and on-road vehicles (battery specific energy: 500 Wh/kg, electricity emission factor: base case, battery life: base case).


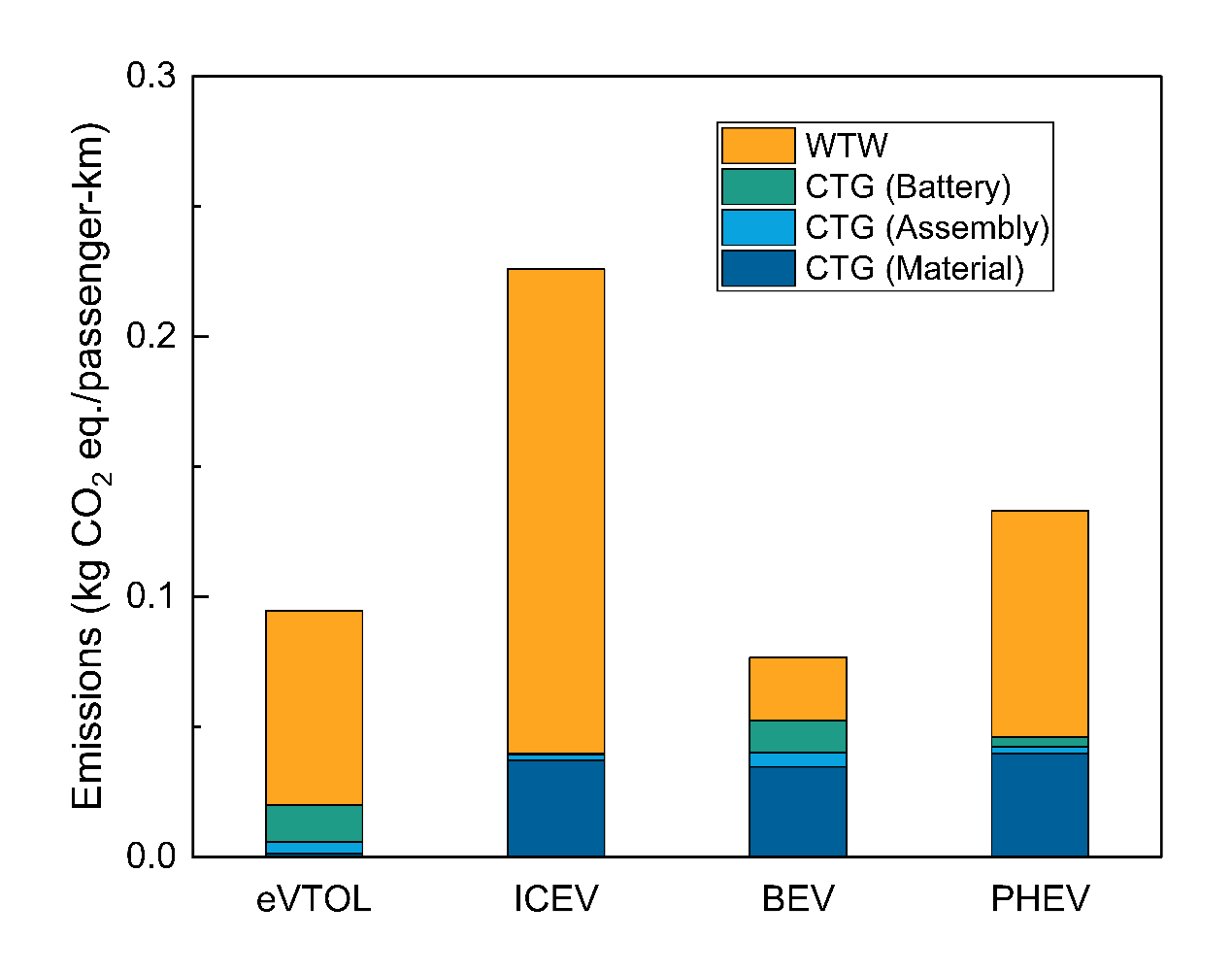


**Figure S22.** Life cycle emissions from eVTOLs and on-road vehicles (battery specific energy: 300 Wh/kg, electricity emission factor: 0.25 kg CO_2_/kWh, battery life: base case).


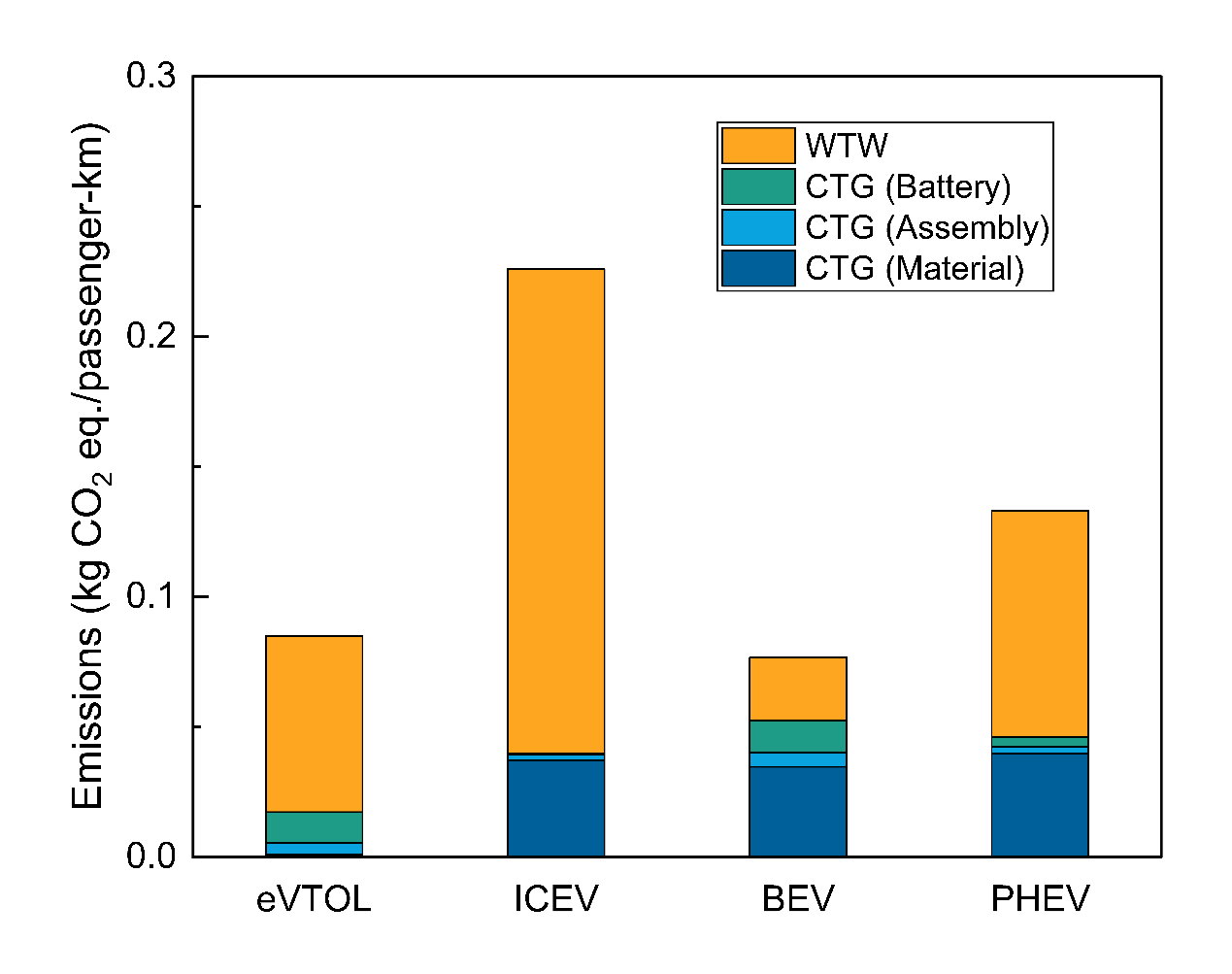


**Figure S23.** Life cycle emissions from eVTOLs and on-road vehicles (battery specific energy: 350 Wh/kg, electricity emission factor: 0.25 kg CO_2_/kWh, battery life: base case).


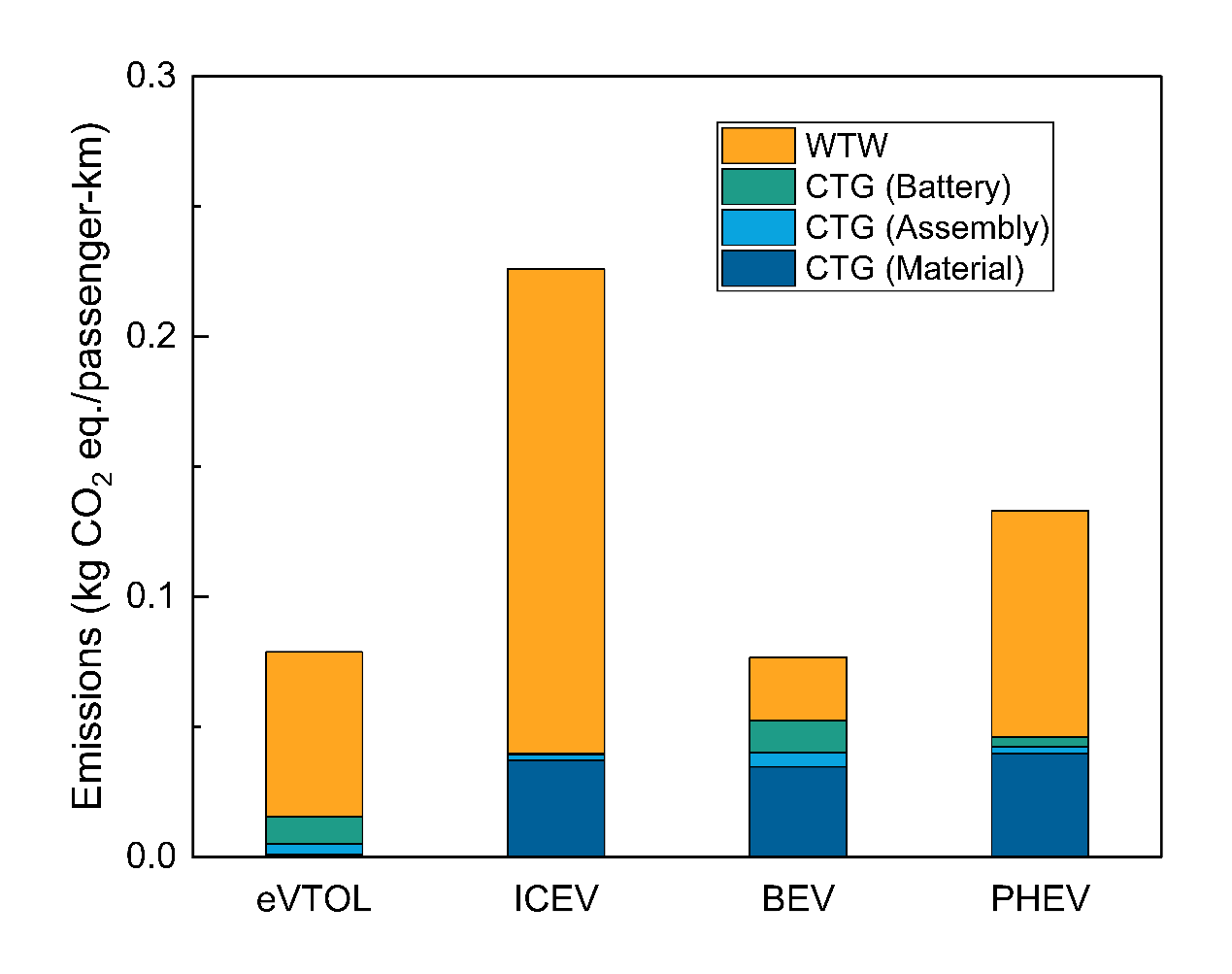


**Figure S24.** Life cycle emissions from eVTOLs and on-road vehicles (battery specific energy: 400 Wh/kg, electricity emission factor: 0.25 kg CO_2_/kWh, battery life: base case).


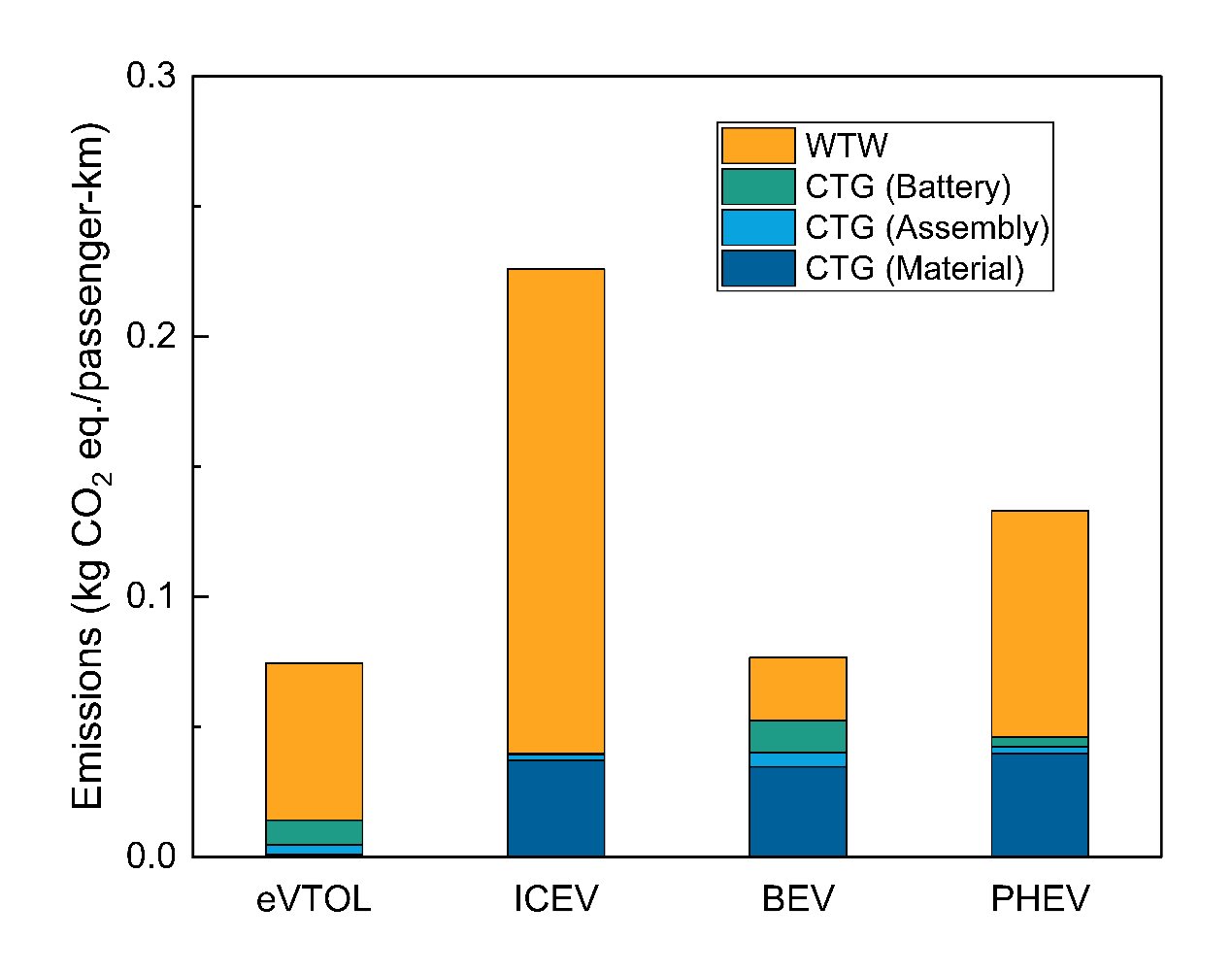


**Figure S25.** Life cycle emissions from eVTOLs and on-road vehicles (battery specific energy: 450 Wh/kg, electricity emission factor: 0.25 kg CO_2_/kWh, battery life: base case).


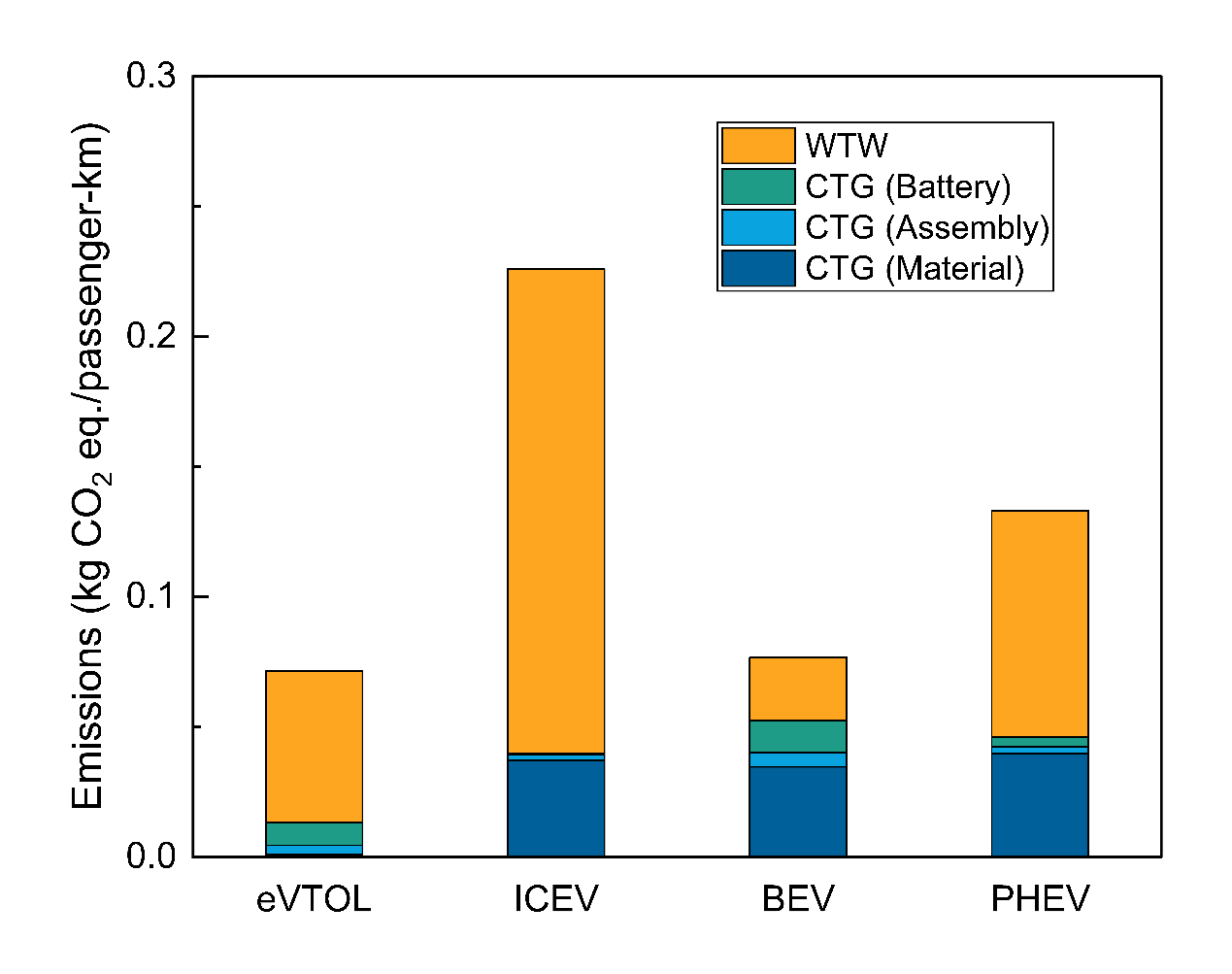


**Figure S26.** Life cycle emissions from eVTOLs and on-road vehicles (battery specific energy: 500 Wh/kg, electricity emission factor: 0.25 kg CO_2_/kWh, battery life: base case).


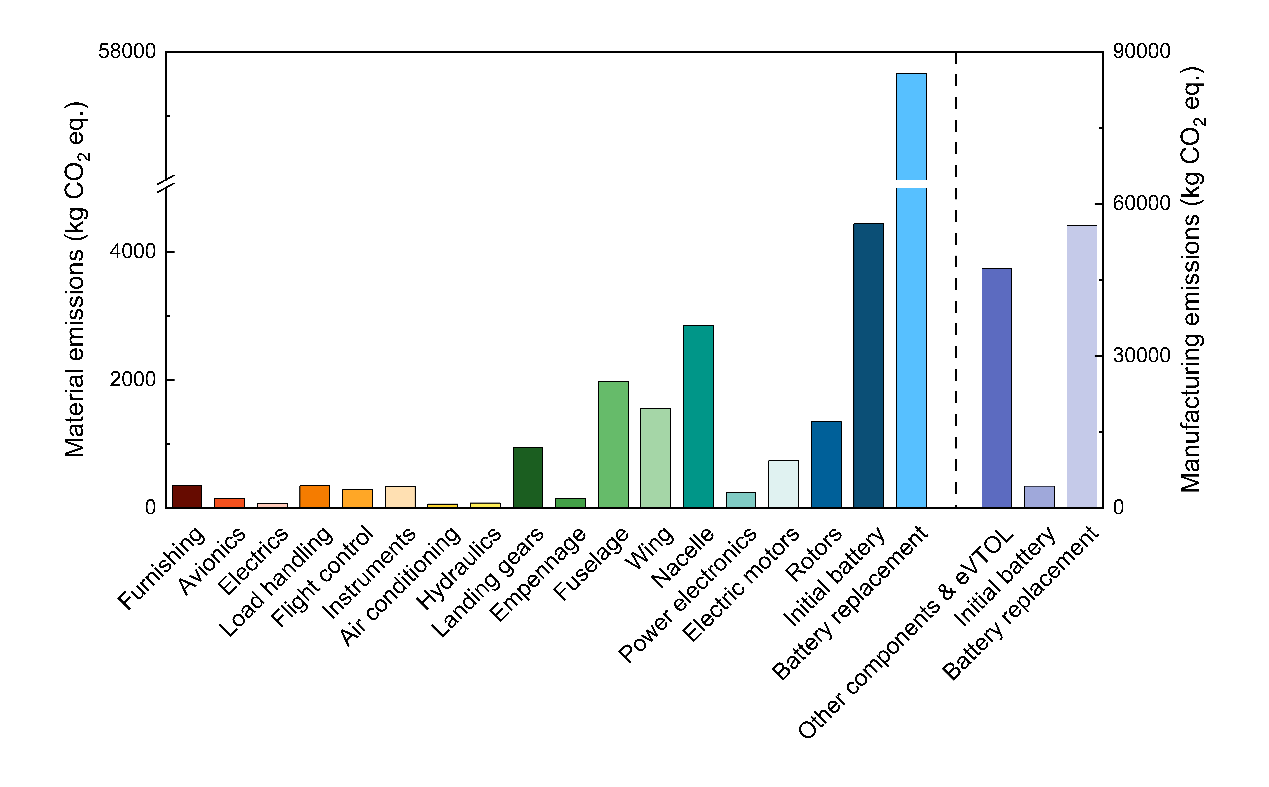


**Figure S27.** CTG emissions from eVTOLs (battery specific energy: base case, electricity emission factor: base case, battery life: 3000 cycles).


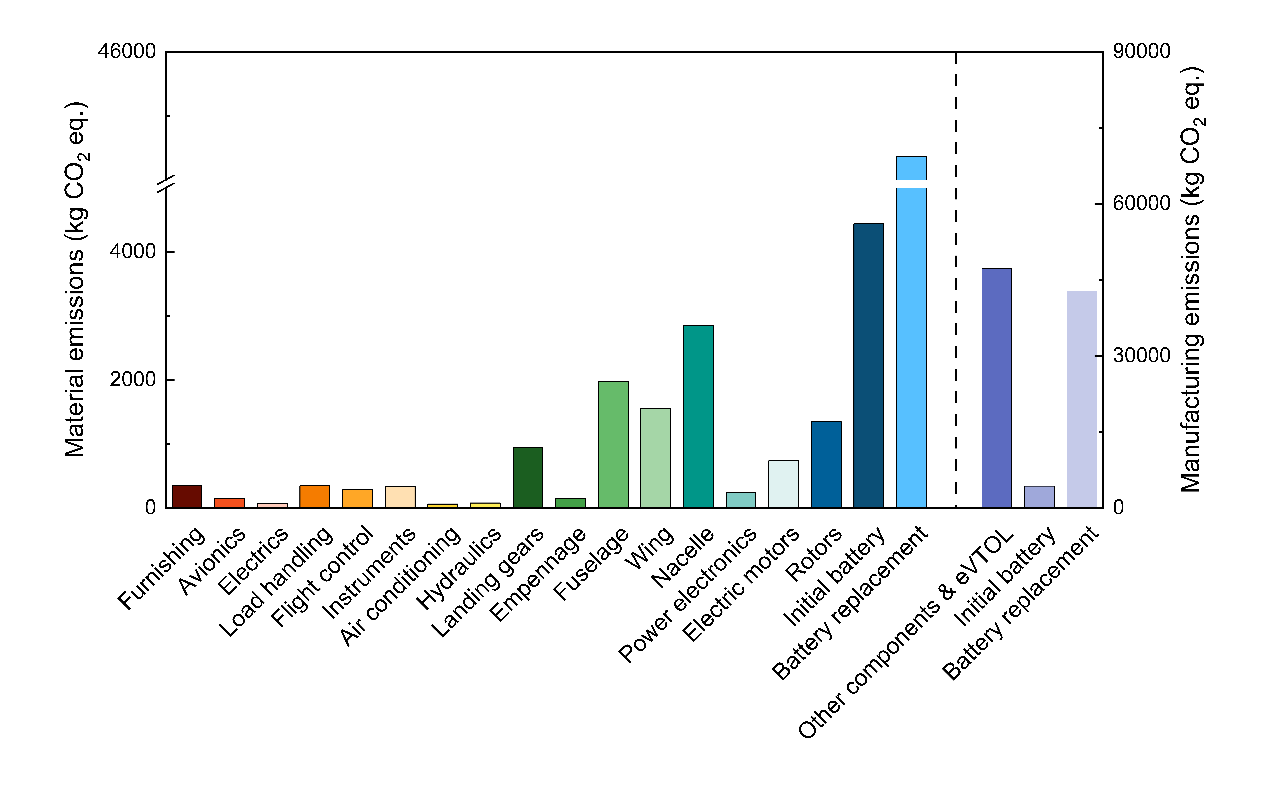


**Figure S28.** CTG emissions from eVTOLs (battery specific energy: base case, electricity emission factor: base case, battery life: 4000 cycles).


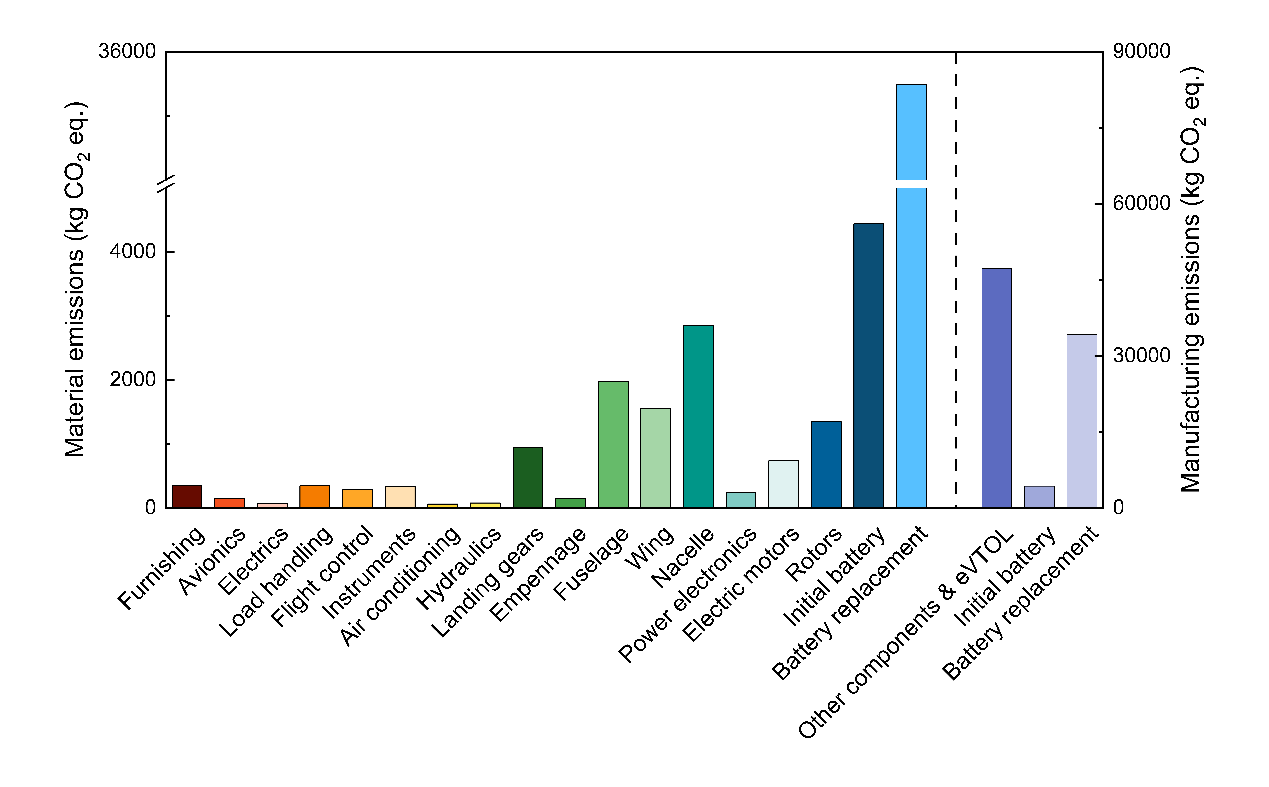


**Figure S29.** CTG emissions from eVTOLs (battery specific energy: base case, electricity emission factor: base case, battery life: 5000 cycles).


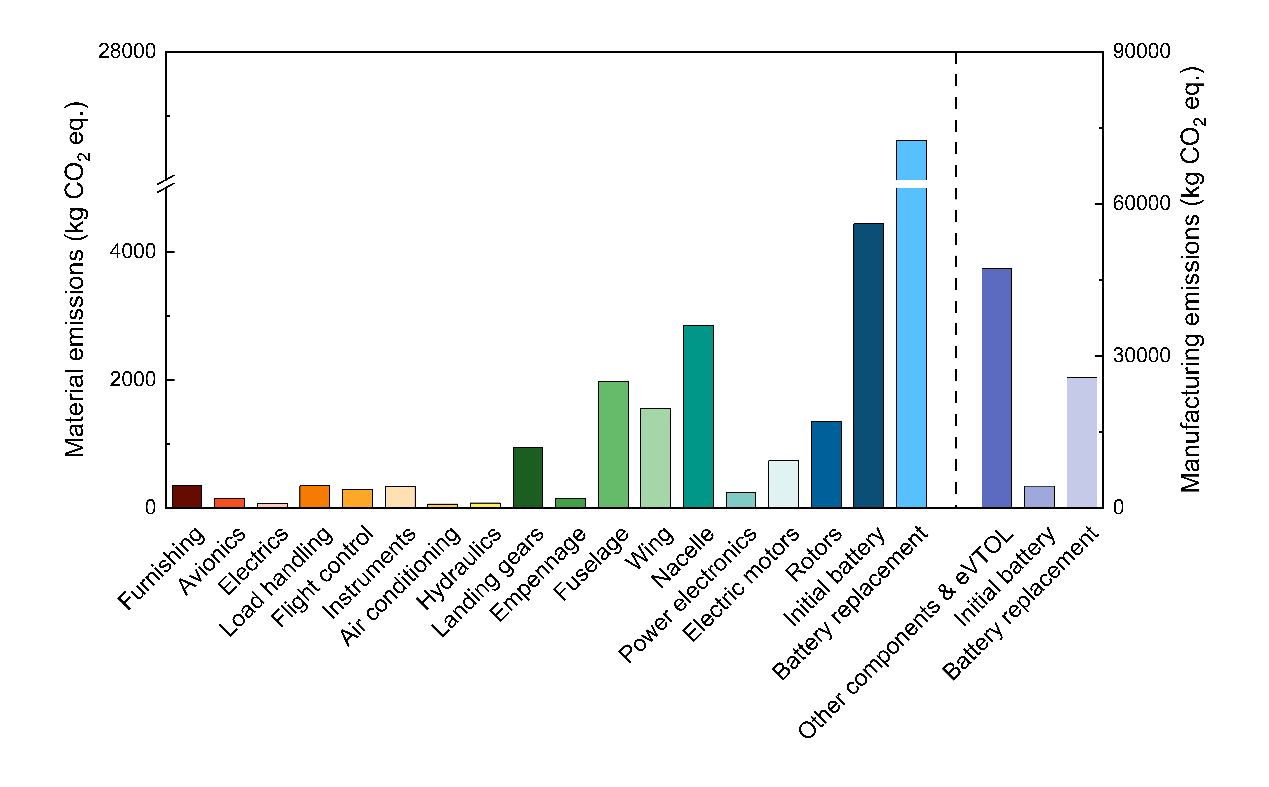


**Figure S30.** CTG emissions from eVTOLs (battery specific energy: base case, electricity emission factor: base case, battery life: 6000 cycles).


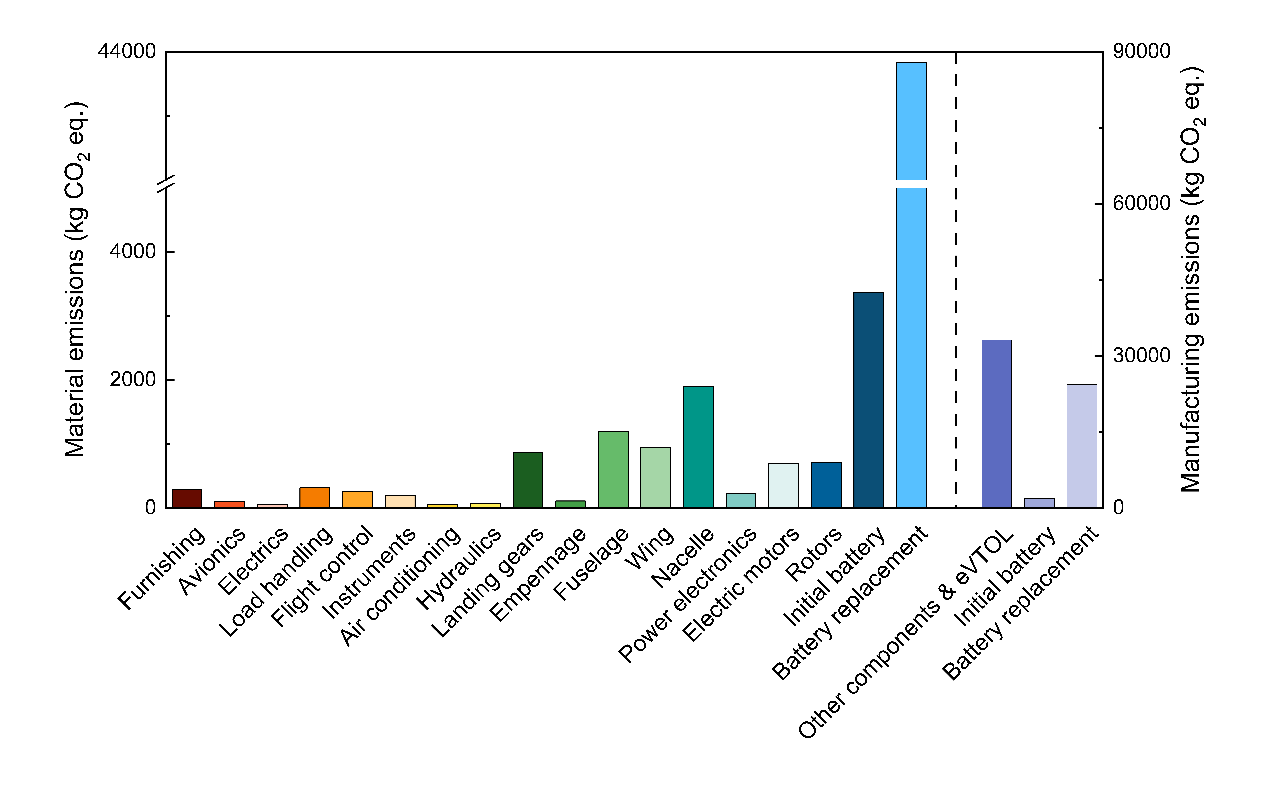


**Figure S31.** CTG emissions from eVTOLs (battery specific energy: base case, electricity emission factor: 0.25 kg CO_2_/kWh, battery life: 3000 cycles).


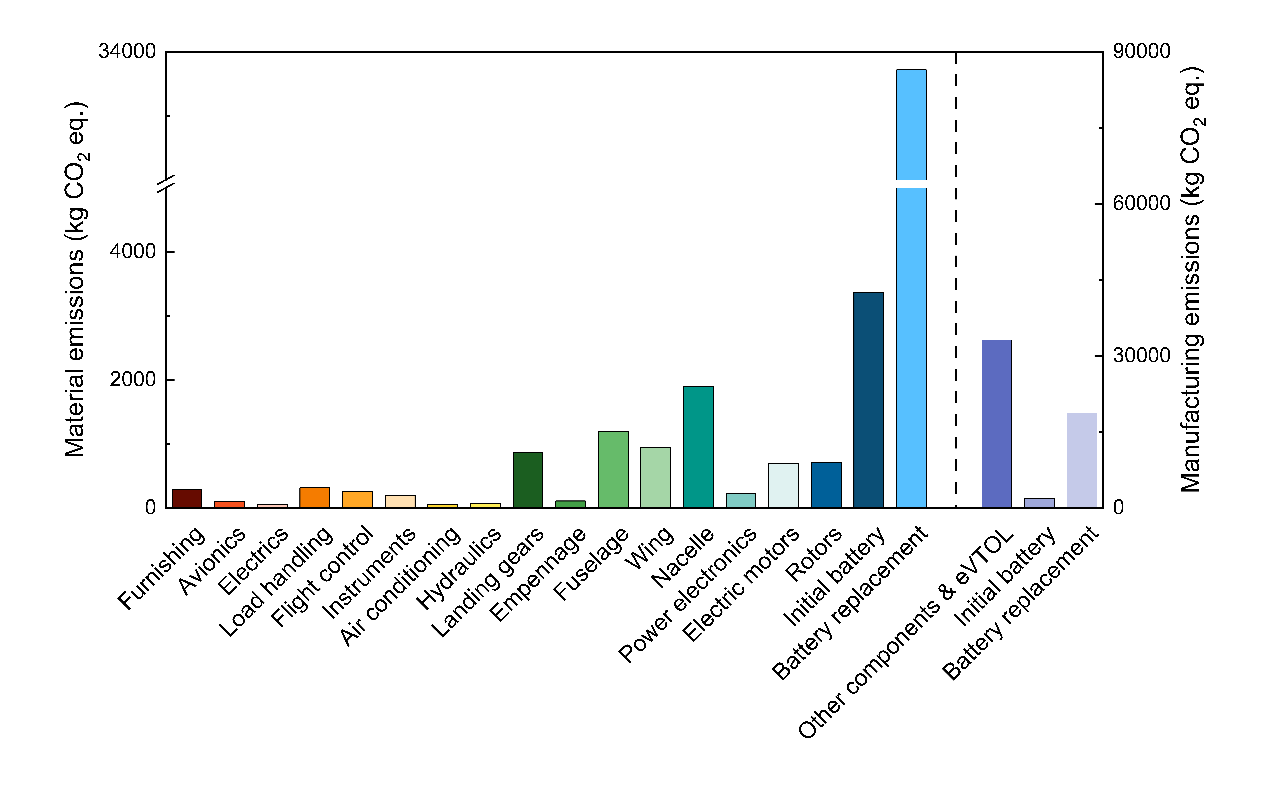


**Figure S32.** CTG emissions from eVTOLs (battery specific energy: base case, electricity emission factor: 0.25 kg CO_2_/kWh, battery life: 4000 cycles).


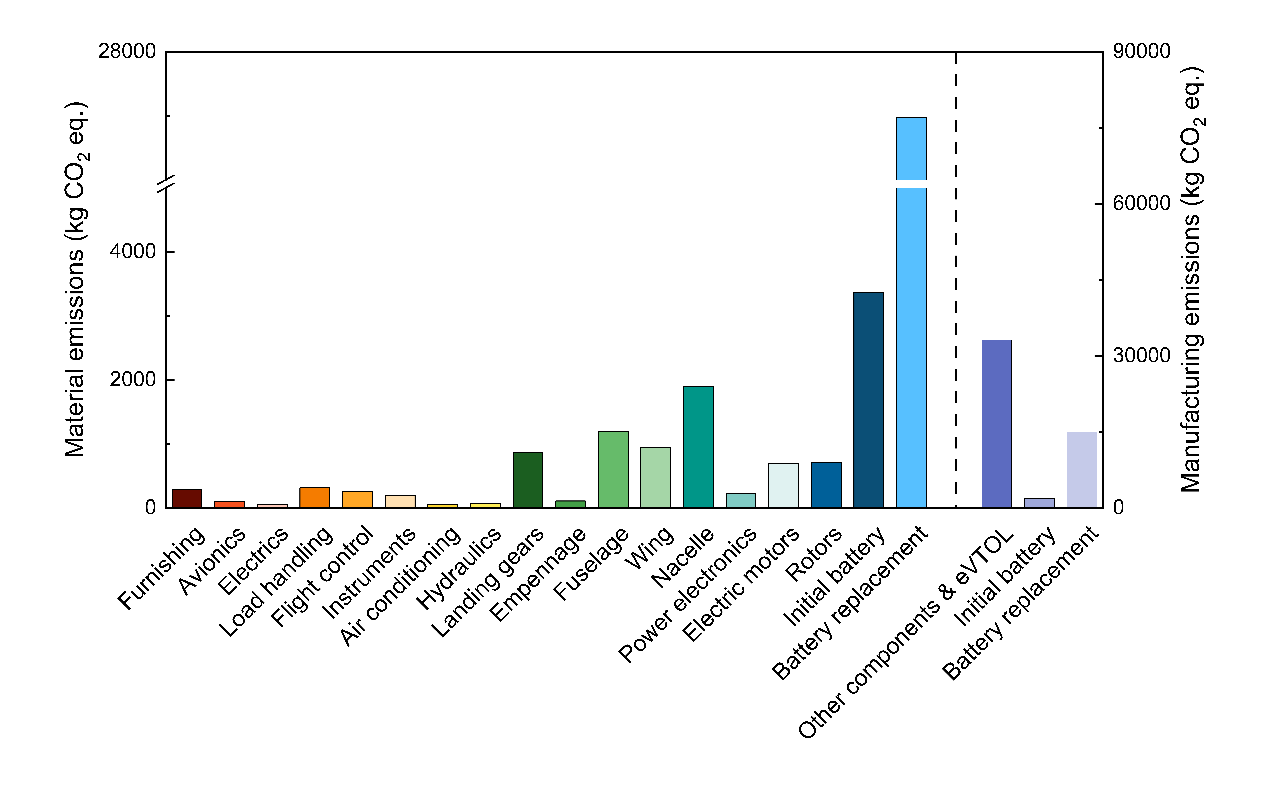


**Figure S33.** CTG emissions from eVTOLs (battery specific energy: base case, electricity emission factor: 0.25 kg CO_2_/kWh, battery life: 5000 cycles).


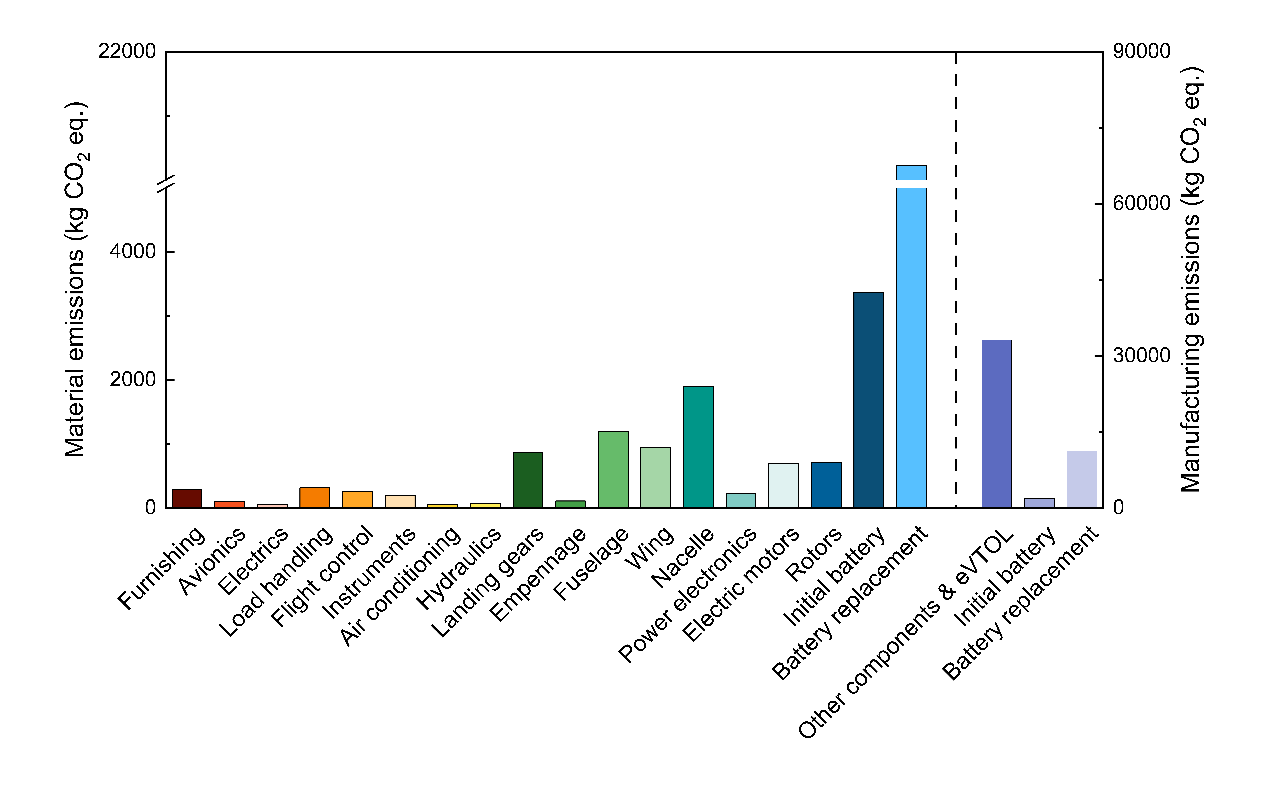


**Figure S34.** CTG emissions from eVTOLs (battery specific energy: base case, electricity emission factor: 0.25 kg CO_2_/kWh, battery life: 6000 cycles).


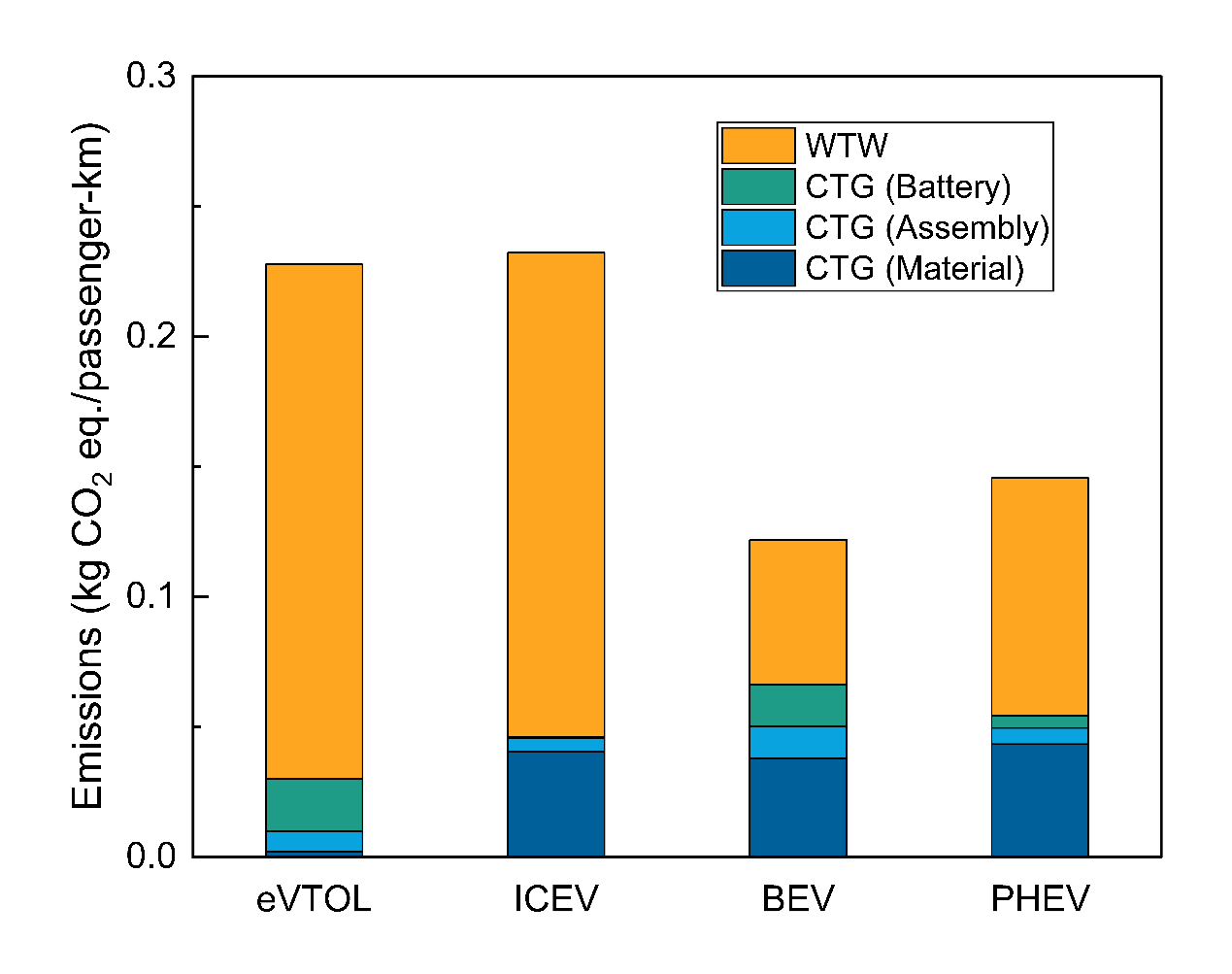


**Figure S35.** Life cycle emissions from eVTOLs and on-road vehicles (battery specific energy: base case, electricity emission factor: 0.57 kg CO_2_/kWh, battery life: 3000 cycles).


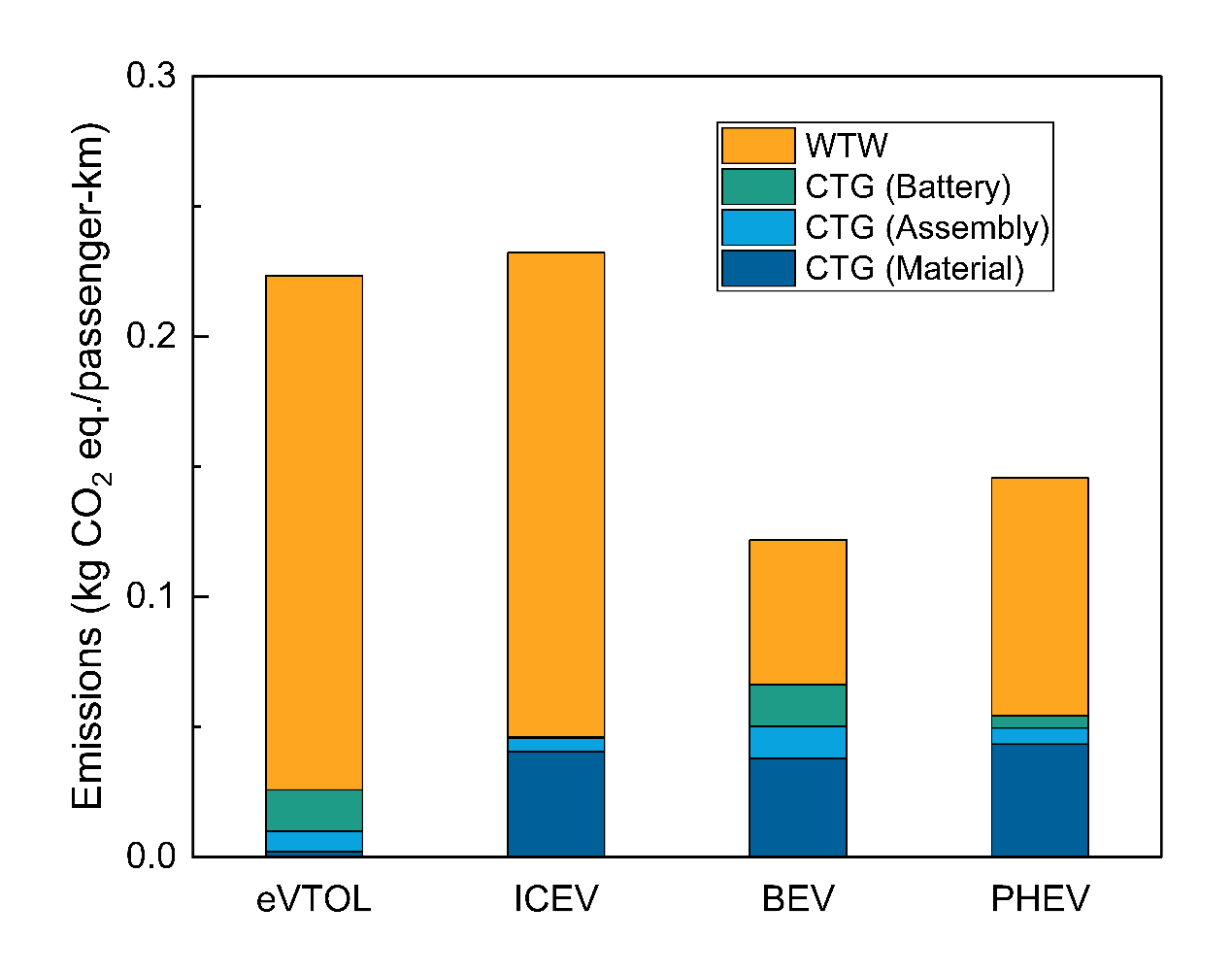


**Figure S36.** Life cycle emissions from eVTOLs and on-road vehicles (battery specific energy: base case, electricity emission factor: 0.57 kg CO_2_/kWh, battery life: 4000 cycles).


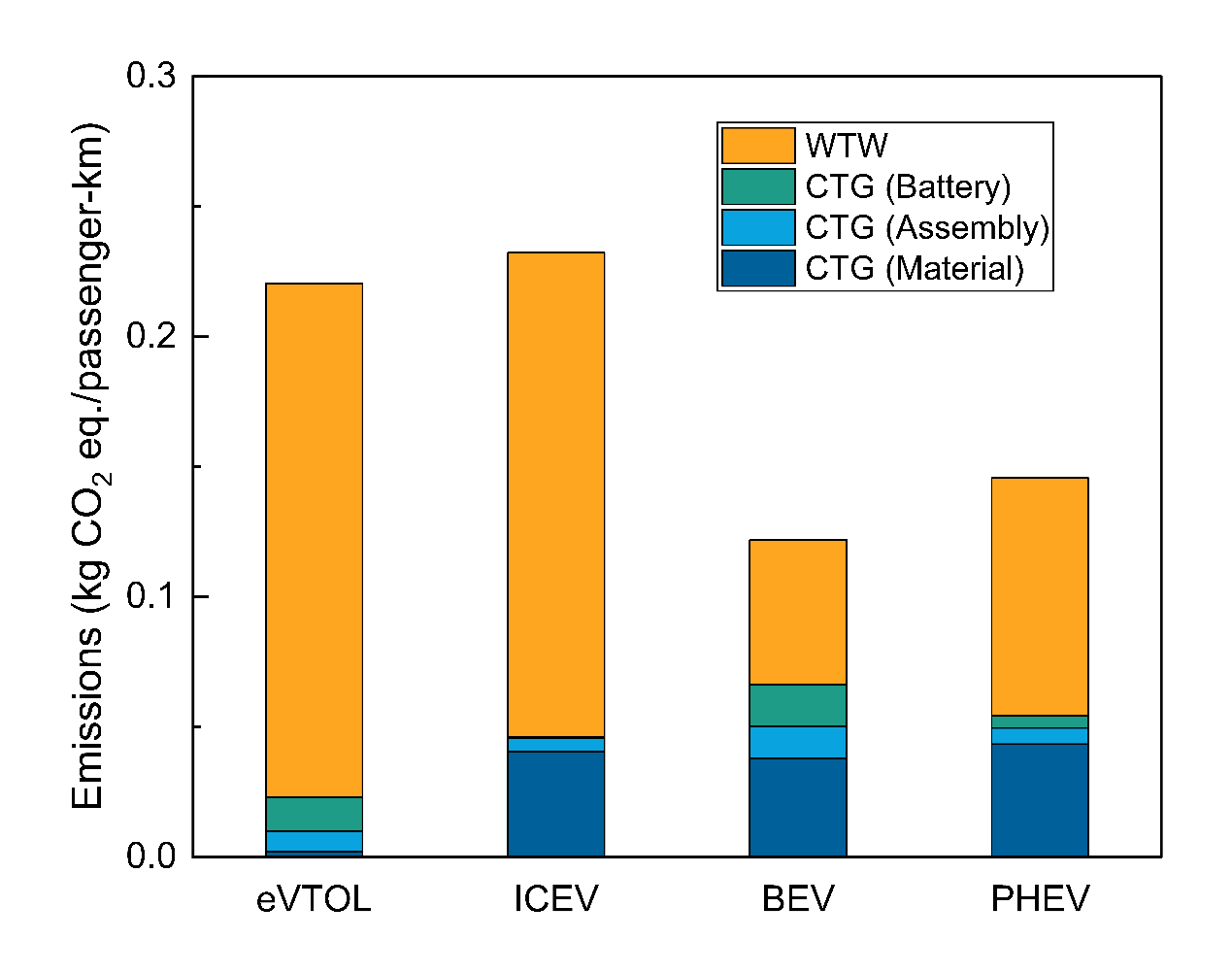


**Figure S37.** Life cycle emissions from eVTOLs and on-road vehicles (battery specific energy: base case, electricity emission factor: 0.57 kg CO_2_/kWh, battery life: 5000 cycles).


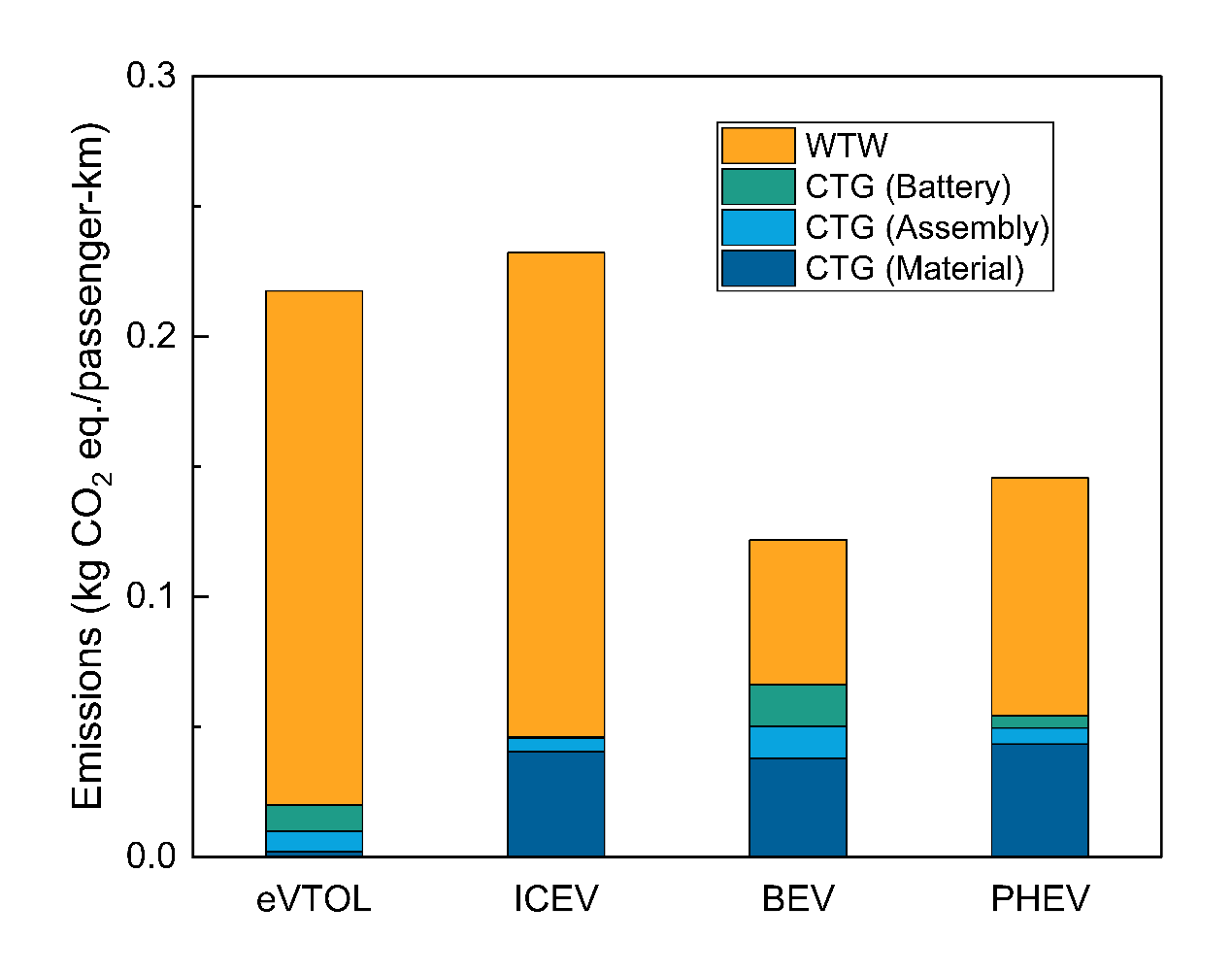


**Figure S38.** Life cycle emissions from eVTOLs and on-road vehicles (battery specific energy: base case, electricity emission factor: 0.57 kg CO_2_/kWh, battery life: 6000 cycles).


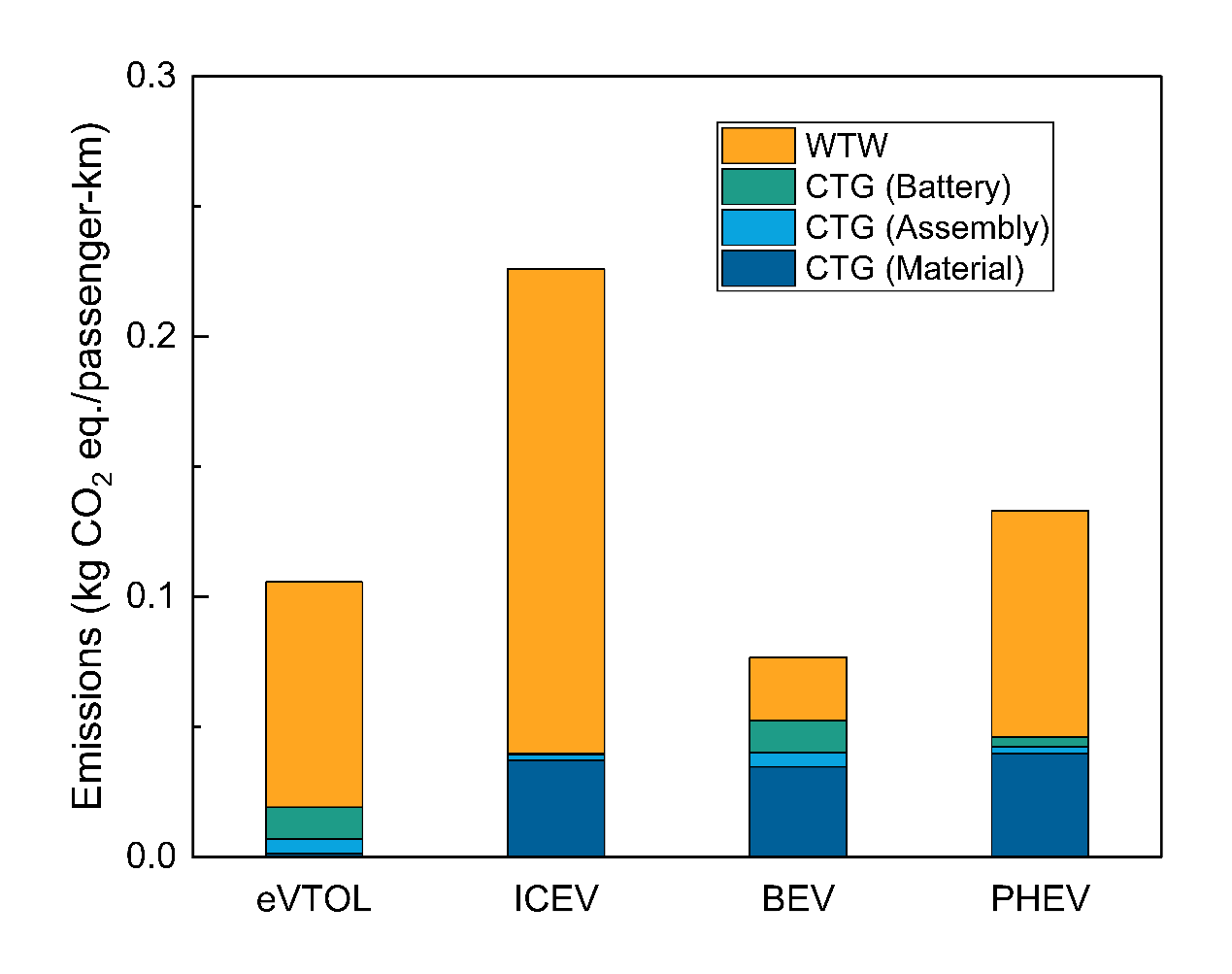


**Figure S39.** Life cycle emissions from eVTOLs and on-road vehicles (battery specific energy: base case, electricity emission factor: 0.25 kg CO_2_/kWh, battery life: 3000 cycles).


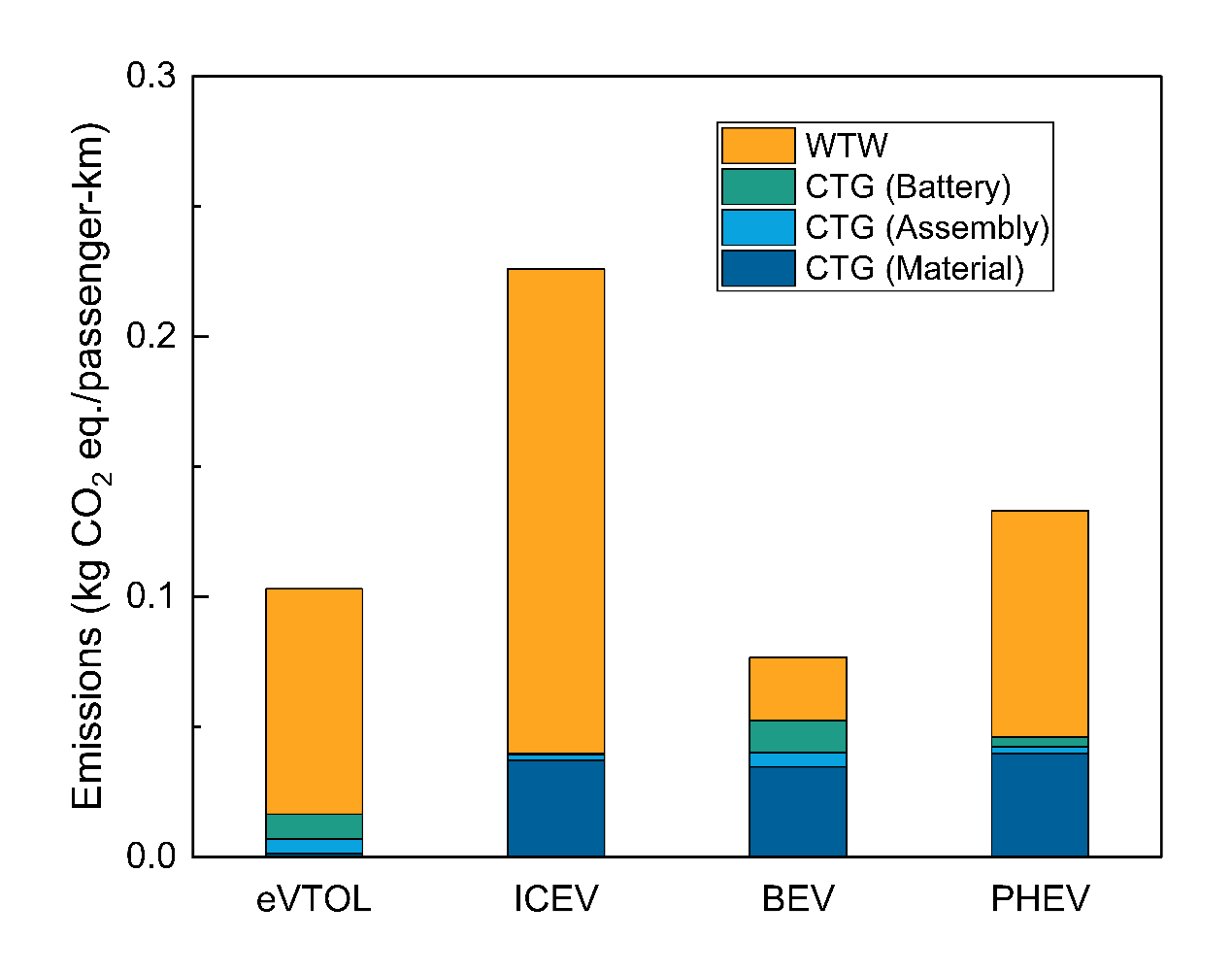


**Figure S40.** Life cycle emissions from eVTOLs and on-road vehicles (battery specific energy: base case, electricity emission factor: 0.25 kg CO_2_/kWh, battery life: 4000 cycles).


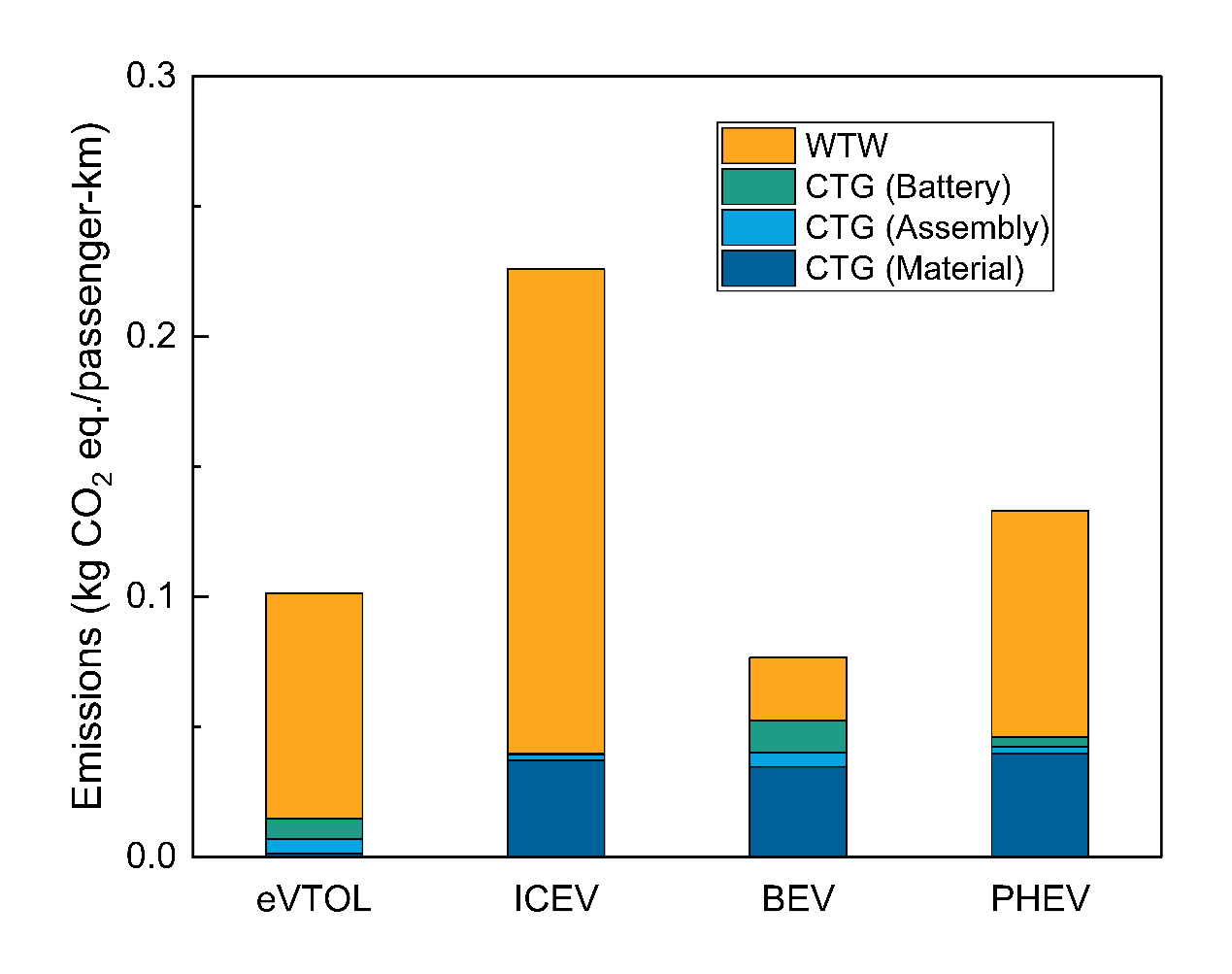


**Figure S41.** Life cycle emissions from eVTOLs and on-road vehicles (battery specific energy: base case, electricity emission factor: 0.25 kg CO_2_/kWh, battery life: 5000 cycles).


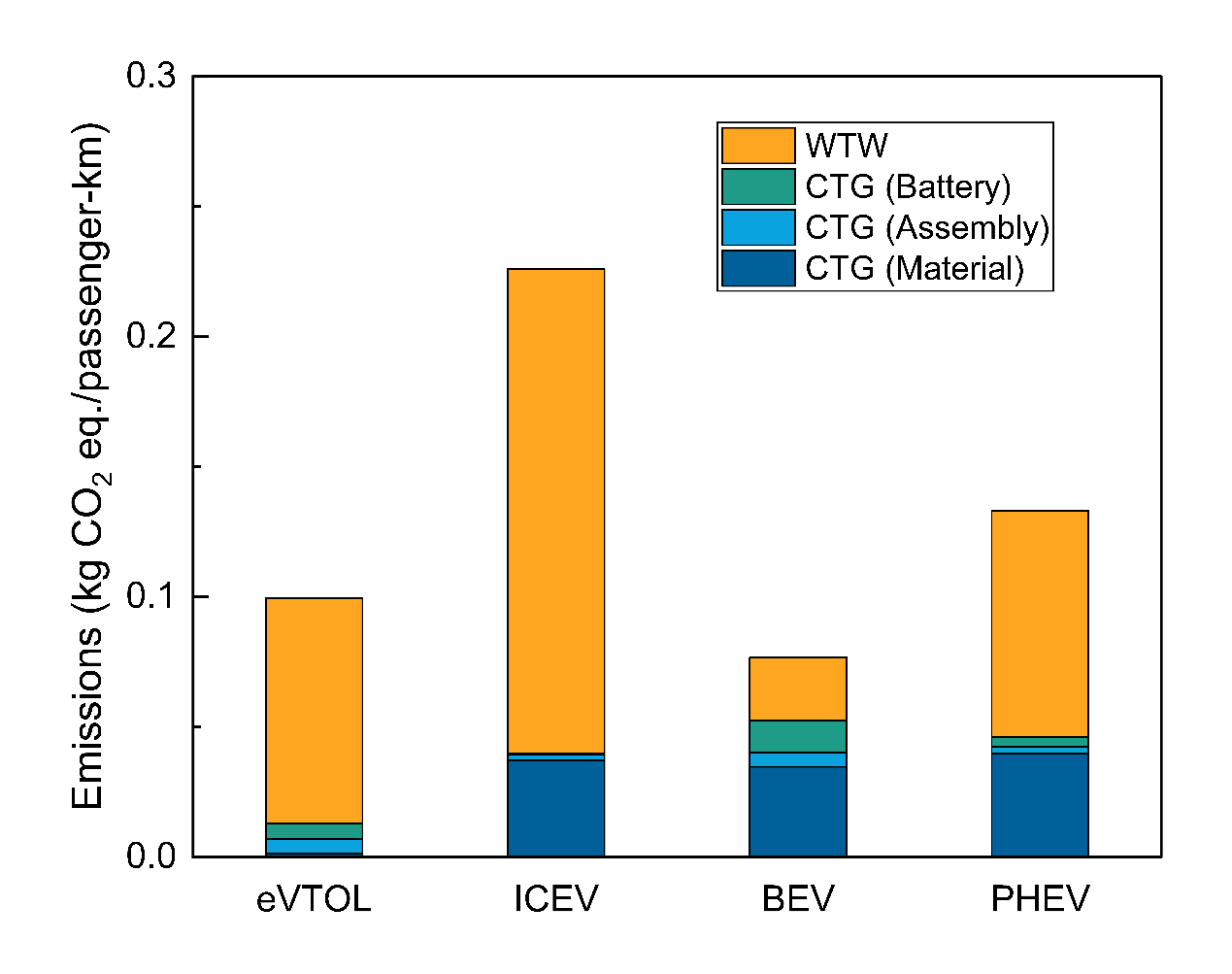


**Figure S42.** Life cycle emissions from eVTOLs and on-road vehicles (battery specific energy: base case, electricity emission factor: 0.25 kg CO_2_/kWh, battery life: 6000 cycles).


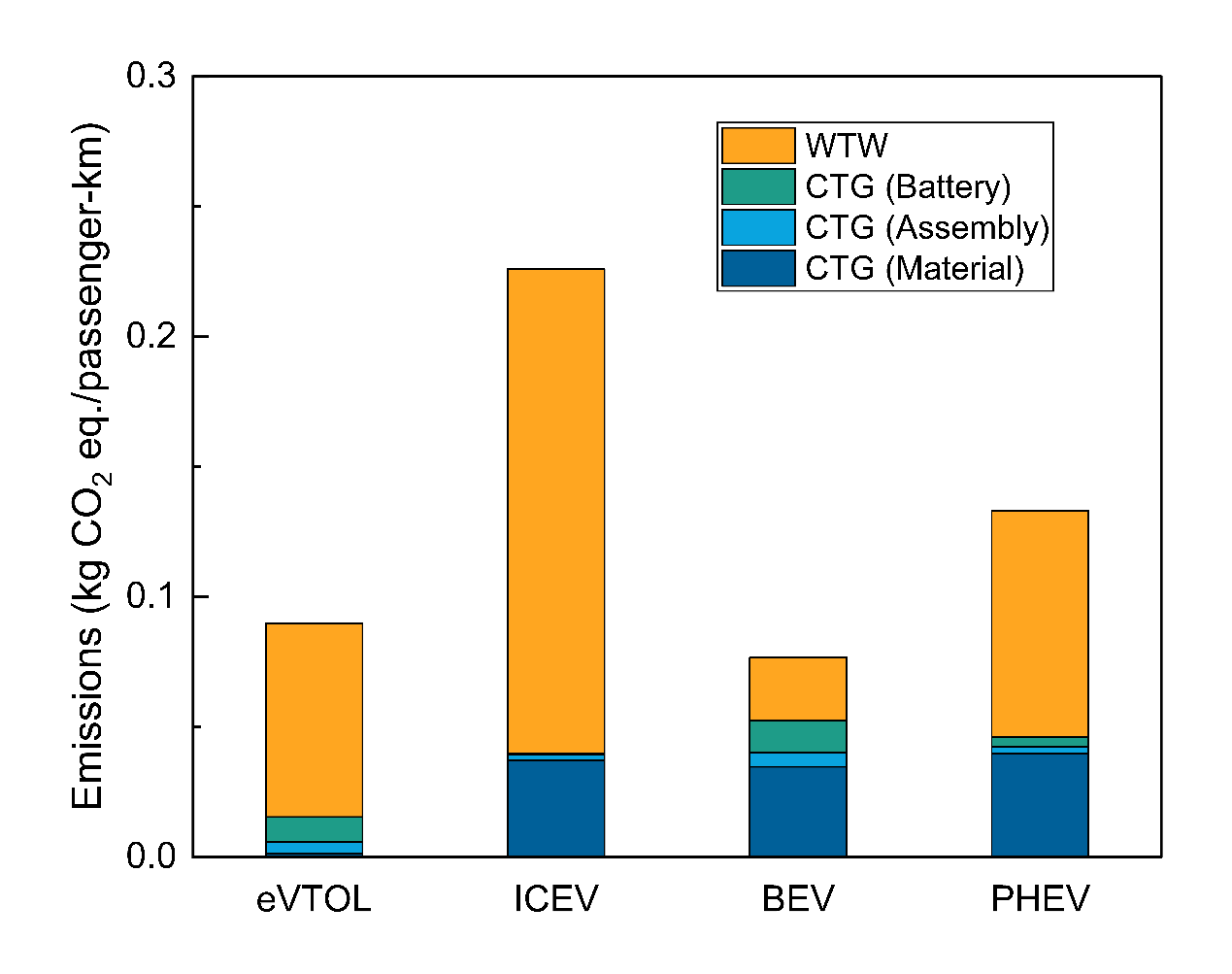


**Figure S43.** Life cycle emissions from eVTOLs and on-road vehicles under AMT scenario (battery specific energy: 300 Wh/kg, electricity emission factor: 0.25 kg CO_2_/kWh, battery life: 3000 cycles). AMT: All Measures Taken scenario.


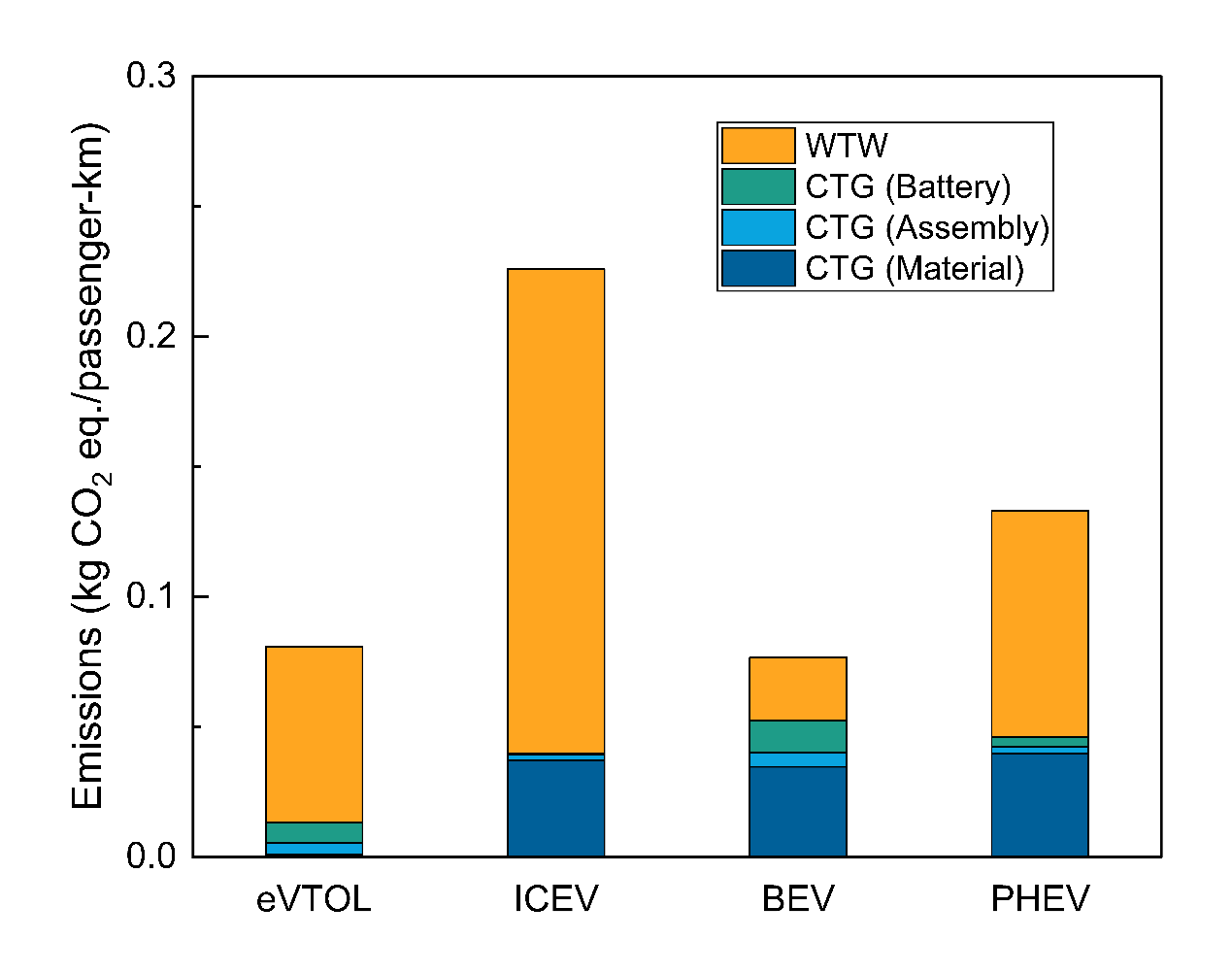


**Figure S44**. Life cycle emissions from eVTOLs and on-road vehicles under AMT scenario (battery specific energy: 350 Wh/kg, electricity emission factor: 0.25 kg CO_2_/kWh, battery life: 3000 cycles). AMT: All Measures Taken scenario.


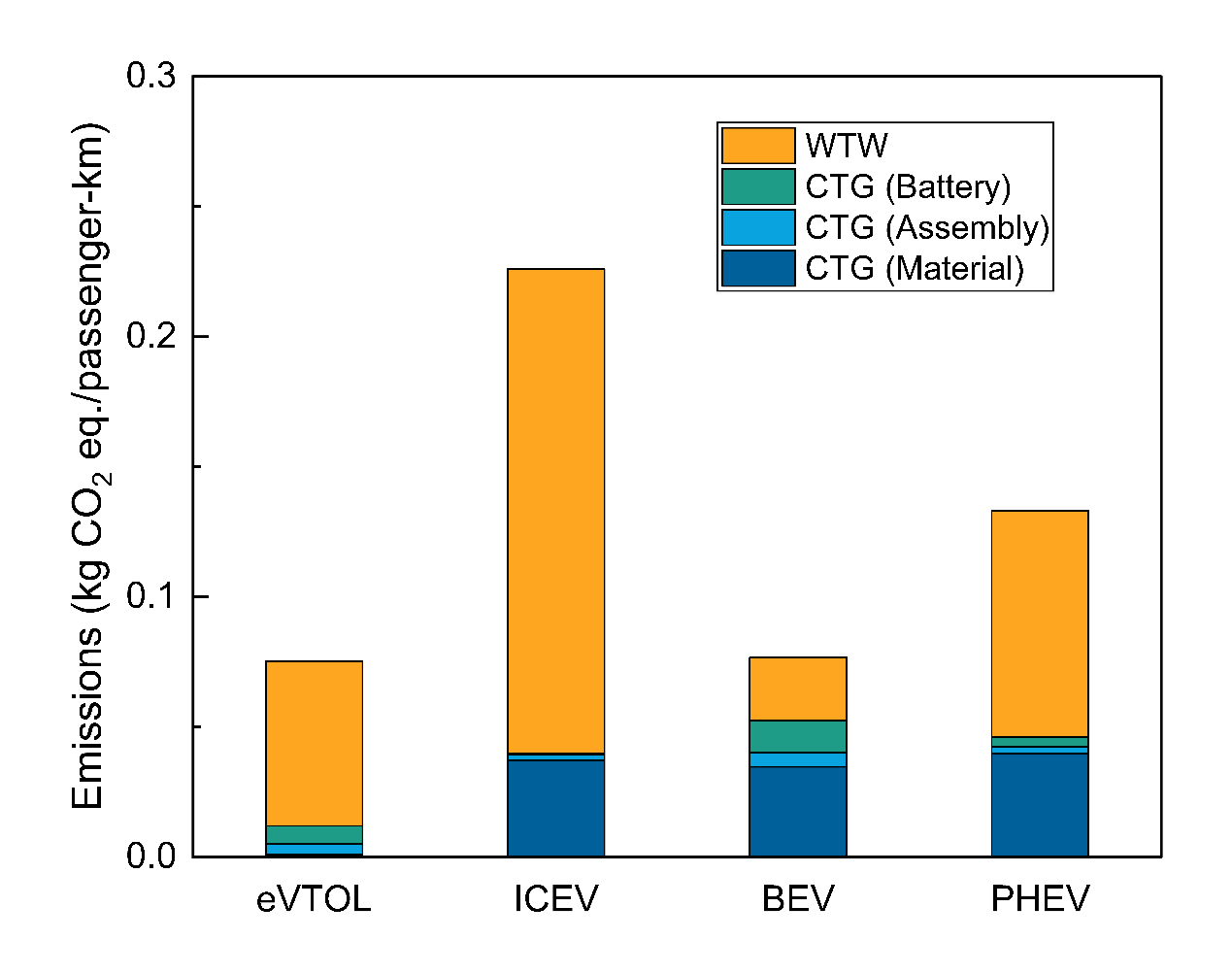


**Figure S45.** Life cycle emissions from eVTOLs and on-road vehicles under AMT scenario (battery specific energy: 400 Wh/kg, electricity emission factor: 0.25 kg CO_2_/kWh, battery life: 3000 cycles). AMT: All Measures Taken scenario.


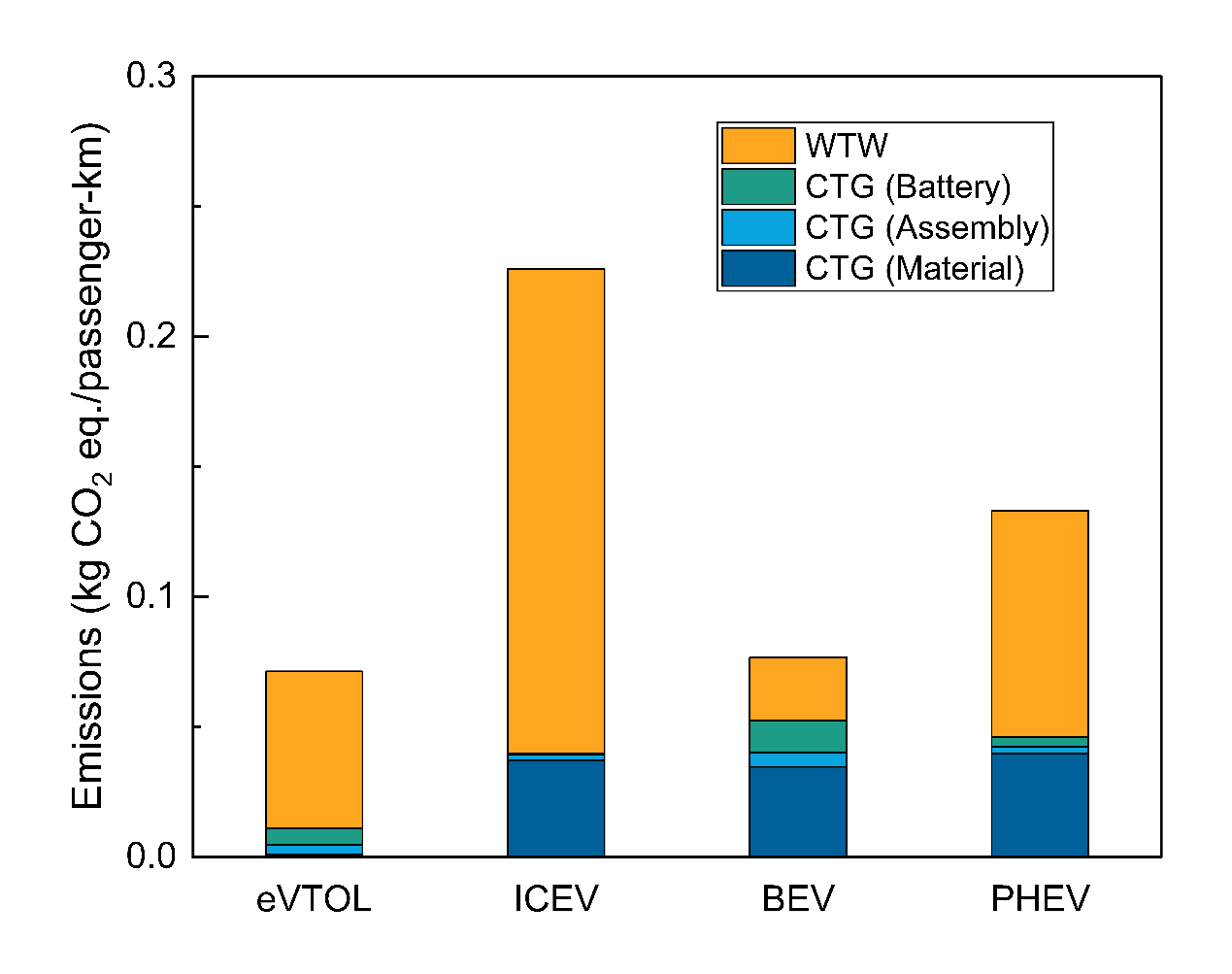


**Figure S46**. Life cycle emissions from eVTOLs and on-road vehicles under AMT scenario (battery specific energy: 450 Wh/kg, electricity emission factor: 0.25 kg CO_2_/kWh, battery life: 3000 cycles). AMT: All Measures Taken scenario.


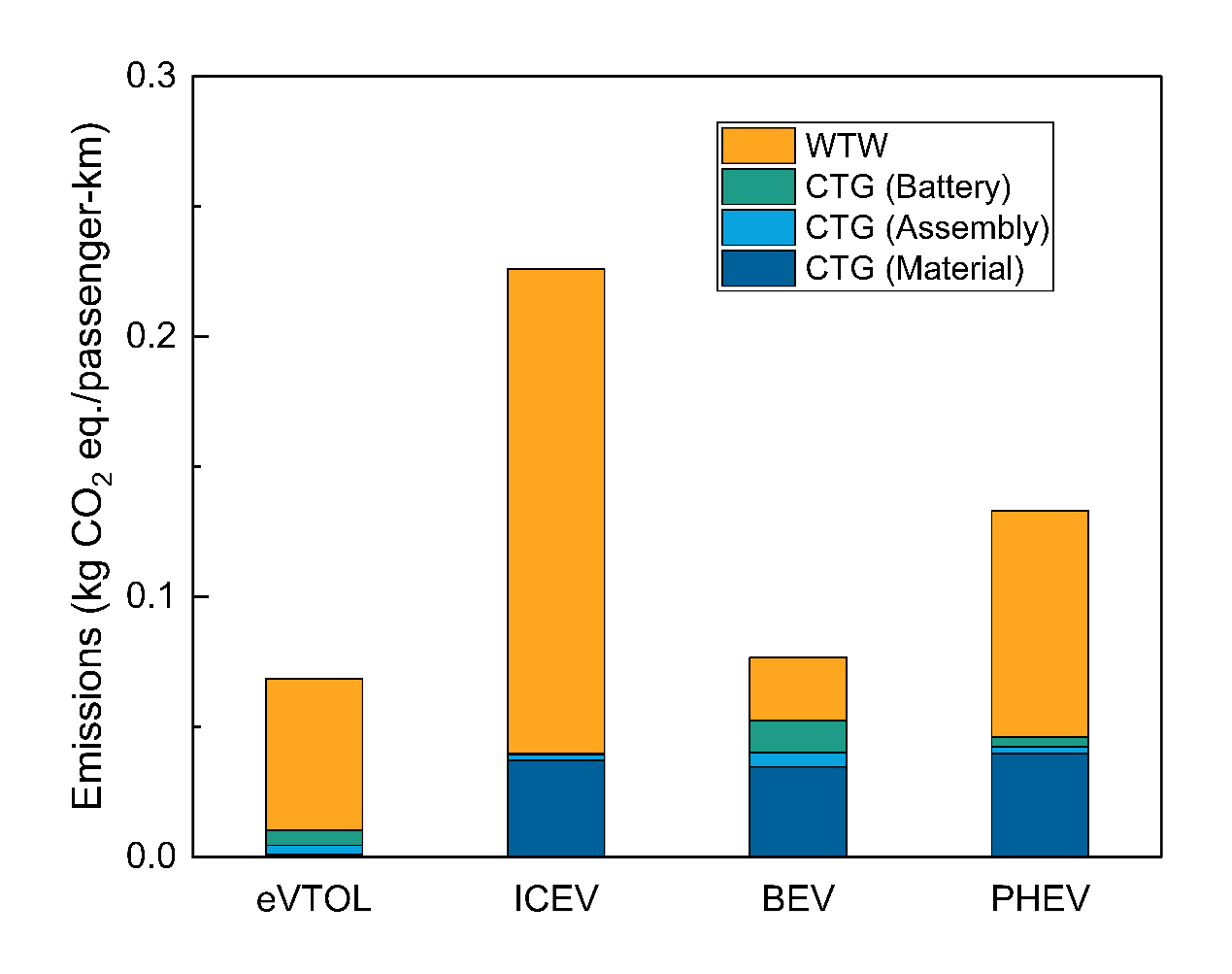


**Figure S47.** Life cycle emissions from eVTOLs and on-road vehicles under AMT scenario (battery specific energy: 500 Wh/kg, electricity emission factor: 0.25 kg CO_2_/kWh, battery life: 3000 cycles). AMT: All Measures Taken scenario.


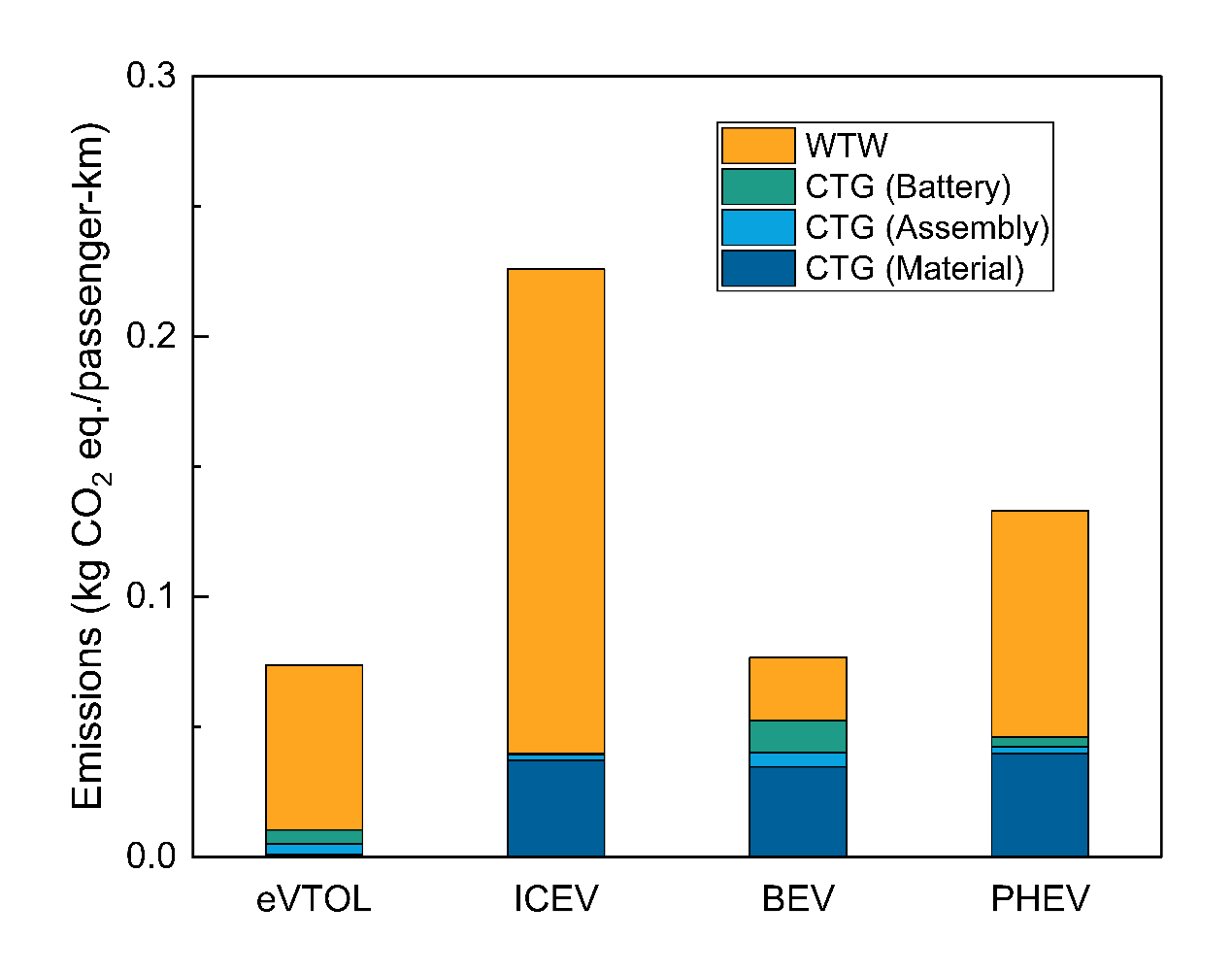


**Figure S48.** Life cycle emissions from eVTOLs and on-road vehicles under AMT scenario (battery specific energy: 400 Wh/kg, electricity emission factor: 0.25 kg CO_2_/kWh, battery life: 4000 cycles). AMT: All Measures Taken scenario.


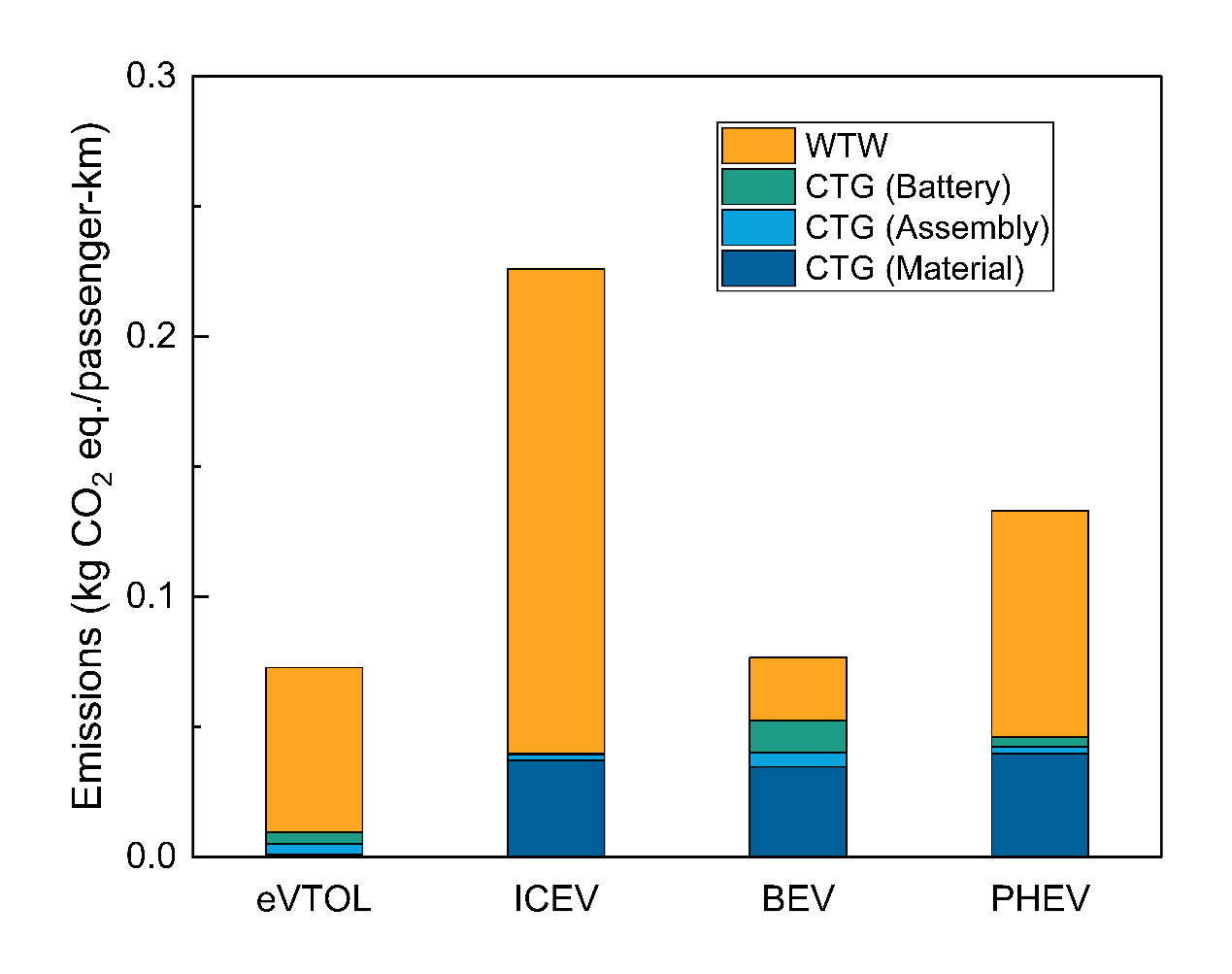


**Figure S49**. Life cycle emissions from eVTOLs and on-road vehicles under AMT scenario (battery specific energy: 400 Wh/kg, electricity emission factor: 0.25 kg CO_2_/kWh, battery life: 5000 cycles). AMT: All Measures Taken scenario.


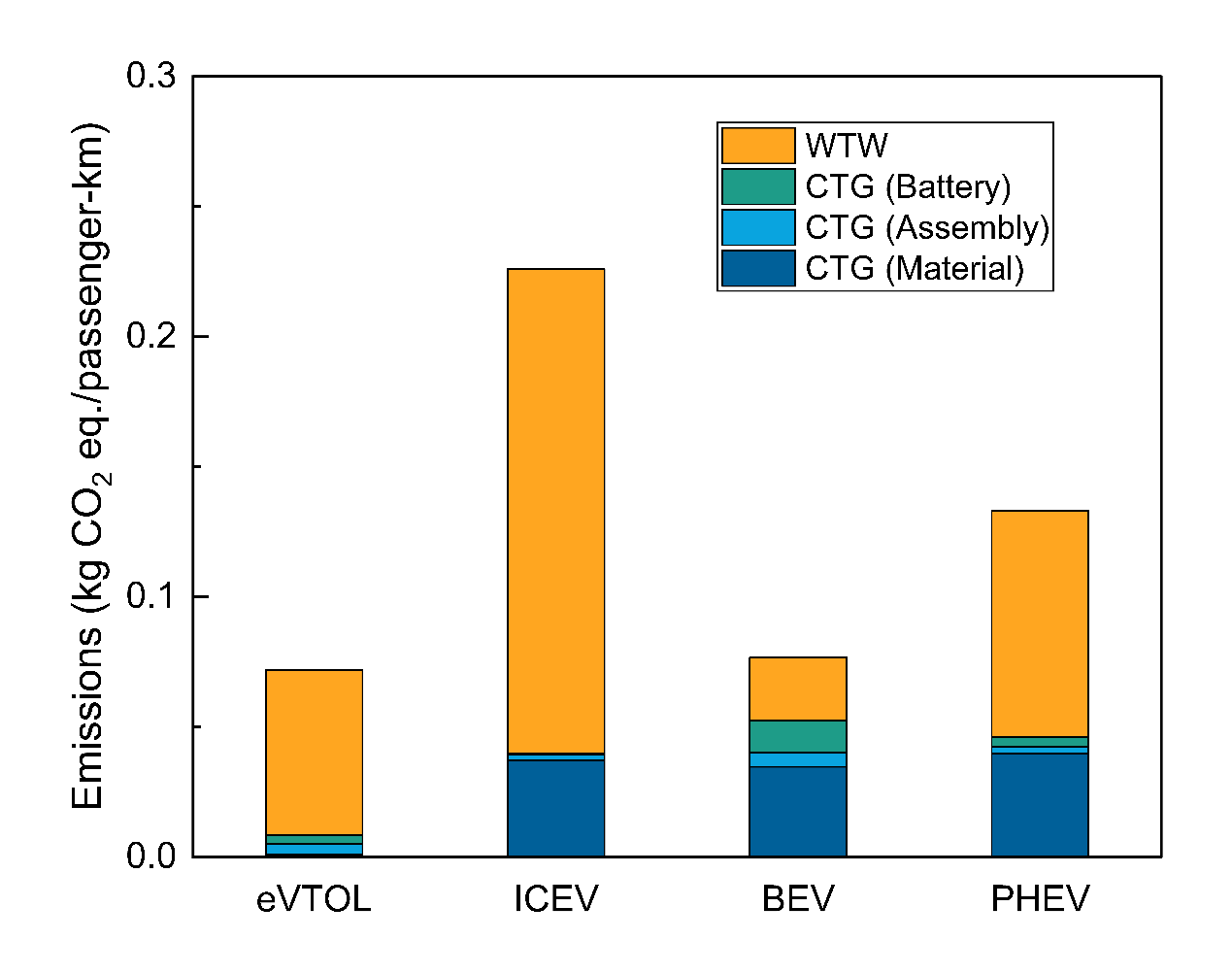


**Figure S50.** Life cycle emissions from eVTOLs and on-road vehicles under AMT scenario (battery specific energy: 400 Wh/kg, electricity emission factor: 0.25 kg CO_2_/kWh, battery life: 6000 cycles). AMT: All Measures Taken scenario.


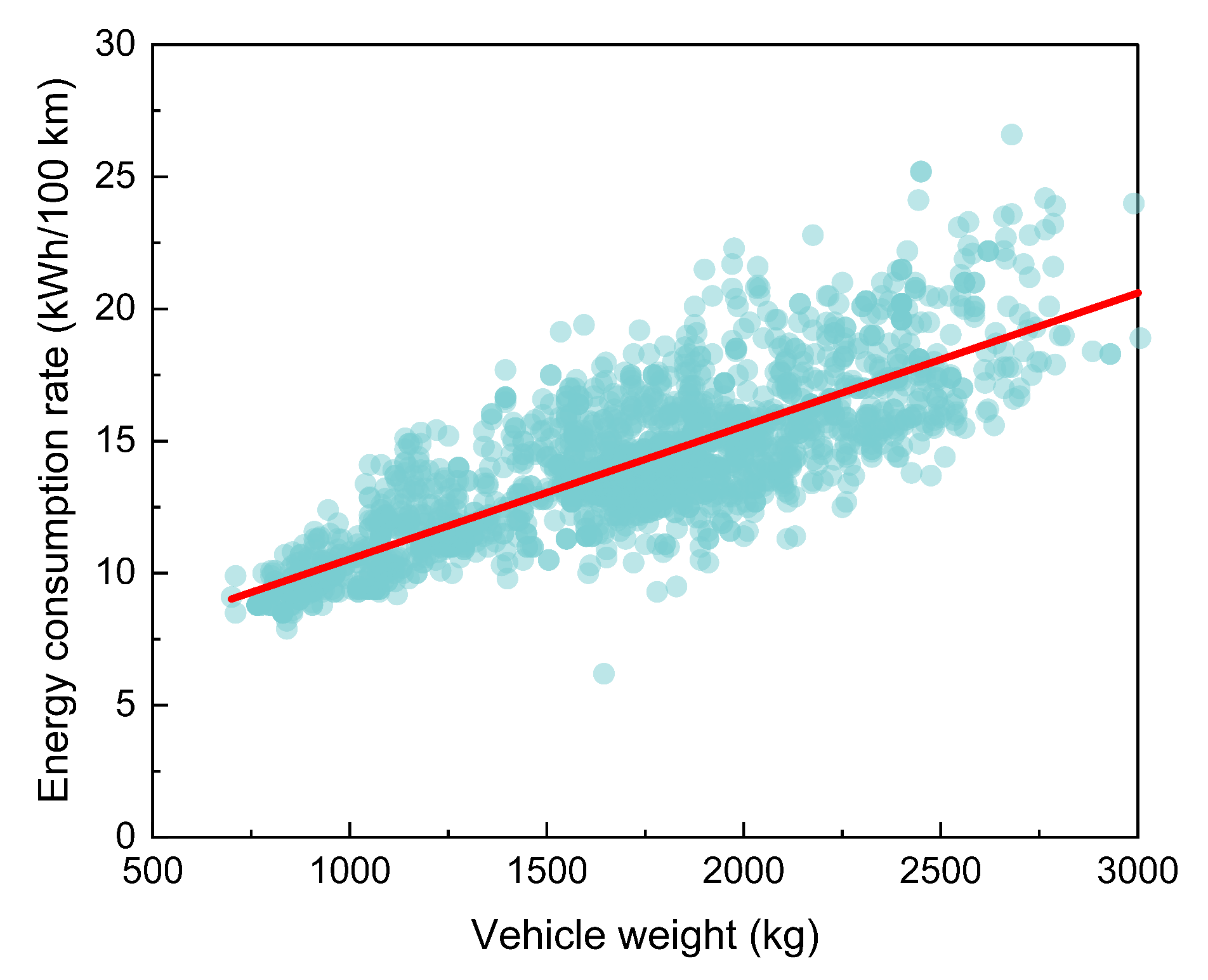


**Figure S51.** Relationship between BEV weight and energy consumption rate. BEV: Battery Electric Vehicle.

**Supplemental Tables**

**Table S1.** Parameters for flying process model

| Parameter | | | Notation | Unit | Value | References |
| --- | --- | --- | --- | --- | --- | --- |
| Disk loading | | | $\delta$ | N/m^2^ | 450 | ^1^ |
| Air density | | | $\rho$ | kg/m^3^ | 1.29 |  |
| Cruising altitude | | | $h$ | m | 305 | ^2^ |
| Cruising speed | | | $V_{cruise}$ | km/h | 200 | ^2-4^ |
| Angle of flight | | | $\alpha$ | degree | 3 | ^3^ |
| L/D while cruising | | | $L/D_{cruise}$ | - | 14 | ^5^ |
| L/D while climbing | | | $L/D_{climb}$ | - | 10.5 | ^5^ |
| Rate of climb | | | $ROC$ | m/s | 2.54 | ^3^ |
| Rate of descent | | | $ROD$ | m/s | 2.54 | ^3^ |
| Battery | Specific energy | | $\rho_{Benergy}$ | Wh/kg | 250 | ^6^ |
|  | Cycle life | | - | cycles | 2000 |  |
|  | Charging efficiency | | - | - | 90% | ^6^ |
| Electric motor | | Power density | $\rho_{EMpower}$ | kW/kg | 4 | ^5^ |
|  |  | Energy efficiency | - | - | 92% | ^6^ |
| Transmission efficiency | | | $\eta_{T}$ | - | 98% | ^6^ |
| Additional cruising time | | | $t_{add}$ | min | 15 | ^2^ |
| Hovering or landing time | | | $t_{hover}$ | s | 30 | ^2^ |

**Table S2.** Parameters for component sizing model

| Parameter | Notation | Unit | Value | References |
| --- | --- | --- | --- | --- |
| Ratio of all other components mass to takeoff mass | $R_{AOC-TO}$ | - | 0.50 | ^5,7,8^ |
| Ratio of nacelle mass to battery mass | $R_{NC-B}$ | - | 0.17 | ^9,10^ |
| Ratio of furnishing mass to all other components mass | $R_{FR-AOC}$ | - | 0.14 |  |
| Ratio of avionics mass to all other components mass | $R_{AV-AOC}$ |  | 0.09 |  |
| Ratio of electrics mass to all other components mass | $R_{ET-AOC}$ |  | 0.09 |  |
| Ratio of load handling mass to all other components mass | $R_{LH-AOC}$ |  | 0.05 |  |
| Ratio of flight control mass to all other components mass | $R_{FC-AOC}$ |  | 0.04 |  |
| Ratio of instrument mass to all other components mass | $R_{IN-AOC}$ |  | 0.03 |  |
| Ratio of air conditioning mass to all other components mass | $R_{AC-AOC}$ |  | 0.01 |  |
| Ratio of hydraulics mass to all other components mass | $R_{HY-AOC}$ |  | 0.01 |  |
| Ratio of landing gear mass to all other components mass | $R_{LG-AOC}$ |  | 0.08 |  |
| Ratio of power electronics mass to all other components mass | $R_{PE-AOC}$ |  | 0.03 |  |
| Ratio of fuselage mass to all other components mass | $R_{FL-AOC}$ |  | 0.17 |  |
| Ratio of wing mass to all other components mass | $R_{WG-AOC}$ |  | 0.13 |  |
| Ratio of rotor mass to all other components mass | $R_{RT-AOC}$ |  | 0.13 |  |
| Ratio of empennage mass to all other components mass | $R_{EP-AOC}$ |  | 0.01 |  |

* All other components represent components excluding battery, electric motor and nacelle, which is used for battery protection.

**Table S3.** Parameters for operation characterizing model

| Parameter | Notation | Unit | Value | References |
| --- | --- | --- | --- | --- |
| Yearly flight time | $YT$ | h | 2,000 | ^6^ |
| VTOL life | $VL$ | yr | 10 | ^6^ |
| Number of seats | $N_{seat}$ | - | 4 | assumed |
| Range | $R$ | km | 100 | assumed |
| Load factor | $LF$ | - | 50% | ^6^ |

**Table S4**. Bill of materials data for eVTOL components^11,12^

|  | Furnishing | Avionics | Electrics | Load handling | Flight control | Instruments | Air conditioning | Hydraulics | Landing gear |
| --- | --- | --- | --- | --- | --- | --- | --- | --- | --- |
| Aluminum |  |  |  |  |  | 0.741 | 0.120 |  | 0.048 |
| Steel |  | 0.027 |  | 0.670 | 0.670 | 0.015 | 0.460 | 0.175 | 0.884 |
| CFRP |  |  |  |  |  |  |  |  | 0.019 |
| Titanium |  |  |  |  |  |  |  |  | 0.048 |
| Copper |  | 0.305 |  | 0.330 | 0.330 |  | 0.190 |  |  |
| Plastics |  | 0.010 |  |  |  |  | 0.230 |  |  |
| Steel alloy |  |  |  |  |  |  |  | 0.238 |  |
| Coated steel |  |  |  |  |  |  |  | 0.070 |  |
| RIMFG |  |  |  |  |  |  |  | 0.018 |  |
| Cast steel |  |  |  |  |  |  |  | 0.158 |  |
| Al2024 |  |  |  |  |  |  |  | 0.254 |  |
| Synthetic rubber |  | 0.011 |  |  |  |  |  | 0.088 |  |
| Nylon 6 | 0.061 |  |  |  |  | 0.002 |  |  |  |
| Viscose | 0.363 |  |  |  |  |  |  |  |  |
| Polyurethane | 0.576 | 0.021 | 0.375 |  |  |  |  |  |  |
| Paper |  |  |  |  |  | 0.023 |  |  |  |
| Halocarbon |  |  |  |  |  | 0.027 |  |  |  |
| Silicone |  |  |  |  |  | 0.024 |  |  |  |
| PVC |  |  |  |  |  | 0.024 |  |  |  |
| Polyurethane coated nylon |  |  |  |  |  | 0.143 |  |  |  |
| Nickel |  | 0.002 |  |  |  |  |  |  |  |
| PP |  | 0.043 |  |  |  |  |  |  |  |
| Nylon |  | 0.001 |  |  |  |  |  |  |  |
| Cast aluminum |  | 0.529 |  |  |  |  |  |  |  |
| Copper wire |  |  | 0.625 |  |  |  |  |  |  |
| Alumina |  | 0.003 |  |  |  |  |  |  |  |
| Epoxy resin |  | 0.002 |  |  |  |  |  |  |  |
| Fiberglass |  | 0.007 |  |  |  |  |  |  |  |
| PET |  | 0.030 |  |  |  |  |  |  |  |
| Zinc |  | 0.011 |  |  |  |  |  |  |  |
| Zinc oxide |  | 0.0002 |  |  |  |  |  |  |  |

|  | Empennage | Fuselage | Wing | Nacelle | Power electronics | Battery | Electric motors | Rotors |
| --- | --- | --- | --- | --- | --- | --- | --- | --- |
| Aluminum | 0.043 | 0.851 | 0.841 |  |  | 0.116 | 0.001 |  |
| Steel |  | 0.007 | 0.028 |  | 0.696 | 0.221 | 0.606 |  |
| CFRP | 0.711 | 0.092 | 0.077 | 0.400 |  |  |  |  |
| Titanium |  | 0.045 | 0.054 |  |  |  |  |  |
| GFRP | 0.246 | 0.005 |  |  |  |  |  |  |
| ASC-II |  |  |  |  |  |  |  | 0.500 |
| Copper |  |  |  |  |  | 0.070 | 0.084 |  |
| Al2024 |  |  |  | 0.150 |  |  |  | 0.500 |
| Nd-Fe-B |  |  |  |  |  |  | 0.033 |  |
| NCM811 |  |  |  |  |  | 0.250 |  |  |
| Binder |  |  |  |  |  | 0.009 |  |  |
| Ethylene Carbonate |  |  |  |  |  | 0.029 |  |  |
| Wrought aluminum |  |  |  |  | 0.064 |  |  |  |
| Graphite |  |  |  |  |  | 0.175 |  |  |
| Dimethyl Carbonate |  |  |  |  |  | 0.029 |  |  |
| LiPF6 |  |  |  |  |  | 0.010 |  |  |
| Ceramic |  |  |  |  |  | 0.0001 |  |  |
| PP |  |  |  |  | 0.011 | 0.008 |  |  |
| Thermal Insulation |  |  |  |  |  | 0.004 |  |  |
| Titanium alloy |  |  |  | 0.450 |  |  |  |  |
| Tin |  |  |  |  | 0.0003 |  |  |  |
| Magnet |  |  |  |  | 0.001 |  |  |  |
| Nylon |  |  |  |  | 0.034 |  |  |  |
| Acrylic acid |  |  |  |  | 0.011 |  |  |  |
| Cast aluminum |  |  |  |  |  |  | 0.277 |  |
| Copper wire |  |  |  |  | 0.162 |  |  |  |
| PET |  |  |  |  | 0.021 | 0.003 |  |  |
| Stainless steel |  |  |  |  |  | 0.042 |  |  |
| Ethylene Glycol |  |  |  |  |  | 0.023 |  |  |
| Electronic Parts |  |  |  |  |  | 0.007 |  |  |
| Polymer |  |  |  |  |  | 0.007 |  |  |

**Table S5**. Energy emission factors of China

| Parameter | Notation | Unit | Value | References |
| --- | --- | --- | --- | --- |
| Electricity emission factor | $EF_{e}$ | kg CO_2_/MJ | 0.16 | ^13,14^ |
| Gas emission factor | $EF_{g}$ |  | 0.07 |  |
| Heat & steam emission factor | $EF_{h}$ |  | 0.12 |  |
| Diesel emission factor | $EF_{d}$ |  | 0.07 |  |
| Kerosene emission factor | $EF_{k}$ |  | 0.07 |  |

**Table S6**. Material emission factors of China^13^

| $EF_{e}$ (kg CO_2_ eq./kWh) | 0.1 | 0.2 | 0.25 | 0.3 | 0.4 | 0.5 | 0.57 | 0.6 |
| --- | --- | --- | --- | --- | --- | --- | --- | --- |
| Aluminum | 3.58 | 5.03 | 5.76 | 6.48 | 7.93 | 9.38 | 10.40 | 10.83 |
| Steel | 10.20 | 10.26 | 10.29 | 10.33 | 10.39 | 10.45 | 10.50 | 10.52 |
| CFRP | 13.84 | 16.02 | 17.11 | 18.21 | 20.39 | 22.57 | 24.10 | 24.75 |
| Titanium | 22.79 | 26.60 | 28.50 | 30.41 | 34.22 | 38.03 | 40.70 | 41.84 |
| GFRP | 2.66 | 2.99 | 3.15 | 3.32 | 3.65 | 3.98 | 4.22 | 4.31 |
| ASC-II | 2.95 | 4.43 | 5.17 | 5.91 | 7.40 | 8.88 | 9.92 | 10.36 |
| Copper | 1.40 | 1.94 | 2.20 | 2.47 | 3.00 | 3.54 | 3.91 | 4.07 |
| Plastics | 2.52 | 2.66 | 2.72 | 2.79 | 2.93 | 3.07 | 3.17 | 3.21 |
| Steel alloy | 0.64 | 0.76 | 0.82 | 0.88 | 1.00 | 1.12 | 1.20 | 1.24 |
| Coated steel | 14.90 | 15.05 | 15.13 | 15.20 | 15.36 | 15.51 | 15.61 | 15.66 |
| RIMFG | 0.93 | 1.06 | 1.13 | 1.20 | 1.33 | 1.46 | 1.55 | 1.59 |
| Cast steel | 14.90 | 15.05 | 15.13 | 15.20 | 15.36 | 15.51 | 15.61 | 15.66 |
| Al2024 | 4.32 | 6.25 | 7.21 | 8.17 | 10.10 | 12.03 | 13.39 | 13.96 |
| Synthetic rubber | 2.96 | 3.04 | 3.08 | 3.11 | 3.19 | 3.26 | 3.32 | 3.34 |
| Nylon 6 | 3.64 | 3.83 | 3.93 | 4.03 | 4.22 | 4.42 | 4.55 | 4.61 |
| Viscose | 2.33 | 2.60 | 2.73 | 2.87 | 3.14 | 3.41 | 3.60 | 3.68 |
| Polyurethane | 1.60 | 1.72 | 1.78 | 1.84 | 1.96 | 2.08 | 2.16 | 2.20 |
| Paper | 0.00 | 0.00 | 0.00 | 0.00 | 0.00 | 0.00 | 0.00 | 0.00 |
| Halocarbon | 0.64 | 0.79 | 0.86 | 0.93 | 1.08 | 1.22 | 1.33 | 1.37 |
| Silicone | 25.58 | 37.84 | 43.96 | 50.09 | 62.35 | 74.61 | 83.23 | 86.87 |
| PVC | 1.18 | 1.32 | 1.38 | 1.45 | 1.58 | 1.71 | 1.80 | 1.84 |
| Polyurethane coated nylon | 3.39 | 3.63 | 3.75 | 3.87 | 4.12 | 4.36 | 4.54 | 4.61 |
| Nd-Fe-B | 8.35 | 10.10 | 10.97 | 11.85 | 13.60 | 15.34 | 16.57 | 17.09 |
| NCM811 | 13.24 | 15.08 | 16.00 | 16.92 | 18.76 | 20.60 | 21.90 | 22.44 |
| Nickel | 13.41 | 14.21 | 14.61 | 15.02 | 15.82 | 16.62 | 17.19 | 17.43 |
| Binder | 1.15 | 1.39 | 1.51 | 1.63 | 1.87 | 2.11 | 2.28 | 2.35 |
| Ethylene carbonate | 0.02 | 0.02 | 0.02 | 0.02 | 0.02 | 0.02 | 0.02 | 0.02 |
| Wrought aluminum | 3.58 | 5.03 | 5.76 | 6.48 | 7.93 | 9.38 | 10.40 | 10.83 |
| Graphite | 2.00 | 2.27 | 2.41 | 2.54 | 2.81 | 3.08 | 3.27 | 3.35 |
| Dimethyl carbonate | 0.09 | 0.09 | 0.10 | 0.10 | 0.10 | 0.10 | 0.10 | 0.10 |
| LiPF6 | 2.16 | 4.29 | 5.35 | 6.42 | 8.55 | 10.67 | 12.17 | 12.80 |
| Ceramic | 0.61 | 0.63 | 0.63 | 0.64 | 0.66 | 0.68 | 0.69 | 0.70 |
| PP | 4.69 | 4.79 | 4.84 | 4.88 | 4.98 | 5.07 | 5.14 | 5.17 |
| Thermal insulation | 0.35 | 0.35 | 0.36 | 0.36 | 0.36 | 0.37 | 0.37 | 0.38 |
| Titanium alloy | 28.76 | 34.85 | 37.90 | 40.94 | 47.03 | 53.12 | 57.41 | 59.21 |
| Tin | 10.20 | 10.26 | 10.29 | 10.33 | 10.39 | 10.45 | 10.50 | 10.52 |
| Magnet | 8.35 | 10.10 | 10.97 | 11.85 | 13.60 | 15.34 | 16.57 | 17.09 |
| Nylon | 3.64 | 3.83 | 3.93 | 4.03 | 4.22 | 4.42 | 4.55 | 4.61 |
| Acrylic acid | 0.16 | 0.19 | 0.21 | 0.22 | 0.26 | 0.29 | 0.32 | 0.33 |
| Cast aluminum | 2.38 | 3.29 | 3.74 | 4.19 | 5.09 | 5.99 | 6.62 | 6.89 |
| Copper wire | 1.40 | 1.94 | 2.20 | 2.47 | 3.00 | 3.54 | 3.91 | 4.07 |
| Alumina | 0.61 | 0.63 | 0.63 | 0.64 | 0.66 | 0.68 | 0.69 | 0.70 |
| Epoxy resin | 4.35 | 4.54 | 4.63 | 4.73 | 4.92 | 5.12 | 5.25 | 5.31 |
| Fiberglass | 0.93 | 1.06 | 1.13 | 1.20 | 1.33 | 1.46 | 1.55 | 1.59 |
| PET | 1.52 | 1.68 | 1.75 | 1.83 | 1.99 | 2.15 | 2.26 | 2.31 |
| Zinc | 0.63 | 1.13 | 1.38 | 1.62 | 2.12 | 2.61 | 2.96 | 3.11 |
| Zinc oxide | 3.98 | 4.38 | 4.58 | 4.78 | 5.18 | 5.58 | 5.86 | 5.98 |
| Stainless steel | 0.64 | 0.76 | 0.82 | 0.88 | 1.00 | 1.12 | 1.20 | 1.24 |
| Ethylene glycol | 2.66 | 2.71 | 2.73 | 2.75 | 2.79 | 2.84 | 2.87 | 2.88 |
| Electronic parts | 9.45 | 12.99 | 14.77 | 16.54 | 20.08 | 23.63 | 26.12 | 27.17 |
| Polymer | 0.64 | 0.79 | 0.86 | 0.93 | 1.08 | 1.22 | 1.33 | 1.37 |

* Material emission factor unit: kg CO_2_ eq./kg

**Supplemental References**

1 Stoll, A. Analysis and Full Scale Testing of the Joby S4 Propulsion System. (Joby Aviation, 2015).

2 Kasliwal, A. *et al.* Role of flying cars in sustainable mobility. *Nat Commun* **10**, 1555 (2019).

3 Yang, X.-G., Liu, T., Ge, S., Rountree, E. & Wang, C.-Y. Challenges and key requirements of batteries for electric vertical takeoff and landing aircraft. *Joule* **5**, 1644-1659 (2021).

4 Zhang, Y. *et al.* Progress and key technologies of flying cars. *Journal of Automotive Safety and Energy* **11**, 1-16 (2020).

5 Liu, M. *et al.* Lifecycle greenhouse gas emissions and energy cost analysis of flying cars with three different propulsion systems. *Journal of Cleaner Production* **331** (2022).

6 Liu, M. *et al.* Flying cars economically favor battery electric over fuel cell and internal combustion engine. *PNAS Nexus* **2**, 1-13 (2023).

7 Liu, M. *et al.* CO2 emissions from electric flying cars: Impacts from battery specific energy and grid emission factor. *eTransportation* **13** (2022).

8 Luo, Y., Qian, Y., Zeng, Z. & Zhang, Y. Simulation and analysis of operating characteristics of power battery for flying car utilization. *eTransportation* **8** (2021).

9 Schwinn, D., Weiand, P., Buchwald, M. & Schmid, M. Rotorcraft Fuselage Weight Assessment in Early Design Stages. (2018).

10 André, N. & Hajek, M. Robust Environmental Life Cycle Assessment of Electric VTOL Concepts for Urban Air Mobility. *AIAA Aviation 2019 Forum* (2019).

11 Thonemann, N. *et al.* Prospective life cycle inventory datasets for conventional and hybrid-electric aircraft technologies. *Journal of Cleaner Production* **434** (2024).

12 Vivalda, P. *Aircraft Life Cycle Assessment: the implementation of a tool and three case studies*, Politecnico di Torino, (2023).

13 Argonne National Laboratory. Summary of Expansions and Updates in R&D GREET® 2023. <https://greet.anl.gov/publication-greet-2023-summary> (2023).

14 Ministry of Ecology and Environment of the People’s Republic of China. *Notice on the management of greenhouse gas emission reporting of enterprises in the power generation industry from 2023 to 2025*, <<https://www.mee.gov.cn/xxgk2018/xxgk/xxgk06/202302/t20230207_1015569.html>> (2023).
